# Mapping the evidence about the natural history of acute infections commonly seen in primary care and managed with antibiotics: a scoping review

**DOI:** 10.1186/s12879-024-09526-3

**Published:** 2024-07-23

**Authors:** Kwame Peprah Boaitey, Mina Bakhit, Tammy C Hoffmann

**Affiliations:** Institute for Evidence-Based Healthcare, Faculty of Health Sciences and Medicine, Bond University, 14 University Dr, Robina, QLD 4226 Australia

**Keywords:** Natural history, Acute infections, Primary care, Respiratory tract infections, Antibiotic stewardship

## Abstract

**Background:**

Knowing the natural history of acute infections in primary care, defined as the course of a disease over time in the absence of specific therapy or treatment, can inform clinicians’ and patients’ expectations about illness recovery, but this evidence is fragmented across the literature. This scoping review aimed to map existing research and research gaps relevant to the natural history of acute infections.

**Methods:**

We searched MEDLINE, Embase and CENTRAL using a 2-phase hierarchical search approach. In Phase A, we focused on identifying systematic reviews synthesising natural history data for eligible infections (acute respiratory, urinary, and skin and soft tissue) and systematic reviews of treatment effectiveness (of RCTs with placebo or no treatment arm, or cohort studies). For infections without existing reviews, in Phase B, we searched for primary studies (placebo-controlled RCTs or cohort studies). Two reviewers independently screened and extracted the data (study characteristics, outcome data - e.g., symptom duration, proportion with resolution at various time points).

**Results:**

We identified 40 systematic reviews, reporting on 45 infections, most commonly (90%) respiratory tract infections. Six (15%) of these aimed to synthesise natural history information. Most reviews reported the proportion of participants with symptom resolution at various time point/s, with 58% providing data on mean symptom duration. Recovery data show the spontaneous resolution of some infections in some people. We found no eligible studies for cellulitis, ecthyma, carbuncle, and erysipelas.

**Conclusions:**

Our review has shown that natural history evidence exists for many common acute infections. It can be utilised by clinicians in implementing patient-centred antibiotic stewardship strategies in primary care. Future research should focus on generating natural history evidence for skin and soft tissue infections and urinary tract infections.

**Supplementary Information:**

The online version contains supplementary material available at 10.1186/s12879-024-09526-3.

## Background

Antibiotic resistance is a global public health emergency threatening our ability to manage infections [1]. The vast tonnage of antibiotic use is a major driver of resistance, with most overuse in primary care [2–4]. Most antibiotics are overused in self-limiting acute infections [5, 6], such as various acute respiratory infections, where antibiotics have a delicate benefit-harm trade-off [7, 8]. Many patients and clinicians believe antibiotics are always necessary for these infections, overestimating their benefits and underestimating harms [9, 10].

Antibiotic stewardship strategies are a public health priority [1]. Strategies that can be implemented individually in primary care include delayed prescribing [11] and shared decision making [12]. Central to these is prescribers knowing and communicating the natural history of common acute infections [13], that is, the course of a disease over time in the absence of specific therapy or treatment [14], which for acute infections is typically antibiotics. Knowledge of the likely duration of an infection may facilitate informed decision-making and decrease patients’ expectations of and requests for antibiotics [13, 15].

Given the importance of natural history knowledge for clinical decision-making and its relevance to primary care antibiotic stewardship strategies, it has been a surprisingly neglected area of research [16]. Evidence about natural history appears to be fragmented across the literature, with no existing databases or repositories of synthesised information, like there is for treatment evidence [16]. Awareness of existing research on self-limiting infections and its gaps can inform future research agendas. This scoping review aims to identify existing research and research gaps relevant to the natural history of acute infections commonly seen in primary care (such as acute respiratory, urinary, and skin and soft tissue infections) and often managed with antibiotics.

## Methods

The review followed the PRISMA-ScR checklist (Preferred Reporting Items for Systematic Reviews and Meta-Analyses Extension for Scoping Reviews) [17]. The protocol was registered in the Open Science Framework [18].

### Information sources and search strategy

We searched MEDLINE, Embase, and Cochrane CENTRAL databases from inception to February 2022, with no language restriction. We used a hierarchical search approach, starting with Phase A: identifying (i) eligible systematic reviews that had the primary aim of synthesising the natural history of eligible infections and (ii) systematic reviews of studies (randomised controlled trials (RCT) with a placebo or no treatment arm; or prospective cohort studies) that studied the effectiveness of antibiotics or other treatments. In Phase B, we searched for eligible primary studies (prospective cohort studies and RCTs with placebo arm) of infections for which no eligible systematic reviews were identified in Phase A. See Additional Box 1 for the search strategies.

The searches were conducted in MEDLINE using free-text words and MeSH terms. The search string was translated into other database platforms using Polyglot Search Translator with the help of an information specialist (Additional Box 2 for MEDLINE search strategy) [19]. We also screened 120 records identified in a previous systematic review of the reporting of natural history information in clinical practice guidelines [20].

### Inclusion and exclusion criteria

Study design eligibility was as described in the above section. We included reviews of patients from primary and ambulatory care settings of any age with any of these infection categories: acute respiratory infection (ARI), uncomplicated urinary tract infection (UTI), skin and soft tissue infection (SSTI) - see Additional Box 3 for eligible illnesses within each category. Primary and ambulatory care settings were defined as care provided to patients at their first encounter with the health system, including general practice, out-of-hour services, outpatient clinics, paediatric clinics, and emergency departments. To be included, studies must have reported outcome data on the duration of symptoms and/or the proportion of participants with symptom resolution at any time point/s.

### Selection of sources of evidence and data extraction

Two reviewers independently screened titles and abstracts, then the full text of potentially eligible records. Disagreements were resolved through discussion or third reviewer consultation. Potentially eligible non-English articles were translated using Google Translate.

Two reviewers independently extracted data, including study design, population characteristics, and eligible outcomes, using a custom-designed data extraction form. The form was piloted on five randomly selected reviews.

We extracted the duration of symptoms and/or the proportion of control group participants who experienced symptom resolution or worsening at any time point/s as reported in the included reviews. In reviews where only some of the included trials met this scoping review’s eligibility criteria, we extracted data from only the eligible trials (those with a placebo or no treatment comparison).

### Synthesis of results

We calculated descriptive statistics using Microsoft Excel 365® and created an evidence map of eligible reviews for each infection. For each infection, we report the mean or median duration of infection as reported in the reviews and present scatter plots of the proportion of the control group participants who experienced symptom resolution at reported time points.

## Results

**Fig. 1:**
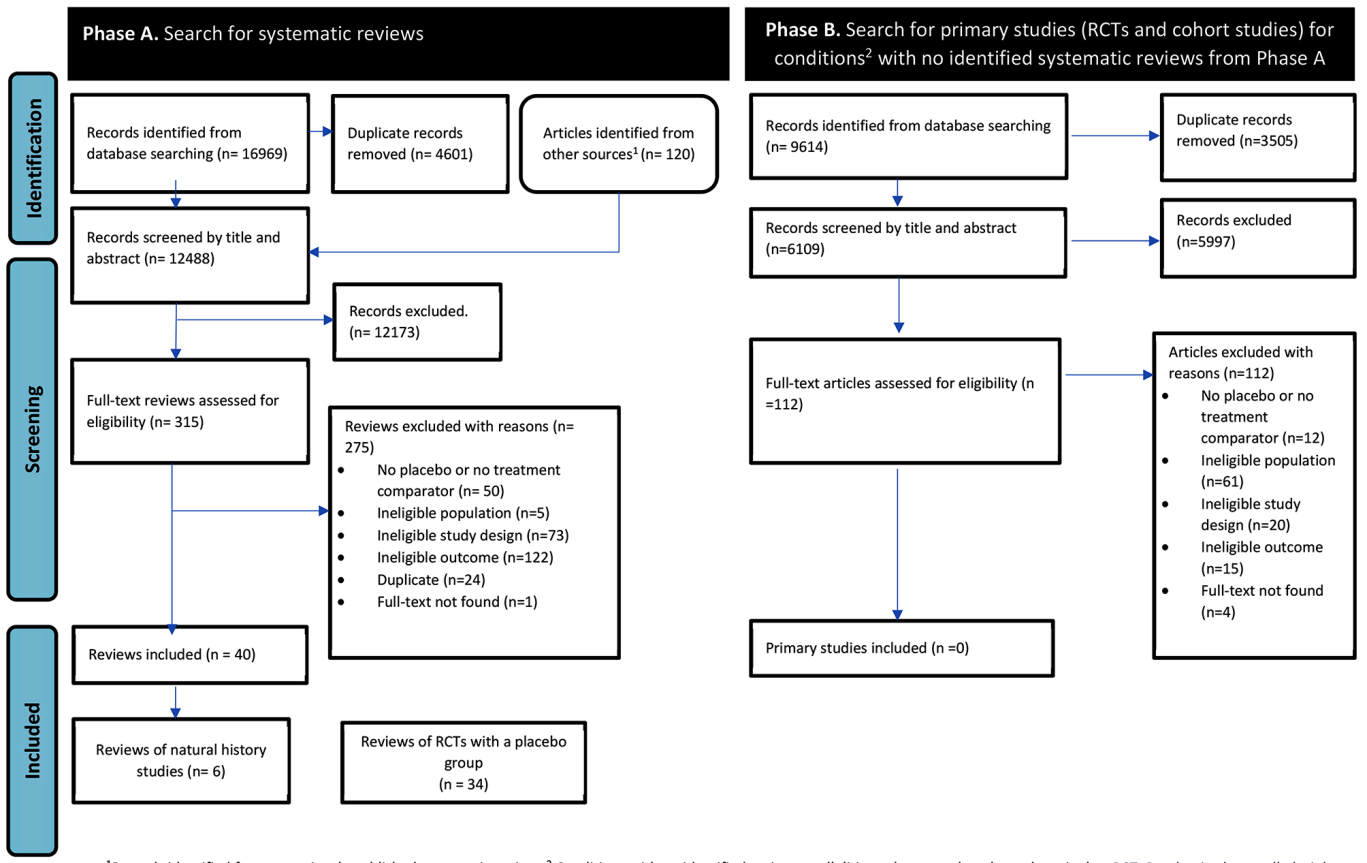
PRISMA diagram. ^1^Records identified from a previously published systematic review: ^2^Conditions with no identified reviews: cellulitis, ecthyma, carbuncle, and erysipelas. *RCT: Randomised controlled trials*

Phase A: Our database search for systematic reviews identified 16,969 records, of which 4,601 were duplicates. From a systematic review [20] of guidelines’ reporting of natural history information, we added 120 records to screen. We excluded 12,173 records after title and abstract screening and assessed the full text of 315 reviews. We included 40 eligible reviews, reporting 45 infections (two reported multiple infections) [13, 21]. See Fig. 1 for PRISMA flow diagram and Additional Tables 1 and 2 for the included and excluded reviews with reasons.

Phase B: We searched for primary studies for conditions with no eligible reviews identified in Phase A (cellulitis, ecthyma, carbuncles, erysipelas). Of 9614 records, 3505 duplicates were removed, and we screened 6109 titles and abstracts and 112 full texts. No eligible studies were identified. See Additional Table 3 for the list of excluded studies with reasons.

### Characteristics of included reviews

Most (90%, *n* = 36) of the systematic reviews addressed Acute Respiratory Infections (ARIs), with three about Skin and Soft Tissue infections (SSTIs) [22, 23] and one review about Urinary Tract Infections (UTIs) [15]. Additional Table 4 shows the characteristics of the included reviews.

Of the 40 included reviews, six (15%) aimed to synthesise natural history information [13, 15, 21, 24–26]. Of these, four included participants from cohort and observational studies as well as those from placebo or no treatment arms [13, 21, 24, 25], and two included multiple ARIs [13, 21]. The remaining 34 reviews were treatment effectiveness reviews of RCTs, with some trials using a placebo or no-treatment group. Half of the reviews (*n* = 20) were published between 2011 and 2015. Most reviews (70%, *n* = 28) included adults and children as participants. The number of studies included in the reviews ranged between 1 and 34, with the reviews’ total sample size ranging between 146 and 11,077 participants.

### Reviews providing information about the natural history of acute infections

**Fig. 2:**
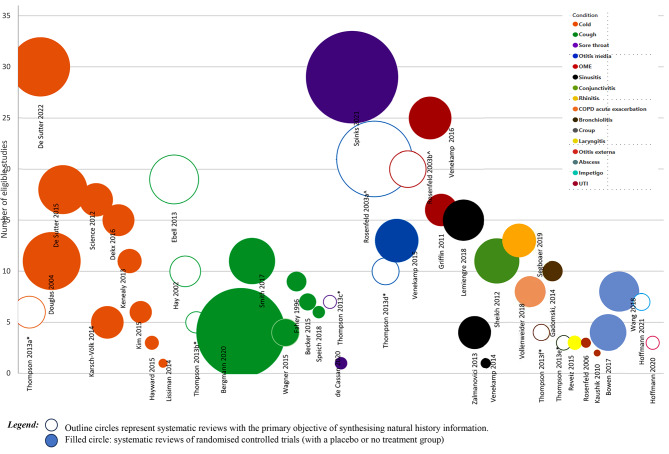
Evidence map of reviews containing natural history information about acute infections. OME: Acute otitis media with effusion, UTI: Urinary tract infection. X-axis: Systematic reviews identified for each condition; Y-axis: The number of eligible primary studies reported in each systematic review with a placebo or no treatment arm for randomised controlled trails. The bubble size reflects the number of studies included in the identified reviews that contributed natural history information. Thompson 2013*a, b, c, d, e, and f is a review reporting multiple respiratory tract conditions (a*= common cold, b*=cough, c*=sore throat, d*= otitis media, e*= bronchiolitis, and f*= croup), Rosenfeld 2003a^, and b^ included both otitis media and otitis media with effusion, respectively

See Additional Table 5 for the natural history data reported in the included reviews. Figure 2 presents the evidence map, grouped by condition, the number of reviews for each condition, and whether the review’s focus was synthesising natural history information.

The condition with the largest reported number of reviews was the common cold (*n* = 11), of which all but one was treatment effectiveness reviews. The sample size in the included reviews ranged between 146 and 6304 participants.

The condition with the second highest number of reported reviews (*n* = 9) was acute cough, with three reviews aiming to synthesise the natural history data [13, 24, 25]. The sample size of review participants ranged between 274 and 14,289.

Data about acute sinusitis were reported by four reviews, with sample sizes ranging from 1133 to 3057 participants. Three reviews provided natural history data about sore throat (one with an aim to synthesise natural history information [13]), with sample sizes ranging between 277 and 15,337).

Three reviews reported on acute otitis media data [8, 13, 21]. Three [21, 27, 28] reviews of participants ≤ 18 years of age were of otitis media with effusion, with one of these [21] aiming to synthesise natural history data. Two reviews reported data on the natural history of otitis externa [29, 30] and included participants of any age, with a sample size ranging between 3289 and 3382.

Data on bronchiolitis were reported by two reviews [13, 31]. One review [31] included 30 RCTs of infants ≤ 24 months with bronchiolitis, whereas the other review [13] reported data as part of a review reporting multiple ARI conditions and included 4 studies of bronchiolitis.

There was only one review for each of the following conditions: conjunctivitis [32] (11 RCTs, 3673 participants), rhinitis [33] (natural history data in four of the 34 RCTs, 2045 participants), croup [13] (natural history data in three RCTs, 415 participants), laryngitis [34] (natural history data in three RCTs, 351 participants), and acute exacerbation of COPD [35] (natural history data in eight RCTs, 1722 participants).

Only one review addressed UTIs [15] and focussed on synthesising natural history information for uncomplicated UTIs in women (natural history data from three RCTs, 346 participants). One review of impetigo [26], with a natural history focus, synthesised data from seven RCTs (*n* = 557). Two reviews [22, 23] reported data about the resolution of abscesses after excision and drainage (4 RCTs, 2405 participants; eight RCTs, 2890 participants).

### Natural history information available in the included reviews

**Fig. 3:**
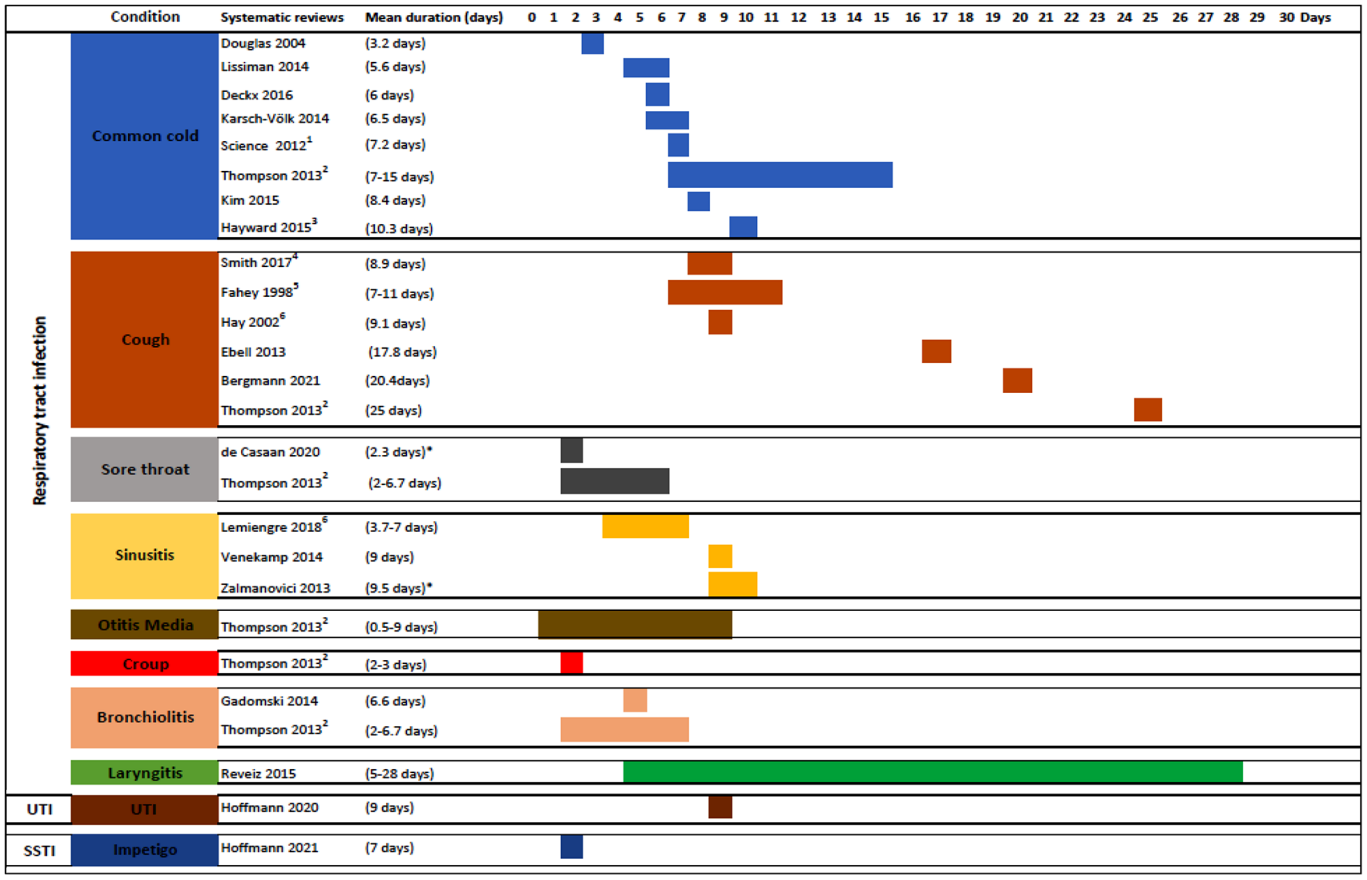
The mean duration of symptoms (unless otherwise specified) of the various conditions as reported in the systematic reviews. **UTI:** Urinary tract infection (uncomplicated), **SSTI: **Skin and Soft Tissue Infection ^1^Science 2021: Outcome data calculated from pooled analysis of data presented for both adult and children. ^2^Thompson 2013: This review reported data for multiple respiratory infections (reported separately). Outcomes were reported as the time point for when 90% of participants recovered from symptoms. ^3^Hayward 2015: Outcome data reported as the time-lapse to symptom resolution. ^4^Smith 2017: Outcome calculated from pooled analysis (Analysis 4.1; 6 studies, 1162 participants). The outcome was reported as the mean number of days of cough. ^5^Fahay 1998: Outcome reported from the final day of clinical assessment. ^6^Outcome data reported narratively. Duration reported from physician outcome measure with complication. *Median duration of symptoms

Figure 3 summarises the mean duration of each condition as reported in reviews. The mean duration of symptoms was reported in 45 (58%) of the conditions.

Figures 4–16 summarise the proportion of participants in the control group with symptom resolution at various time points.

**Fig. 4:**
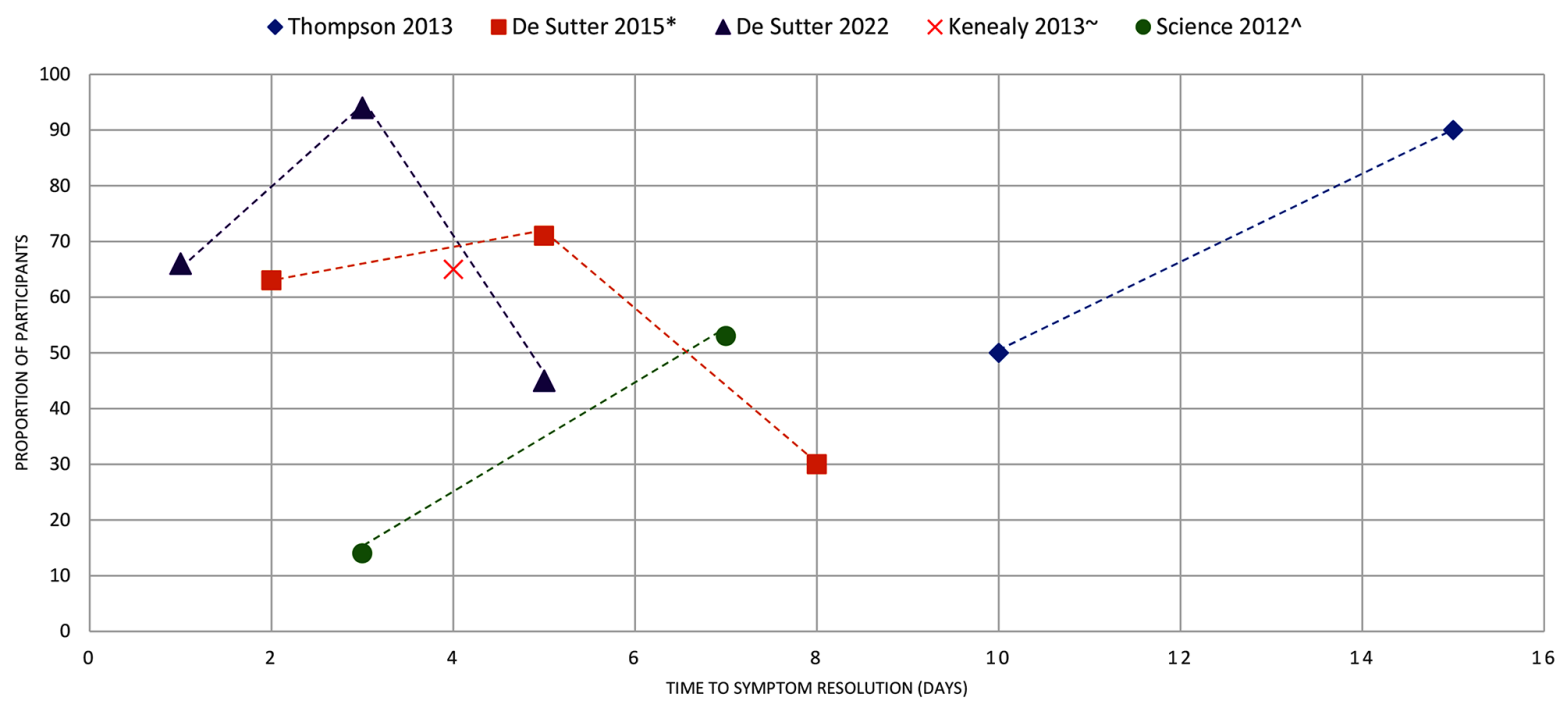
COMMON COLD: the proportion of participants with symptom resolution at various time points. De Sutter 2015*: The outcome data were reported as improvements in symptoms score. We calculated the proportions from the forest plots. Kenealy 2013~: The outcome data were reported as proportion of participants with persistent symptoms, which we used to calculate the proportion with symptoms resolution. The review reported outcome data on days 1-7. We assumed the median point for the time to symptom resolution. Science 2012^: We calculated the proportion of participants with symptoms resolution from the number of participants with symptoms (pooled analysis, 17 studies, 858 participants)

**Fig. 5:**
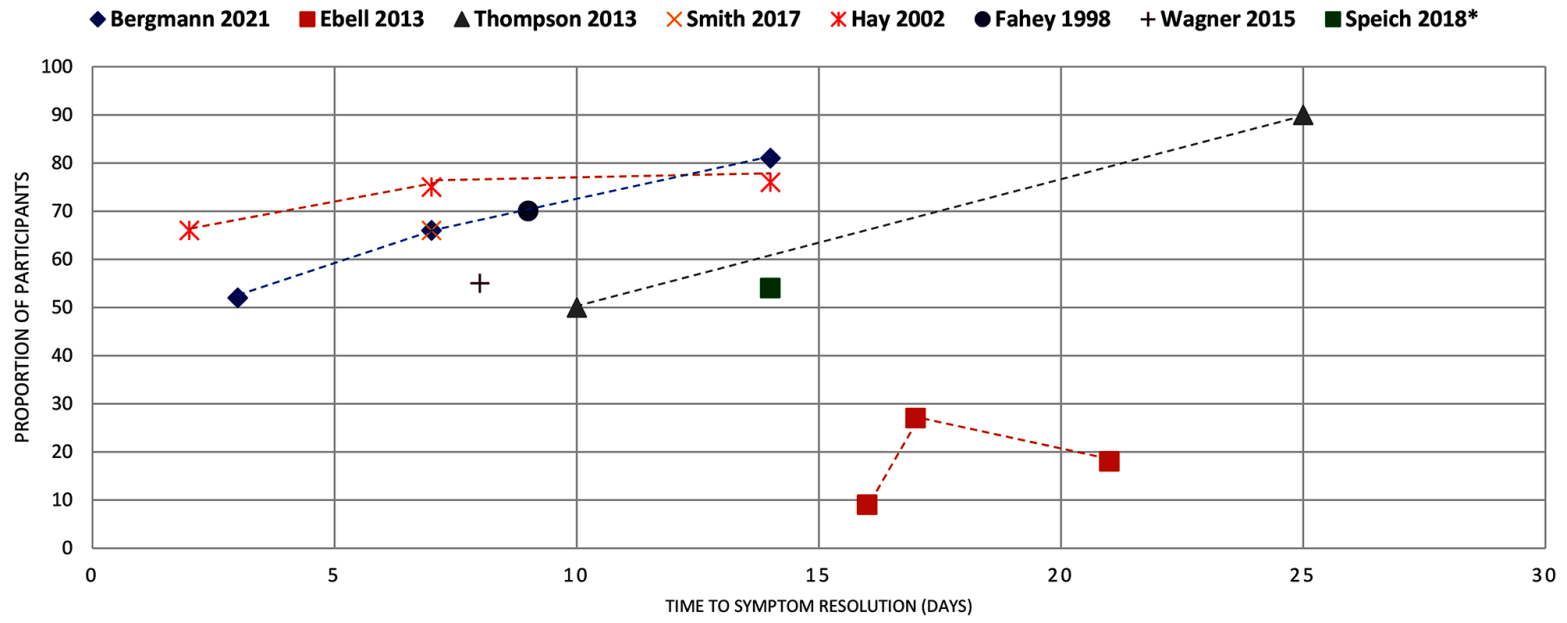
COUGH: the proportion of participants with symptom resolution at various time points. De Sutter 2015*: The outcome data were reported as improvements in symptoms score. We calculated the proportions from the forest plots. Kenealy 2013~: The outcome data were reported as proportion of participants with persistent symptoms, which we used to calculate the proportion with symptoms resolution. The review reported outcome data on days 1-7. We assumed the median point for the time to symptom resolution.Science 2012^: We calculated the proportion of participants with symptoms resolution from the number of participants with symptoms (pooled analysis, 17 studies, 858 participants). Bergmann 2021: We plotted the proportion of participants with clinical improvement (4 studies, 1016 participants). Ebell 2013: Outcome data were calculated by subtracting the percentage of participants with cough to attain the number of participants symptom resolution. Smith 2017: The time point for proportion without symptoms is assumed from the trial with the largest sample size in the review (Little 2013). Outcome data were calculated from the pooled analysis (11 studies, 1277 participants). Wagner 2015: We used the timepoint from one trial (Kammerich 2017), which is the only included study that provided a timepoint for assessment. Outcome data were calculated from the pooled analysis (2 studies, 395 participants). Speich 2018*: Patients with subacute cough, outcome data reported by one study (Ponsioen 2005)

**Fig. 6:**
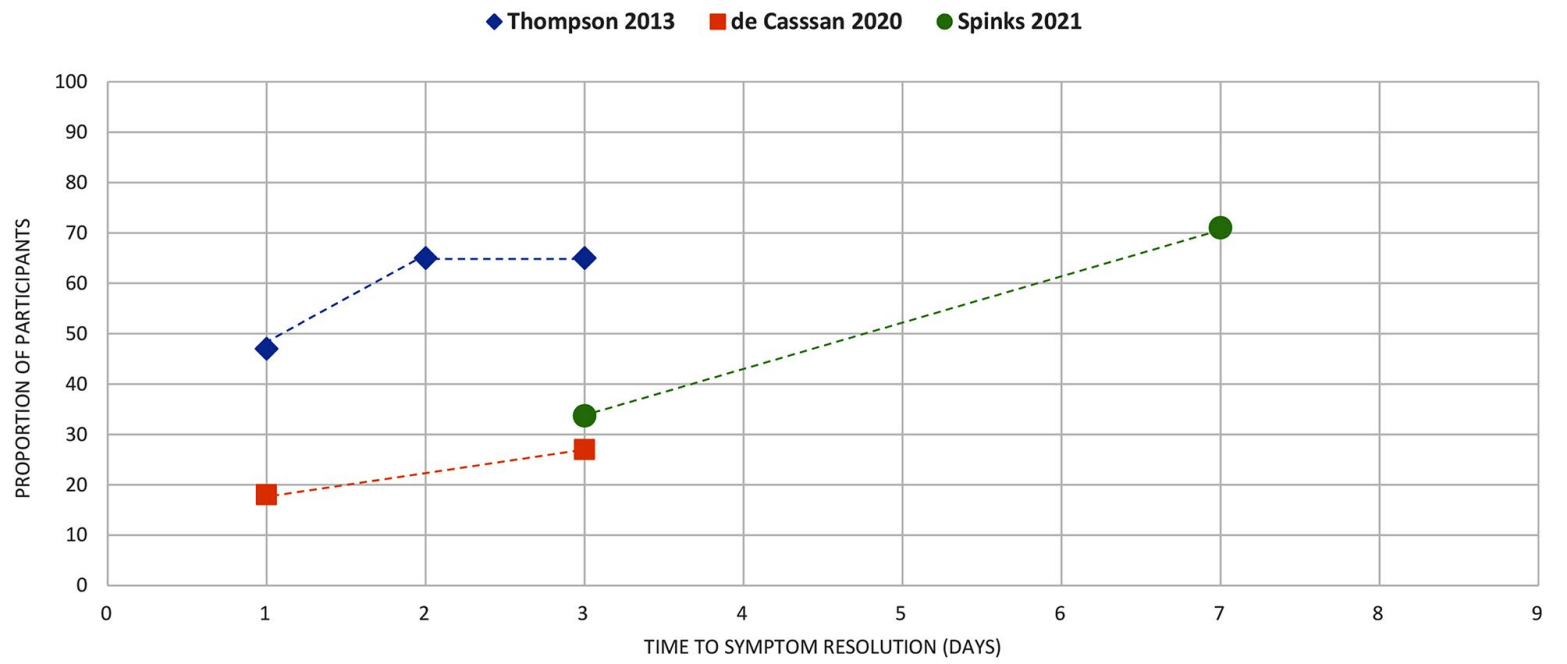
SORE THROAT: the proportion of participants with symptom resolution at various time points. Thompson 2013: The review reported the proportion of participants who were symptomatic at the specified time point. We calculated the outcome from the proportion with symptoms. de Cassan 2020: The proportion of participants with complete resolution of pain. Spinks 2021: The outcome data at day 3 was reported as the proportion of participants with symptoms, which was used to calculate the proportion of participants with symptom resolution

**Fig. 7:**
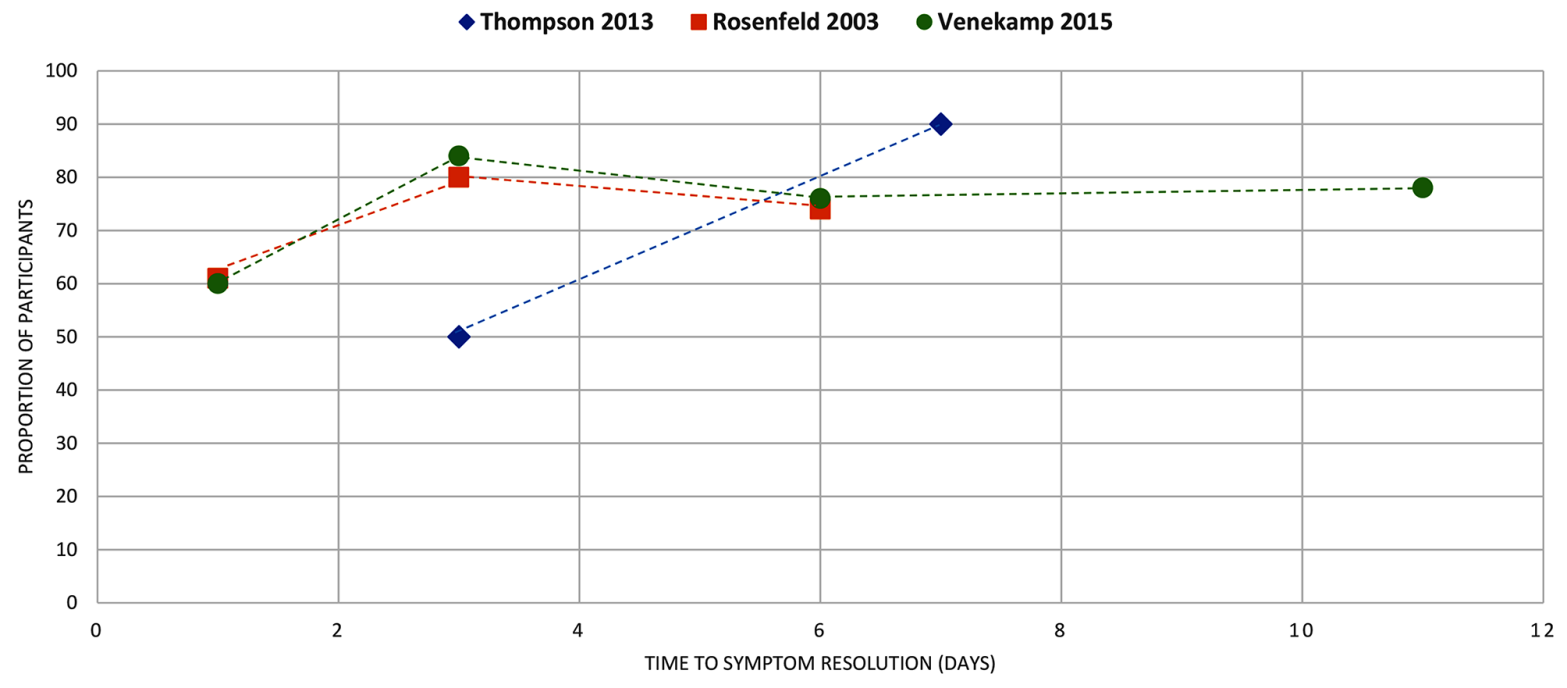
ACUTE OTITIS MEDIA: the proportion of participants with symptom resolution at various time points

**Fig. 8:**
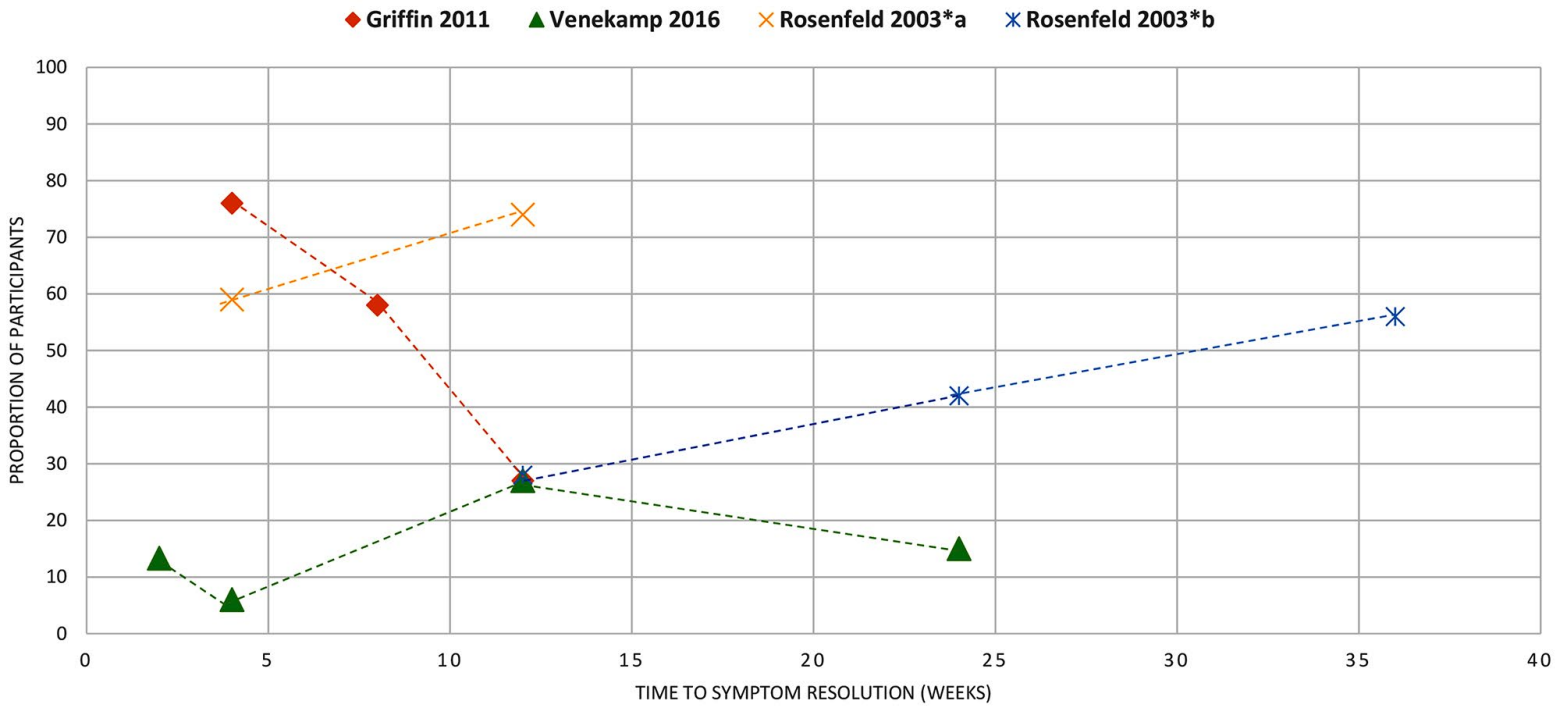
OTITIS MEDIA WITH EFFUSION: the proportion of participants with symptom resolution at various time points. Griffin 2011: Plotted data represents placebo participants from the RCT of antihistamine + decongestant combination in the review. Refer to Supplementary Table V for further details. Rosenfeld 2003*a: participants with untreated otitis media with effusion; Rosenfeld 2003*b: otitis media with effusion of unknown duration. Venekamp 2016: As reported in the review, only 52% of placebo participants received a true placebo, others received treatment of unproven efficacy (this was not clearly defined in the review)

**Fig. 9:**
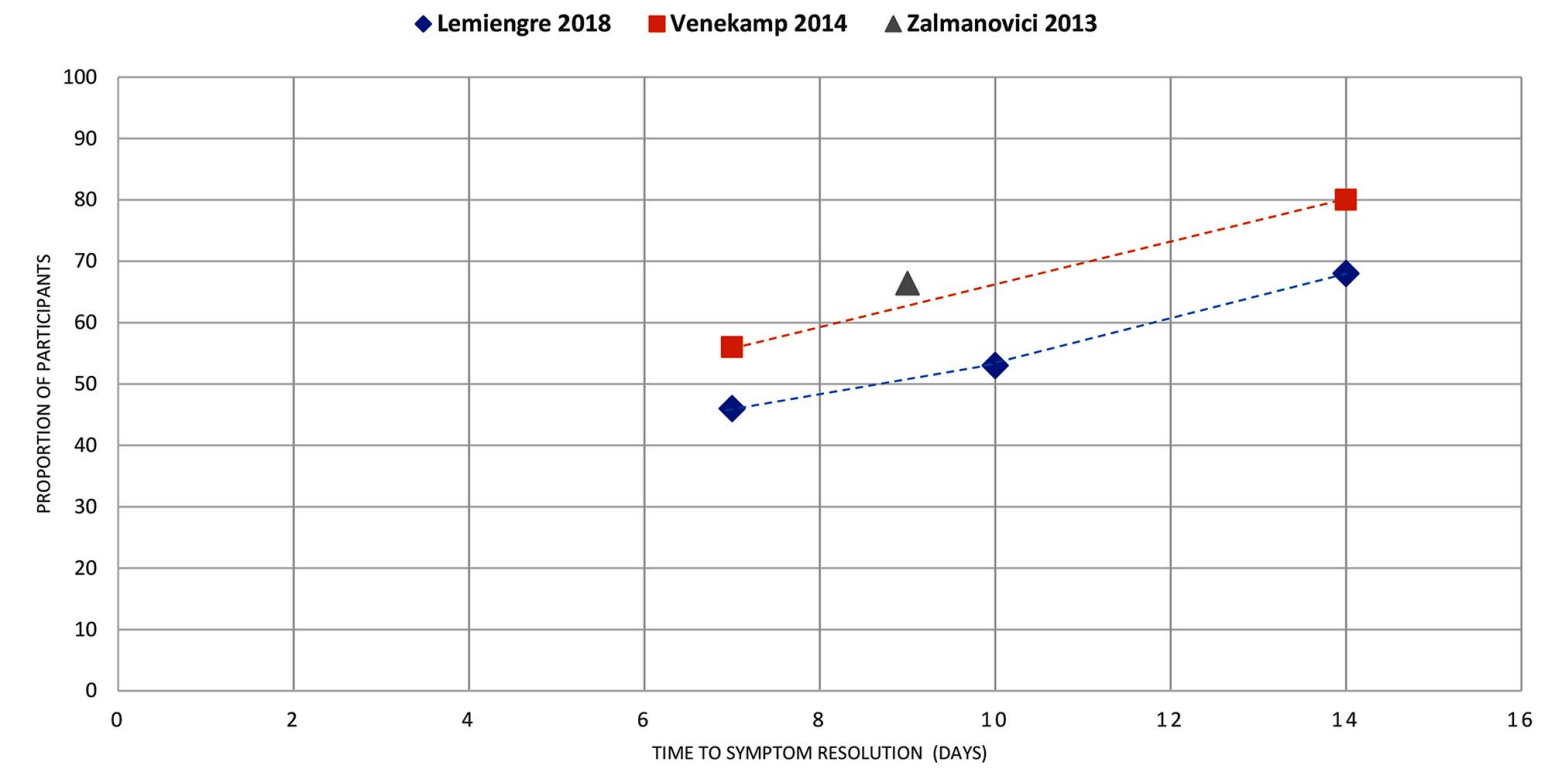
ACUTE SINUSITIS: the proportion of participants with symptom resolution at various time points. Griffin 2011: Plotted data represents placebo participants from the RCT of antihistamine + decongestant combination in the review. Refer to Supplementary Table V for further details. Rosenfeld 2003*a: participants with untreated otitis media with effusion; Rosenfeld 2003*b: otitis media with effusion of unknown duration. Venekamp 2016: As reported in the review, only 52% of placebo participants received a true placebo, others received treatment of unproven efficacy (this was not clearly defined in the review). Lemiengre 2018: Outcome data as reported (pooled analysis, 11 studies, 603 participants). Venekamp 2014: Outcome data as reported (narratively, 1 study, 86 participants). Zalmanovici 2013: Outcome data as reported (pooled analysis, 3 studies, 624 participants). We used the median time to clinical success reported in one study (Dolor 2001) for the datapoint

**Fig. 10:**
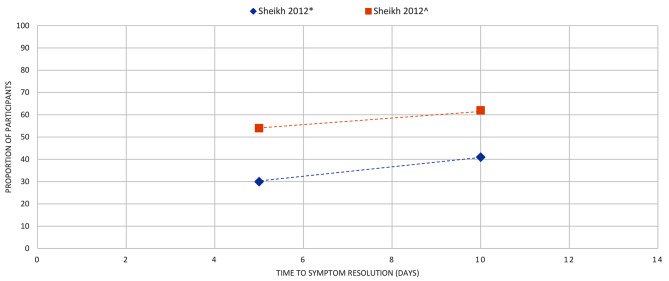
CONJUNCTIVITIS: the proportion of participants with symptom resolution at various time points (in days). Sheikh 2012*: Outcome data for clinical remission. Sheikh 2012^: Outcome data for biological remission

**Fig. 11:**
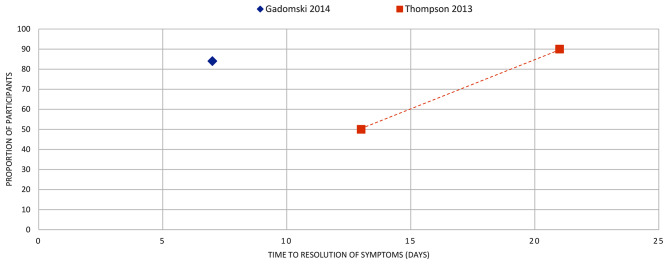
BRONCHIOLITIS: the proportion of participants with symptom resolution at various time points. Gadomski 2014: We used the pooled mean duration as the time point to resolution of symptoms. Thompson 2013: The proportion of participants symptom free at day 21 was an estimated proportion reported narratively in the review

**Fig. 12:**
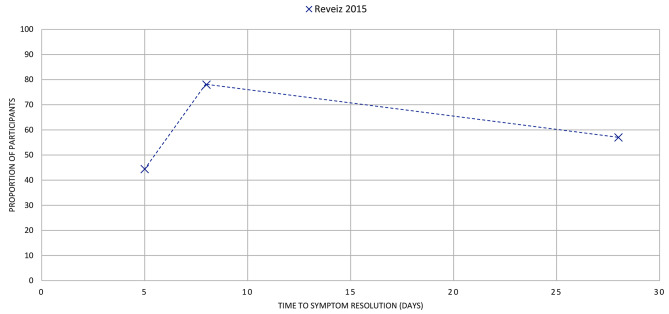
LARYNGITIS: the proportion of participants with symptom resolution at various time points. Outcome data represent data from placebo participants of fusafungine + clarithromycin combination. The review reported additional data for fusafungine alone + placebo and Erythromycin alone + placebo in the review. Refer to Supplementary Table V for further details

**Fig. 13:**
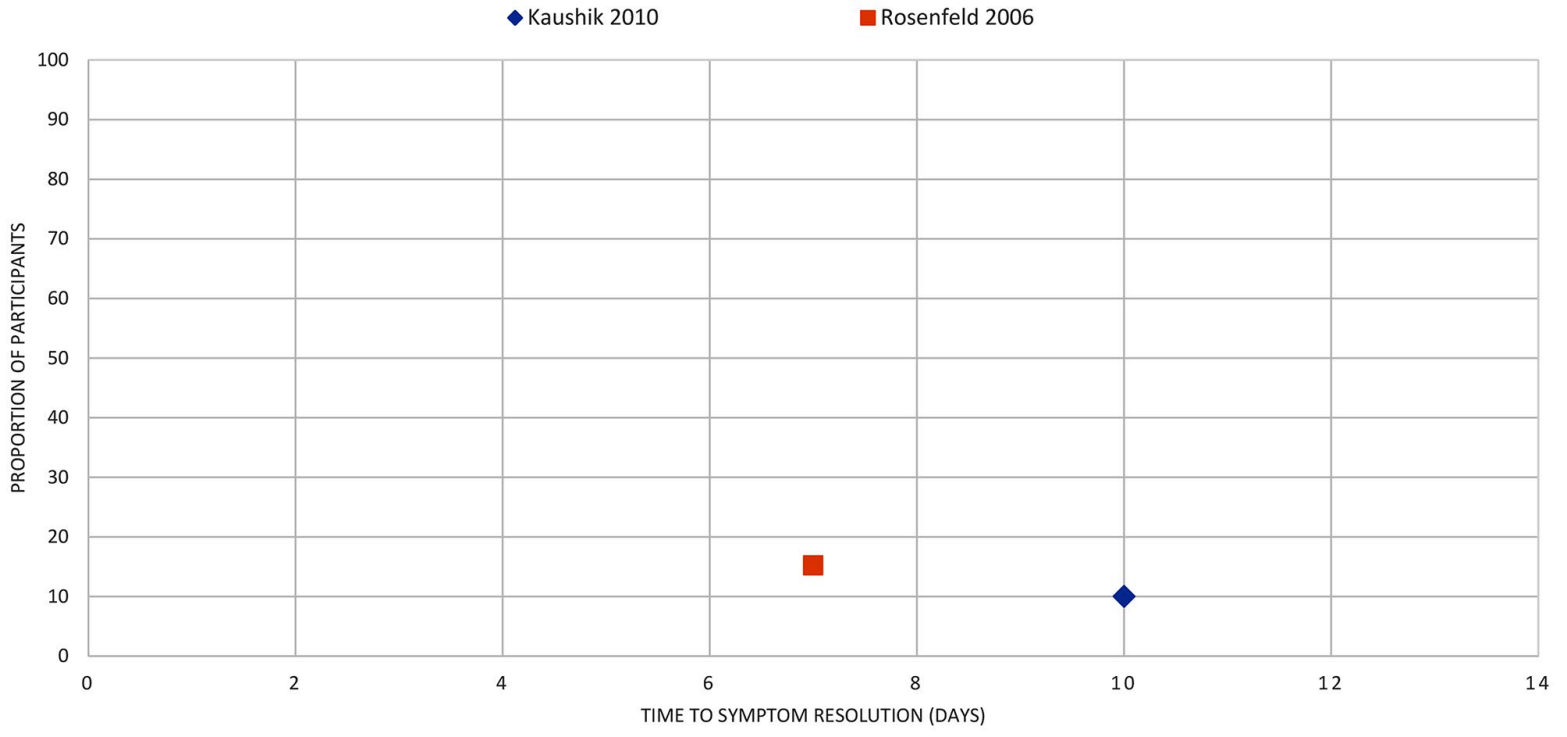
OTITIS EXTERNA: the proportion of participants with symptom resolution at various time points. Kaushik 2010: Outcome data as reported narratively (20 participants). Rosenfeld 2006: Outcome data calculated (pooled analysis, 2 studies, 46 participants)

**Fig. 14:**
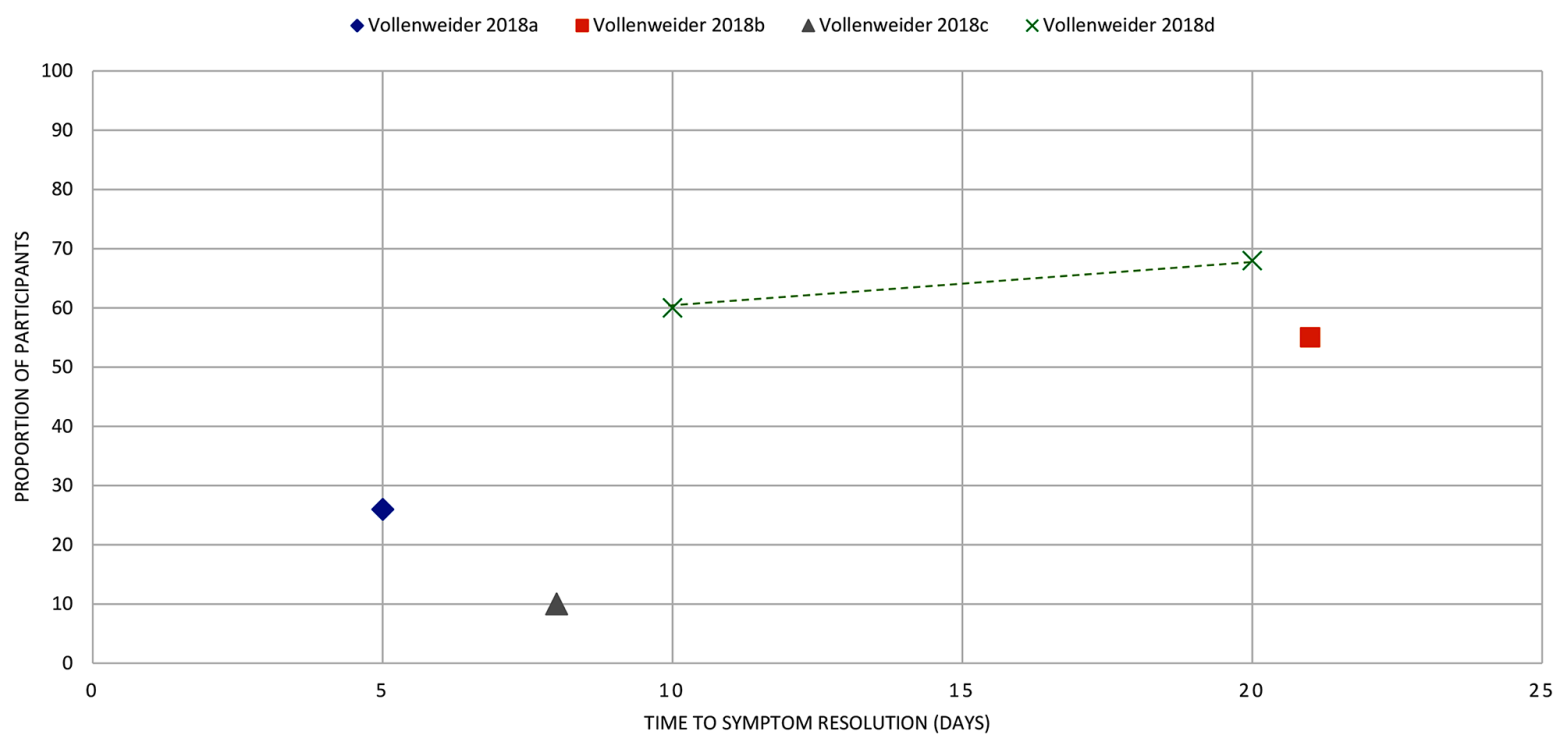
ACUTE EXACERBATION OF COPD: the proportion of participants with symptom resolution at various time points. Vollenweider 2008a, b, c, d represents reported data from individual studies extracted from the review. (a): data from Allegra 1991, (b): Anthonisen 1987, (c): Jorgensen 1992, (d): Llor 2012

**Fig. 15:**
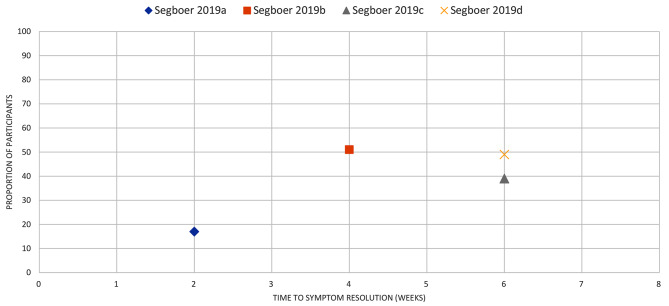
ACUTE RHINITIS: the proportion of participants with symptom resolution at various time points. Segboer 2019 a, b, c, d represents reported data from individual studies extracted from the review. (**a**): data from Day 1990, (**b**): Tuekeltaub 1982, (**c**): Schulz 1978, (**d**): Lundblad 2001

**Fig. 16:**
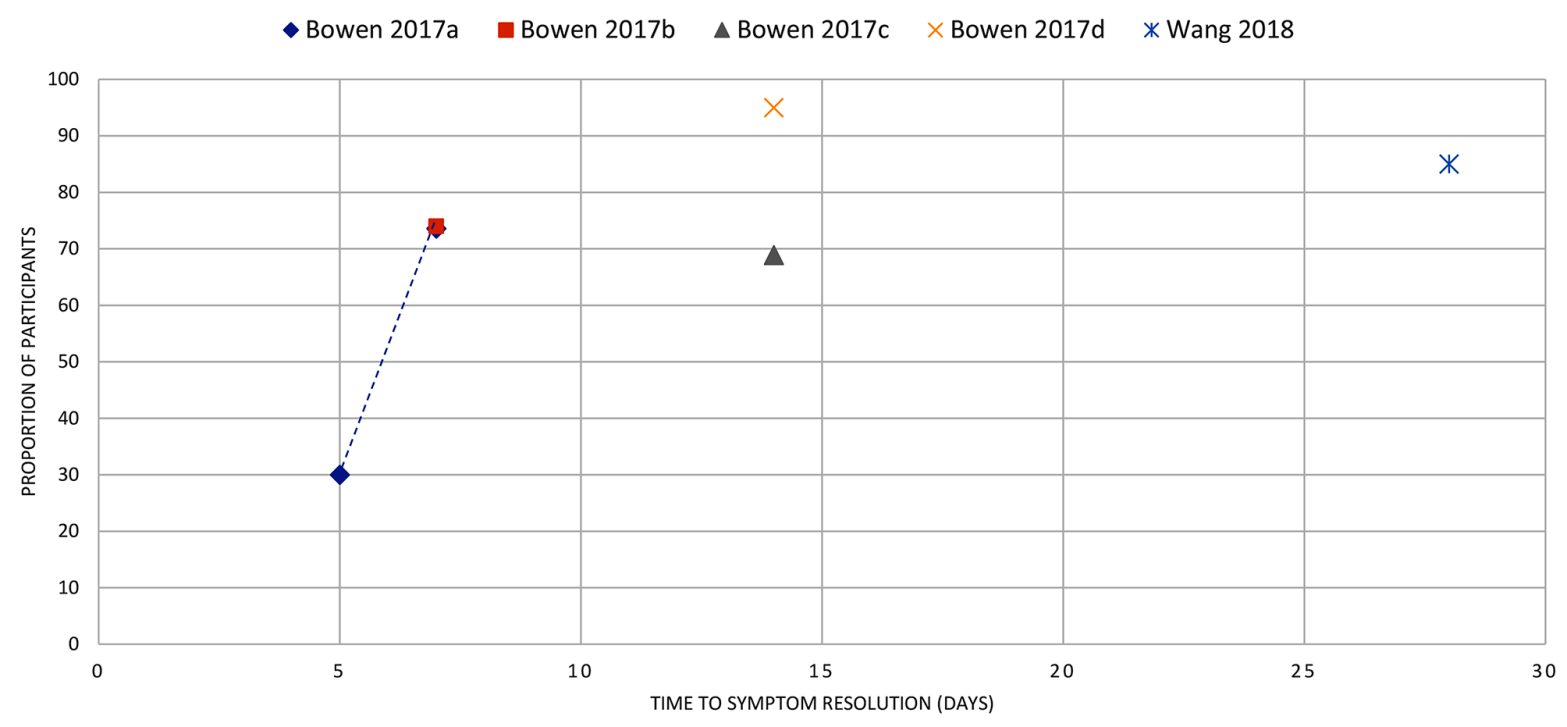
UNCOMPLICATED SKIN ABSCESS: the proportion of participants with symptom resolution at various time points after incision and drainage. Bowen 2017a, b, c, d represents reported data from individual studies narratively presented in the review. Wang 2018: Data reported as treatment failure, which was used to calculate the proportion of participants with symptom resolution (8 trials, 1121 participants)

### ARIs

Common cold (*n* = 11): Seven reviews of placebo-controlled RCTs reported mean duration of the common cold, with a range between 3.2 and 10.3 days. Thompson 2013 [13], the only review with a natural history focus, reported a range of 7 and 15 days (Fig. 3). Five reviews reported data on the proportion of participants with symptom resolution (Fig. 4).

Cough (*n* = 9): The mean duration of cough was reported by six reviews, ranging from 8.9 to 28 days (Fig. 3). One review reported a median duration of 7 to 11 days [36]. Eight reviews reported the proportion of participants with symptom resolution: two reported that by day 14, up to 73% had symptom resolution. The remainder reported that most participants (> 50%) had symptoms resolved by day 15 (Fig. 5).

Sore throat (*n* = 3): One review [13] reported a mean duration of 2 to 6.7 days. One review, with only one primary study with eligible data, reported a median duration of 2.3 days [37]. Three reviews provided the proportion of participants with symptom resolution (Fig. 6).

Acute otitis media (*n* = 3): Only one review [13] reported mean duration, with a range between 0.5 and 9 days (Fig. 3). Three reviews reported the proportion of participants with symptom resolution, with ≥ 50% experiencing resolution by day 3 in all reviews (Fig. 7).

Otitis media with effusion (*n* = 3): No data on mean duration. Three reviews reported data on the proportion of participants who had symptom resolution at various time points (Fig. 8).

Acute sinusitis (*n* = 4): Two reviews reported mean duration [38, 39], with a range between 7 and 14 days, and one review [40] reported a median duration of 9.5 days (Fig. 3). Three reviews reported symptom resolution at various time points, with > 50% of participants experiencing symptom resolution by about day 9 in all reviews (Fig. 9).

Conjunctivitis (*n* = 1): One review [32] reported that 31% of participants had symptoms resolved by day 5 and 42% by day 10 (Fig. 10).

Bronchiolitis (*n* = 2): Two reviews reported a mean duration estimate of 6.6 days in one and between 2 and 6.7 days in the other (Fig. 3). Two reviews reported the proportion with symptom resolution: 84% by day 7 and 90% by day 21 (Fig. 11).

Croup (*n* =1 ): One review [13] reported a mean duration of 2 to 3 days (Fig. 3) and that 50% of participants had symptom resolution by day 1 and 80% by day 2.

Laryngitis (*n* = 1): One review [34] reported the proportion of participants with symptom resolution at day 5, 8, and 28 days (44%, 78%, 76%, respectively), with all data from one study in the review (Fig. 12).

Otitis externa (*n* = 2): One review [30] reported that 15% of placebo participants experienced symptom resolution by day 7 and 10% by day 10 [29] (Fig. 13).

Acute exacerbation of COPD (*n* = 1): We extracted outcome data from five eligible primary studies in one review [35]. Two of these primary studies reported mean duration (of 12.8 days [41] and 13.5 days [42]). One study [43] in the review reported that 26% of participants had exacerbation symptoms resolved by day 5; another [44] reported that by day 10 and day 20, 60% and 67% of participants had symptom resolution (Fig. 14).

Rhinitis (*n* = 1): We extracted data from four eligible primary studies in one review [33]. In one study [45], 17% experienced symptom improvement by week 2; in another study, 51% by week 4 [46]. In another two studies, by week 6, 39% and 49% of placebo participants experienced symptom resolution [47, 48] (Fig. 15).

### Skin and soft tissue infections

Abscesses (*n* = 2): Two reviews reported the resolution of uncomplicated skin abscesses after incision and drainage. One review [23] reported resolution in 85% of participants by day 28. The other review [22] reported the outcome separately for each of the four eligible primary studies, with resolution in 74% of participants by day 7 in two studies and in 69% and 95% by days 10 and 14 in the other two (Fig. 16).

Impetigo (*n* = 1): One review estimated 7 days as the mean duration of impetigo and reported that 13–74% of placebo participants were better by day 7 [26].

### Urinary tract infection (*n* = 1)

One review [15] reported that 42% of participants experienced symptom resolution by day 9. However, most review data were obtained from one of the included trials.

## Discussion

We identified 40 systematic reviews that contained 45 natural history conditions. Nearly all (90%) of the reviews were of ARIs (most for common cold, cough, sore throat, or acute otitis media), with only 4 reporting other infections (three Skin and Soft Tissue (SSTIs), one UTIs). Most existing evidence is contained within treatment effectiveness of systematic reviews of placebo-controlled RCTs and needed to be extracted from the reported placebo group results. Only 6 (15%) reviews aimed to synthesise natural history information. We found no reviews or primary studies with natural history data for some conditions eligible for this review (cellulitis, ecthyma, carbuncles, erysipelas).

A strength of this review is its pragmatic yet comprehensive hierarchical searching approach, which allowed us to identify the best available evidence. The heterogeneity of reported information limited our review’s synthesis of results. We did not attempt to update the evidence and search for newly published primary studies when an eligible systematic review was included, regardless of its year of publication. This may have resulted in the omission of some studies that could have provided additional data.

The over-representation of ARI reviews and the under-representation of UTI and skin and soft tissue infection aligns with the findings of a scoping review of the quantity of randomised placebo-controlled trials of antibiotics, with many more trials conducted in ARIs than other infections [49]. For some conditions, the reported mean duration of illness varied across reviews of the same condition. Likely contributors to the variation include differences in the inclusion criteria of primary studies, the definition of symptom resolution, and the estimated duration of illness before study entry/randomisation. A systematic review of 82 clinical guidelines for acute infections also found some variation in the duration of infections reported, likely due to variations in the body of evidence used in each guideline [20].

Our review provides an up-to-date collation of evidence-based information about the natural history of acute infections commonly seen in primary care. Our findings show that many infections will likely resolve spontaneously, which is important in informing clinical decision-making. While there was variation in the recovery timeframes of some infections across the different reviews, the presented information provides a useful snapshot of the available evidence.

Developers of clinical guidelines for acute conditions are encouraged to include natural history information to facilitate clinicians’ access to it and the ability to incorporate the information into patient discussions as part of antibiotic stewardship strategies such as shared decision making and delayed prescribing. However, this information is missing in about 40% of guidelines [20], and sometimes the information provided in guidelines is not evidence-based [50]. In a recent qualitative study with Australian general practitioners, they identified the value of knowing natural history evidence and using it in consultations, but felt ill-prepared to do so without ready access to it [51]. A study of United Kingdom primary care patients found that natural history information is highly desired, but is the most common unmet need in a consultation [52]. Helping patients understand how long common infections are likely to last and their self-resolving nature may help reduce consultation rates for similar infections. While reporting complication data was not within the review’s scope, complications were uncommon in placebo group participants. As there can be a delicate balance between adverse events from treatment and complications from not treating, advice on the waiting period and what to monitor during a “wait-and-see” period should be informed by evidence when it exists.

This scoping review has highlighted the evidence gaps for the natural history of UTIs and SSTIs. More primary studies are needed, followed by systematic reviews to synthesise natural history evidence. Questions surrounding the necessity of antibiotic use for some SSTI and UTI conditions remain because of entrenched beliefs that antibiotics are always needed for managing these infections [53], hindering the conduct of trials with a placebo or no-antibiotic comparator. Future research, such as meta-analyses, which would also appraise studies’ risk of bias, to formally synthesise natural history evidence would be facilitated if authors of primary studies used outcome measures with similar definitions and time points. The variations noted in this review highlights the need to develop core outcome measures for common infections, such as exists for atopic dermatitis [54].

## Conclusion

Our review has identified what natural history evidence exists for acute infections commonly managed in primary care and often with antibiotics, even though antibiotics may not always be needed. Most existing evidence is for acute respiratory infections, with identified evidence gaps for the natural history of UTIs and numerous SSTIs. Awareness of existing evidence may facilitate its incorporation into clinical practice guidelines and other decision-support tools and, ultimately, its use in antibiotic stewardship strategies such as delayed prescribing and shared decision making. Additionally, researchers are encouraged to conduct research into the natural history of acute infections where little or no evidence exists.

### Electronic supplementary material

Below is the link to the electronic supplementary material.

Supplementary Material 1

## Data Availability

All data generated and analysed during this study are included in this published article [and its Additional Files].

## Abbreviations

RCT: Randomised Controlled Trials
PRISMA-ScR: Preferred Reporting Items for Systematic Reviews and Meta-Analyses Extension for Scoping Reviews
ARI: Acute Respiratory Infection
UTI: Uncomplicated urinary Tract Infection
SSTI: Skin and Soft Tissue Infection
COPD: Chronic Obstructive Pulmonary Diseases
