# Supplementary material for: Mapping the evidence about the natural history of acute infections commonly seen in primary care and managed with antibiotics: a scoping review
Source: BMC Infect Dis. 2024 Jul 23;24:721. doi: 10.1186/s12879-024-09526-3 (PMC11264388; doi:10.1186/s12879-024-09526-3)
Supplement: Supplementary file 1 — Supplementary Material 1 [file 12879_2024_9526_MOESM1_ESM.docx]

**Additional Files**

**Additional Box 1**: Flow chart of the hierarchical search strategy

Interest: Systematic review data

Database: Systematic review of cohort study/RCT reporting Natural History

Primary Studies: randomised controlled trials and prospective cohort studies

Systematic review of antibiotics/other treatment effectiveness

Interest: No treatment or placebo group of eligible primary studies

Interest: No treatment or placebo group

**Additional Box 2:** Search strategies for systematic reviews and primary studies

**Systematic reviews search strategy**

**Systematic reviews Medline:** Search run on the 24^th^ of February 2022

(exp Prognosis/ or Natural history.ti,ab. or Natural course.ti,ab. or Clinical course.ti,ab. or Watchful waiting.ti,ab. or (Wait adj1 see).ti,ab. or Active monitoring.ti,ab. or Prognosis.ti,ab. or Progress.ti,ab. or Progressing.ti,ab. or Progression.ti,ab. or recurrence.ti,ab. Or exp "Cohort Studies"/ OR Cohort.tw. OR "Follow up".tw. OR Observational.tw. OR Longitudinal.tw. OR Prospective.tw. Or ((Symptom or Symptoms or Illness or Disease or Condition OR Spontaneous) adj3 (resolve or resolution or Duration or Length or Time or recurrence or Relapse or Remit or Worsen or Improve or Improves or Improving or prevent OR Recovery)).ti,ab.)

AND (exp Impetigo/ OR Impetigo.tw. OR exp Ecthyma/ or Ecthyma.tw. OR exp Carbuncle / or Carbuncle.tw. OR School sores.tw. OR exp Otitis Media with Effusion/ OR Glue ear.tw. OR otitis media.tw. OR exp Sinusitis/ OR Sinusitis.tw. OR Sinusitides.tw. OR Sinus Infections.tw. OR Sinus Infection.tw. OR Inflamed Sinus.tw. OR Inflamed Sinuses.tw. OR Inflammed Sinus.tw. OR Inflamed Sinuses.tw. OR Sinus Inflammation.tw. OR exp Urinary Tract Infections/ OR Urinary Tract Infections.tw. OR Urinary Tract Infection.tw. OR UTI.tw. OR Cystitis.tw. OR exp Respiratory Tract Infections/ OR Respiratory Tract Infections.tw. OR Cough.tw. OR Respiratory Tract Infection.tw. OR Respiratory Tract disease.tw. OR Respiratory Tract diseases.tw. OR Nasopharyngitis.tw. OR Rhinitis.tw. OR Sinusitis.tw. OR Pharyngitis.tw. OR Laryngitis.tw. OR bronchitis.tw. OR bronchiolitis.tw. OR Pleurisy.tw. OR Cough.tw. OR ((exp COPD/ OR "chronic obstructive pulmonary disease".tw. AND (exp "Symptom Flare Up"/ OR "acute exacerbation".tw.)) OR Sneezing.tw. OR Pneumonia.tw. OR Respiratory Sounds.tw. OR Earache.tw. OR Influenza.tw. OR Common Cold.tw. OR Conjunctivitis.tw. OR Respiratory infection.tw. OR Respiratory infections.tw. OR Respiratory inflammation.tw. OR Respiratory tract inflammation.tw. OR urti.tw. OR lrti.tw. OR Lower Urinary Tract Infections.tw OR ari.tw. OR nasopharyngitis.tw. OR rhinopharyngitis.tw. OR nasosinusitis.tw. OR rhinosinusitis.tw. OR rhinitis.tw. OR rhinorrhoea.tw. OR rhinorrhea.tw. OR pharyngitis.tw. OR sore throat.tw. OR Tonsillitis.tw. OR laryngitis.tw. OR croup.tw. OR pseudocroup.tw. OR tracheobronchitis.tw. OR laryngotracheobronchitis.tw. OR bronchitis.tw. OR bronchiolitis.tw. OR pneumonia.tw. OR pleuropneumonia.tw. OR bronchopneumonia.tw. OR pleurisy.tw. OR Wheeze.tw. OR Wheezing.tw. OR aom.tw. OR ome.tw. OR earache.tw. OR 'otitis externa'.tw/exp OR Otitis externa.tw. OR influenza.tw. OR flu.tw. OR common colds.tw. OR Conjunctivitis.tw. OR Erysipelas.tw. OR Erysipelas.tw. OR Cellulitis.tw. OR Impetigo.tw. OR Soft Tissue Infections.tw. OR Soft Tissue Infection.tw. OR Skin infections.tw. OR Skin infection.tw.)

AND (((Search OR Searched) AND (PubMed OR MEDLINE)).ti,ab. OR (Systematic adj1 Review).ti,ab. OR ((Systematically OR Reviewed) AND (literature)).ti,ab. or Cochrane database of systematic reviews.jn. OR (Meta analysis OR Systematic review).pt.)

**Randomised controlled trial.**

(exp Prognosis/ OR Prognosis.tw. OR “Natural history”.tw. OR “Natural course”.tw. OR “Clinical course”.tw. OR “Watchful waiting”.tw. OR (Wait ADJ1 see ).tw. OR self-remit*.tw. OR self-limit*.tw. OR “Active monitoring”.tw. OR Progress.tw. OR Progressing.tw. OR Progression.tw. OR recurrence.tw. OR “Ongoing symptoms”.tw. OR ((Infection* OR Illness OR Disease OR Condition OR spontaneous* ) ADJ3 (resolv* OR resolution OR Duration OR Length OR Time OR recurrence OR Relapse OR Remit OR Worsen* OR Improv* OR prevent OR Recover* )).tw.)

AND (exp Erysipelas/ OR exp Cellulitis/ OR exp Ecthyma/ OR exp Carbuncle/ OR exp Croup/ OR Carbuncle.tw. OR Carbuncles.tw. OR Cellulitis.tw. OR Croup.tw. OR Pseudocroup.tw. OR Erysipelas.tw. OR Erysipelas.tw. OR Ecthyma.tw. OR Erysipelas.tw. OR “Acute bacterial skin infections”.tw. OR “Acute bacterial skin infection”.tw. OR “Skin structure infections”.tw. OR “Skin structure infection”.tw. OR ABSSSI.tw. OR “Soft Tissue Infections”.tw. OR “Soft Tissue Infection”.tw. OR “Ulcerative pyoderma”.tw.)

AND (“randomized controlled trial”.pt. OR “controlled clinical trial”.pt. OR randomized.tw. OR randomised.tw. OR placebo.tw. OR randomly.tw. OR trial.tw. OR groups.tw.)

**Cohort studies**

(exp Prognosis/ OR Prognosis.tw. OR “Natural history”.tw. OR “Natural course”.tw. OR “Clinical course”.tw. OR “Watchful waiting”.tw. OR (Wait ADJ1 see ).tw. OR self-remit*.tw. OR self-limit*.tw. OR “Active monitoring”.tw. OR Progress.tw. OR Progressing.tw. OR Progression.tw. OR recurrence.tw. OR “Ongoing symptoms”.tw. OR ((Infection* OR Illness OR Disease OR Condition OR spontaneous* ) ADJ3 (resolv* OR resolution OR Duration OR Length OR Time OR recurrence OR Relapse OR Remit OR Worsen* OR Improv* OR prevent OR Recover* )).tw.)

AND (exp Erysipelas/ OR exp Cellulitis/ OR exp Ecthyma/ OR exp Carbuncle/ OR exp Croup/ OR Carbuncle.tw. OR Carbuncles.tw. OR Cellulitis.tw. OR Croup.tw. OR Pseudocroup.tw. OR Erysipelas.tw. OR Erysipelas.tw. OR Ecthyma.tw. OR Erysipelas.tw. OR “Acute bacterial skin infections”.tw. OR “Acute bacterial skin infection”.tw. OR “Skin structure infections”.tw. OR “Skin structure infection”.tw. OR ABSSSI.tw. OR “Soft Tissue Infections”.tw. OR “Soft Tissue Infection”.tw. OR “Ulcerative pyoderma”.tw.)

AND (follow-up.tw. OR prognosis.tw. OR “Epidemiology”.fs. OR prognosis/ OR diagnosed.tw. OR cohort.tw. OR ((History OR Variable$ ) AND (Decision$ OR Identif$ OR Prognos$ )).tw. OR ((Prognostic ) AND (History OR Variable$ )).tw. OR (first ADJ2 episode ).tw. OR incidence/ OR course.tw.)

**Appendix 1: Search strategy for Ovid MEDLINE database**

**Primary studies search strategy**

| **Primary Study Search: Randomised controlled trails**  (exp Prognosis/ OR Prognosis.tw. OR “Natural history”.tw. OR “Natural course”.tw. OR “Clinical course”.tw. OR “Watchful waiting”.tw. OR (Wait ADJ1 see ).tw. OR self-remit*.tw. OR self-limit*.tw. OR “Active monitoring”.tw. OR Progress.tw. OR Progressing.tw. OR Progression.tw. OR recurrence.tw. OR “Ongoing symptoms”.tw. OR ((Infection* OR Illness OR Disease OR Condition OR spontaneous* ) ADJ3 (resolv* OR resolution OR Duration OR Length OR Time OR recurrence OR Relapse OR Remit OR Worsen* OR Improv* OR prevent OR Recover* )).tw.)  AND  (exp Erysipelas/ OR exp Cellulitis/ OR exp Ecthyma/ OR exp Carbuncle/ OR exp Croup/ OR Carbuncle.tw. OR Carbuncles.tw. OR Cellulitis.tw. OR Croup.tw. OR Pseudocroup.tw. OR Erysipelas.tw. OR Erysipelas.tw. OR Ecthyma.tw. OR Erysipelas.tw. OR “Acute bacterial skin infections”.tw. OR “Acute bacterial skin infection”.tw. OR “Skin structure infections”.tw. OR “Skin structure infection”.tw. OR ABSSSI.tw. OR “Soft Tissue Infections”.tw. OR “Soft Tissue Infection”.tw. OR “Ulcerative pyoderma”.tw.)  AND  (“randomized controlled trial”.pt. OR “controlled clinical trial”.pt. OR randomized.tw. OR randomised.tw. OR placebo.tw. OR randomly.tw. OR trial.tw. OR groups.tw.) | **Cohort studies**  (exp Prognosis/ OR Prognosis.tw. OR “Natural history”.tw. OR “Natural course”.tw. OR “Clinical course”.tw. OR “Watchful waiting”.tw. OR (Wait ADJ1 see ).tw. OR self-remit*.tw. OR self-limit*.tw. OR “Active monitoring”.tw. OR Progress.tw. OR Progressing.tw. OR Progression.tw. OR recurrence.tw. OR “Ongoing symptoms”.tw. OR ((Infection* OR Illness OR Disease OR Condition OR spontaneous* ) ADJ3 (resolv* OR resolution OR Duration OR Length OR Time OR recurrence OR Relapse OR Remit OR Worsen* OR Improv* OR prevent OR Recover* )).tw.)  AND  (exp Erysipelas/ OR exp Cellulitis/ OR exp Ecthyma/ OR exp Carbuncle/ OR exp Croup/ OR Carbuncle.tw. OR Carbuncles.tw. OR Cellulitis.tw. OR Croup.tw. OR Pseudocroup.tw. OR Erysipelas.tw. OR Erysipelas.tw. OR Ecthyma.tw. OR Erysipelas.tw. OR “Acute bacterial skin infections”.tw. OR “Acute bacterial skin infection”.tw. OR “Skin structure infections”.tw. OR “Skin structure infection”.tw. OR ABSSSI.tw. OR “Soft Tissue Infections”.tw. OR “Soft Tissue Infection”.tw. OR “Ulcerative pyoderma”.tw.)  AND  (follow-up.tw. OR prognosis.tw. OR “Epidemiology”.fs. OR prognosis/ OR diagnosed.tw. OR cohort.tw. OR ((History OR Variable$ ) AND (Decision$ OR Identif$ OR Prognos$ )).tw. OR ((Prognostic ) AND (History OR Variable$ )).tw. OR (first ADJ2 episode ).tw. OR incidence/ OR course.tw.) |
| --- | --- |

**Supplementary Box 3:** Inclusion and Exclusion criteria for common infections

| System | Included conditions | Excluded conditions |
| --- | --- | --- |
| Respiratory tract infections | Acute otitis media, acute otitis media with effusion, otitis externa, pharyngitis, streptococcal pharyngitis, tonsillitis, common cold, rhinosinusitis, sinusitis, laryngitis, acute bronchitis including subacute or protracted cough, bronchiolitis, nonallergic rhinitis, simple bacterial and viral conjunctivitis, croup, acute exacerbation of Chronic Obstructive Pulmonary Disease (COPD). | Chronic cough, COPD, chronic otitis media, chronic sinusitis, chronic bronchitis), allergic rhinitis, lower respiratory tract infections (e.g., pneumonia), meningitis, Covid-19; avian flu; severe acute respiratory syndrome; influenza; influenza-like illness; and chronic or recurrent respiratory infections. |
| Urinary Tract infection | Uncomplicated lower urinary tract infections | Chronic or recurrent urinary tract infections, asymptomatic bacteriuria, interstitial cystitis, UTI in children, |
| Skin and Soft tissue infection | Cellulitis, erysipelas, impetigo, boils, carbuncles, ecthyma, pityriasis rosea | Severe infections (e.g., necrotising tissue infections), psoriasis, leishmaniasis, genital warts, herpes, atopic dermatitis, eczema, Erythema, scabies, atopic dermatitis, chronic or recurrent skin and soft tissue infections. |

**Additional table 1:** List of included reviews

| **Respiratory tract infections** |
| --- |
| 1. Becker, L. A., Hom, J., Villasis-Keever, M., & van der Wouden, J. C. (2015). Beta2-agonists for acute cough or a clinical diagnosis of acute bronchitis. *Cochrane Database of Systematic Reviews*, *2015*(9). https://doi.org/10.1002/14651858.CD001726.pub5 |
| 1. Bergmann, M., Haasenritter, J., Beidatsch, D., Schwarm, S., Hörner, K., Bösner, S., Grevenrath, P., Schmidt, L., Viniol, A., Donner-Banzhoff, N., & Becker, A. (2021). Prevalence, aetiologies and prognosis of the symptom cough in primary care: a systematic review and meta-analysis. BMC Family Practice, 22(1), 151. <https://doi.org/10.1186/s12875-021-01501-0> |
| 1. de Cassan, S., Thompson, M. J., Perera, R., Glasziou, P. P., Del Mar, C. B., Heneghan, C. J., & Hayward, G. (2020). Corticosteroids as standalone or add‐on treatment for sore throat. *Cochrane Database of Systematic Reviews*(5). https://doi.org/10.1002/14651858.CD008268.pub3 |
| 1. De Sutter, A. I. M., Eriksson, L., & van Driel, M. L. (2022). Oral antihistamine‐decongestant‐analgesic combinations for the common cold. Cochrane Database of Systematic Reviews(1). <https://doi.org/10.1002/14651858.CD004976.pub4> |
| 1. De Sutter, A. I. M., Saraswat, A., & van Driel, M. L. (2015). Antihistamines for the common cold. Cochrane Database of Systematic Reviews(11). <https://doi.org/10.1002/14651858.CD009345.pub2> |
| 1. Deckx, L., De Sutter, A. I. M., Guo, L., Mir, N. A., & van Driel, M. L. (2016). Nasal decongestants in monotherapy for the common cold. *Cochrane Database of Systematic Reviews*(10). https://doi.org/10.1002/14651858.CD009612.pub2 |
| 1. Douglas, R. M., Chalker, E. B., & Treacy, B. (2000). Vitamin C for preventing and treating the common cold. *Cochrane Database of Systematic Reviews*(2), CD000980. |
| 1. Ebell, M. H., Lundgren, J., & Youngpairoj, S. (2013). How long does a cough last? Comparing patients' expectations with data from a systematic review of the literature. *Annals of family medicine*, *11*(1), 5-13. https://doi.org/10.1370/afm.1430 |
| 1. Fahey, T., Stocks, N., & Thomas, T. (1998). Quantitative systematic review of randomised controlled trials comparing antibiotic with placebo for acute cough in adults. *British Medical Journal*, *316*(7135), 906-910. https://doi.org/10.1136/bmj.316.7135.906 |
| 1. Gadomski, A. M., & Scribani, M. B. (2014). Bronchodilators for bronchiolitis. *Cochrane Database of Systematic Reviews*(6). https://doi.org/10.1002/14651858.CD001266.pub4 |
| 1. Griffin, G., & Flynn, C. A. (2011). Antihistamines and/or decongestants for otitis media with effusion (OME) in children. *Cochrane Database of Systematic Reviews*(9). https://doi.org/10.1002/14651858.CD003423.pub3 |
| 1. Hay, A. D., & Wilson, A. D. (2002). The natural history of acute cough in children aged 0 to 4 years in primary care: A systematic review. *British Journal of General Practice*, *52*(478), 401-409. |
| 1. Hayward, G., Thompson, M. J., Perera, R., Del Mar, C. B., Glasziou, P. P., & Heneghan, C. J. (2015). Corticosteroids for the common cold. *Cochrane Database of Systematic Reviews*(10). https://doi.org/10.1002/14651858.CD008116.pub3 |
| 1. Karsch‐Völk, M., Barrett, B., Kiefer, D., Bauer, R., Ardjomand‐Woelkart, K., & Linde, K. (2014). Echinacea for preventing and treating the common cold. *Cochrane Database of Systematic Reviews*(2). https://doi.org/10.1002/14651858.CD000530.pub3 |
| 1. Kaushik, V., Malik, T., & Saeed, S. R. (2010). Interventions for acute otitis externa. *Cochrane Database of Systematic Reviews*(1). https://doi.org/10.1002/14651858.CD004740.pub2 |
| 1. Kenealy, T., & Arroll, B. (2013). Antibiotics for the common cold and acute purulent rhinitis. *Cochrane Database of Systematic Reviews*(6). https://doi.org/10.1002/14651858.CD000247.pub3 |
| 1. Kim, S. Y., Chang, Y. J., Cho, H. M., Hwang, Y. W., & Moon, Y. S. (2015). Non-steroidal anti-inflammatory drugs for the common cold. *Cochrane Database of Systematic Reviews*(9), CD006362. https://doi.org/https://dx.doi.org/10.1002/14651858.CD006362.pub4 |
| 1. Lemiengre MB, van Driel ML, Merenstein D, Liira H, Mäkelä M, De Sutter AIM. Antibiotics for acute rhinosinusitis in adults. Cochrane Database of Systematic Reviews 2018, Issue 9. Art. No.: CD006089. DOI: 10.1002/14651858.CD006089 |
| 1. Lissiman, E., Bhasale, A. L., & Cohen, M. (2014). Garlic for the common cold. *Cochrane Database of Systematic Reviews*(11). https://doi.org/10.1002/14651858.CD006206.pub4 |
| 1. Reveiz, L., & Cardona, A. F. (2015). Antibiotics for acute laryngitis in adults. *Cochrane Database of Systematic Reviews*(5). https://doi.org/10.1002/14651858.CD004783.pub5 |
| 1. Rosenfeld, R. M., & Kay, D. (2003). Natural history of untreated otitis media. *Laryngoscope*, *113*(10), 1645-1657. https://doi.org/10.1097/00005537-200310000-00004 |
| 1. Rosenfeld RM, Singer M, Wasserman JM, Stinnett SS. Systematic review of topical antimicrobial therapy for acute otitis externa. Otolaryngol Head Neck Surg. 2006 Apr;134(4 Suppl):S24-48. doi: 10.1016/j.otohns.2006.02.013 |
| 1. Science, M., Johnstone, J., Roth, D. E., Guyatt, G., & Loeb, M. (2012). Zinc for the treatment of the common cold: A systematic review and meta-analysis of randomized controlled trials. *CMAJ*, *184*(10), E551-E561. https://doi.org/10.1503/cmaj.111990 |
| 1. Segboer, C., Gevorgyan, A., Avdeeva, K., Chusakul, S., Kanjanaumporn, J., Aeumjaturapat, S., Reeskamp, L. F., Snidvongs, K., & Fokkens, W. (2019). Intranasal corticosteroids for non‐allergic rhinitis. *Cochrane Database of Systematic Reviews*(11). https://doi.org/10.1002/14651858.CD010592.pub2 |
| 1. Shaikh N, Wald ER. Decongestants, antihistamines and nasal irrigation for acute sinusitis in children. Cochrane Database of Systematic Reviews 2014, Issue 10. Art. No.: CD007909. DOI: 10.1002/14651858.CD007909.pub4. |
| 1. Sheikh, A., Hurwitz, B., van Schayck, C. P., McLean, S., & Nurmatov, U. (2012). Antibiotics versus placebo for acute bacterial conjunctivitis. *Cochrane Database of Systematic Reviews*(9). https://doi.org/10.1002/14651858.CD001211.pub3 |
| 1. Smith, S. M., Fahey, T., Smucny, J., & Becker, L. A. (2017). Antibiotics for acute bronchitis. *Cochrane Database of Systematic Reviews*(6). https://doi.org/10.1002/14651858.CD000245.pub4 |
| 1. Speich, B., Thomer, A., Aghlmandi, S., Ewald, H., Zeller, A., & Hemkens, L. G. (2018). Treatments for subacute cough in primary care: systematic review and meta-analyses of randomised clinical trials. *British Journal of General Practice*, *68*(675), e694-e702. |
| 1. Spinks, A., Glasziou, P. P., & Del Mar, C. B. (2021). Antibiotics for treatment of sore throat in children and adults. *Cochrane Database of Systematic Reviews*(12). https://doi.org/10.1002/14651858.CD000023.pub5 |
| 1. Thompson, M., Vodicka, T. A., Blair, P. S., Buckley, D. I., Heneghan, C., & Hay, A. D. (2013). Duration of symptoms of respiratory tract infections in children: Systematic review. *BMJ (Online)*, *347*. https://doi.org/10.1136/bmj.f7027 |
| 1. Venekamp, R. P., Burton, M. J., van Dongen, T. M., van der Heijden, G. J., van Zon, A., & Schilder, A. G. (2016). Antibiotics for otitis media with effusion in children. *Cochrane Database of Systematic Reviews*(6), CD009163. https://doi.org/https://dx.doi.org/10.1002/14651858.CD009163.pub3 |
| 1. Venekamp, R. P., Sanders, S. L., Glasziou, P. P., Del Mar, C. B., & Rovers, M. M. (2015). Antibiotics for acute otitis media in children. *Cochrane Database of Systematic Reviews*(6). https://doi.org/10.1002/14651858.CD000219.pub4 |
| 1. Venekamp, R. P., Thompson, M. J., Hayward, G., Heneghan, C. J., Del Mar, C. B., Perera, R., Glasziou, P. P., & Rovers, M. M. (2014). Systemic corticosteroids for acute sinusitis. *Cochrane Database of Systematic Reviews*(3). https://doi.org/10.1002/14651858.CD008115.pub3 |
| 1. Vollenweider, D. J., Frei, A., Steurer‐Stey, C. A., Garcia‐Aymerich, J., & Puhan, M. A. (2018). Antibiotics for exacerbations of chronic obstructive pulmonary disease. *Cochrane Database of Systematic Reviews*(10). https://doi.org/10.1002/14651858.CD010257.pub2 |
| 1. Wagner, L., Cramer, H., Klose, P., Lauche, R., Gass, F., Dobos, G., & Langhorst, J. (2015). Herbal Medicine for Cough: a Systematic Review and Meta-Analysis. *Forschende Komplementarmedizin (2006)*, *22*(6), 359-368. https://doi.org/https://dx.doi.org/10.1159/000442111 |
| 1. Zalmanovici Trestioreanu, A., & Yaphe, J. (2013). Intranasal steroids for acute sinusitis. *Cochrane Database of Systematic Reviews*(12). https://doi.org/10.1002/14651858.CD005149.pub4 |
| **Urinary Tract infections** |
| 1. Hoffmann, T., Peiris, R., Del Mar, C., Cleo, G., & Glasziou, P. (2020). Natural history of uncomplicated urinary tract infection without antibiotics: A systematic review. *British Journal of General Practice*, *70*(699), E714-E722. https://doi.org/10.3399/bjgp20X712781 |
| **Skin and soft tissue infections** |
| 1. Bowen, A. C., Carapetis, J. R., Currie, B. J., Fowler, V., Chambers, H. F., & Tong, S. Y. C. (2017). Sulfamethoxazole-Trimethoprim (Cotrimoxazole) for Skin and Soft Tissue Infections including Impetigo, Cellulitis, and Abscess. *Open Forum Infectious Diseases*, *4*(4). https://doi.org/10.1093/ofid/ofx232 |
| 1. Wang W, Chen W, Liu Y, et alAntibiotics for uncomplicated skin abscesses: systematic review and network meta-analysisBMJ Open 2018;8:e020991. doi: 10.1136/bmjopen-2017-020991 |
| 1. Hoffmann, T. C., Peiris, R., Glasziou, P., Cleo, G., & Del Mar, C. (2021). Natural history of non-bullous impetigo: A systematic review of time to resolution or improvement without antibiotic treatment. *British Journal of General Practice*, *71*(704), E237-E242. https://doi.org/10.3399/bjgp20X714149 |

**Additional Table 2:** List of excluded reviews

| Study | Reasons for exclusion |
| --- | --- |
| 1. Koning, S., Verhagen, A. P., van Suijlekom-Smit, L. W., Morris, A., Butler, C. C., & van der Wouden, J. C. (2004). Interventions for impetigo. Cochrane database of systematic reviews (Online)(2), CD003261. | Duplicate |
| 1. Rosenfeld, R. M., Singer, M., & Jones, S. (2007). Systematic review of antimicrobial therapy in patients with acute rhinosinusitis. Otolaryngology - Head and Neck Surgery, 137(3 SUPPL.), S32-S45.e39. https://doi.org/10.1016/j.otohns.2007.06.724 | Duplicate |
| 1. Rosenfeld, R. M., Vertrees, A. J. E., Carr, J., Cipoile, R. J., Uden, D. L., Giebink, G. S., & Canafax, D. M. (1994). Clinical efficacy of antimicrobial drugs for acute otitis media: Metaanalysis of 5400 children from thirty-three randomized trials (0022-3476). (Journal of Pediatrics, Issue. | Duplicate |
| 1. Rovers, M. M., Glasziou, P., Appelman, C. L., Burke, P., McCormick, D. P., Damoiseaux, R. A., Gaboury, I., Little, P., & Hoes, A. W. (2006). Antibiotics for acute otitis media: a meta-analysis with individual patient data. Lancet, 368(9545), 1429-1435. | Duplicate |
| 1. Sheikh, A., & Hurwitz, B. (2001). Topical antibiotics for acute bacterial conjunctivitis: a systematic review. British Journal of General Practice, 51(467), 473-477. | Duplicate |
| 1. Venekamp, R. P., Sanders, S. L., Glasziou, P. P., Del Mar, C. B., & Rovers, M. M. (2015). Antibiotics for acute otitis media in children. Cochrane Database of Systematic Reviews(6). https://doi.org/10.1002/14651858.CD000219.pub4 | Duplicate |
| 1. Vouloumanou, E., Karageorgopoulos, D., Kazantzi, M., Kapaskelis, A., & Falagas, M. (2009). Antibiotics versus placebo or watchful waiting for acute otitis media: A meta-analysis of randomised controlled trials. Clinical Microbiology and Infection, 15, S563-S564. https://doi.org/10.1111/j.1469-0691.2009.02858.x | Duplicate |
| 1. Flynn, C. A., Griffin, G. H., & Schultz, J. K. (2004). Decongestants and antihistamines for acute otitis media in children. Cochrane database of systematic reviews (Online)(3), CD001727. | Duplicate |
| 1. Glasziou, P. P., Del Mar, C. B., Sanders, S. L., & Hayem, M. (2004). Antibiotics for acute otitis media in children. Cochrane database of systematic reviews (Online)(1), CD000219. | Duplicate |
| 1. Hayward, G., Thompson, M., Heneghan, C., Perera, R., Del Mar, C., & Glasziou, P. (2009). Corticosteroids for pain relief in sore throat: Systematic review and meta-analysis. BMJ (Online), 339(7719), 488-490. https://doi.org/10.1136/bmj.b2976 | Duplicate |
| 1. Hayward, G., Thompson, M. J., Perera, R., Del Mar, C. B., Glasziou, P. P., & Heneghan, C. J. (2012). Corticosteroids for the common cold. Cochrane database of systematic reviews (Online), 8, CD008116. | Duplicate |
| 1. Smucny, J., Fahey, T., Becker, L., & Glazier, R. (2004). Antibiotics for acute bronchitis. Cochrane database of systematic reviews (Online)(4), CD000245. | Duplicate |
| 1. Smucny, J. J., Becker, L. A., Glazier, R. H., & McIsaac, W. (1998). Are antibiotics effective treatment for acute bronchitis? A meta-analysis. Journal of Family Practice, 47(6), 453-460. | Duplicate |
| 1. Smucny, J. J., Flynn, C. A., Becker, L. A., & Glazier, R. H. (2001). Are β2-agonists effective treatment for acute bronchitis or acute cough in patients without underlying pulmonary disease? A systematic review. Journal of Family Practice, 50(11), 945-951. | Duplicate |
| 1. Speich, B., Thomer, A., Aghlmandi, S., Ewald, H., Zeller, A., & Hemkens, L. G. (2018). Treatments for subacute cough in primary care: systematic review and meta-analyses of randomised clinical trials. British Journal of General Practice, 68(675), e694-e702. https://doi.org/https://dx.doi.org/10.3399/bjgp18X698885 | Duplicate |
| 1. Spinks, A., Glasziou, P. P., & Del Mar, C. B. (2013). Antibiotics for sore throat. Cochrane Database of Systematic Reviews, 2013(11). https://doi.org/10.1002/14651858.CD000023.pub4 | Duplicate |
| 1. Spurling, G. K., Doust, J., Del Mar, C. B., & Eriksson, L. (2011). Antibiotics for bronchiolitis in children. Cochrane database of systematic reviews (Online)(6), CD005189. | Duplicate |
| 1. van Zon, A., van der Heijden, G. J., van Dongen, T. M., Burton, M. J., & Schilder, A. G. (2012). Antibiotics for otitis media with effusion in children. Cochrane Database of Systematic Reviews(9), CD009163. https://doi.org/https://dx.doi.org/10.1002/14651858.CD009163.pub2 | Duplicate |
| 1. Vollenweider, D. J., Frei, A., Steurer‐Stey, C. A., Garcia‐Aymerich, J., & Puhan, M. A. (2018). Antibiotics for exacerbations of chronic obstructive pulmonary disease. Cochrane Database of Systematic Reviews(10). https://doi.org/10.1002/14651858.CD010257.pub2 | Duplicate |
| 1. Vorwerk, C., & Coats, T. J. (2008). Use of helium-oxygen mixtures in the treatment of croup: A systematic review. Emergency Medicine Journal, 25(9), 547-550. https://doi.org/10.1136/emj.2007.052878 | Duplicate |
| 1. Young, J., De Sutter, A., Merenstein, D., van Essen, G. A., Kaiser, L., Varonen, H., Williamson, I., & Bucher, H. C. (2008). Antibiotics for adults with clinically diagnosed acute rhinosinusitis: a meta-analysis of individual patient data. Lancet, 371(9616), 908-914. | Duplicate |
| 1. Zalmanovici, A., & Yaphe, J. (2007). Steroids for acute sinusitis. Cochrane Database of Systematic Reviews(2). https://doi.org/10.1002/14651858.CD005149.pub2 | Duplicate |
| 1. Kaushik, V., Malik, T., & Saeed, S. R. (2010). Interventions for acute otitis externa. Cochrane Database of Systematic Reviews(1). https://doi.org/10.1002/14651858.CD004740.pub2 | Duplicate |
| 1. Wang, M. X., Win, S. S., & Pang, J. (2020). Zinc supplementation reduces common cold duration among healthy adults: A systematic review of randomized controlled trials with micronutrients supplementation. American Journal of Tropical Medicine and Hygiene, 103(1), 86-99. https://doi.org/10.4269/ajtmh.19-0718 | Duplicate |
| 1. Abba, K., Gulani, A., & Sachdev, H. S. (2010). Zinc supplements for preventing otitis media. Cochrane database of systematic reviews (Online), 2(2), CD006639. https://doi.org/10.1002/14651858.CD006639.pub2 | Ineligible study design |
| 1. Abioye, A. I., Bromage, S., & Fawzi, W. (2021). Effect of micronutrient supplements on influenza and other respiratory tract infections among adults: a systematic review and meta-analysis. BMJ Global Health, 6(1), 01. https://doi.org/https://dx.doi.org/10.1136/bmjgh-2020-003176 | Ineligible study design |
| 1. Anderson-James, S., Marchant, J. M., Acworth, J. P., Turner, C., & Chang, A. B. (2013). Inhaled corticosteroids for subacute cough in children. Cochrane Database of Systematic Reviews, 2013(2). https://doi.org/10.1002/14651858.CD008888.pub2 | Ineligible study design |
| 1. Ariathianto, Y. (2011). Asymptomatic bacteriuria - prevalence in the elderly population. Australian Family Physician, 40(10), 805-809. | Ineligible study design |
| 1. Ausejo, M., Saenz, A., Pham, B., Kellner, J. D., Johnson, D. W., Moher, D., & Klassen, T. P. (1999). The effectiveness of glucocorticoids in treating croup: meta-analysis. BMJ, 319(7210), 595-600. | Ineligible study design |
| 1. Baars, E. W., van der Werf-Kok, E., Willcox, M., Huber, R., Belt-Van Zoen, E., & Hu, X. Y. (2021). Can CAM treatment strategies control symptoms of respiratory tract infections (RTIs) and reduce antibiotic use? A SR of SRs. European Journal of Integrative Medicine, 48. https://doi.org/10.1016/j.eujim.2021.101889 | Ineligible study design |
| 1. Barnett, M. (2012). Do intranasal steroids improve symptoms of acute sinusitis? American Family Physician, 86(7), 680-682. | Ineligible study design |
| 1. Bird, J. H., Biggs, T. C., & King, E. V. (2014). Controversies in the management of acute tonsillitis: An evidence-based review. Clinical Otolaryngology, 39(6), 368-374. https://doi.org/10.1111/coa.12299 | Ineligible study design |
| 1. Blom, D., Ermers, M., Bont, L., Van Aalderen, W. M. C., & Van Woensel, J. B. M. (2007). Inhaled corticosteroids during acute bronchiolitis in the prevention of post-bronchiolitic wheezing [Review]. Cochrane Database of Systematic Reviews(1). https://doi.org/10.1002/14651858.CD004881.pub2 | Ineligible study design |
| 1. Bolser, D. C. (2006). Cough suppressant and pharmacologic protussive therapy: ACCP evidence-based clinical practice guidelines. Chest, 129(1 Suppl), 238S-249S. https://doi.org/https://dx.doi.org/10.1378/chest.129.1_suppl.238S | Ineligible study design |
| 1. Bourke, T., & Shields, M. (2011). Bronchiolitis. BMJ clinical evidence, 2011. | Ineligible study design |
| 1. Burkin, A. V., Svistushkin, V. M., Nikiforova, G. N., & Dukhanin, A. S. (2019). [Glucosaminylmuramyl dipeptide in treatment of respiratory tract diseases]. Vestnik Otorinolaringologii, 84(6), 118-131. https://doi.org/https://dx.doi.org/10.17116/otorino201984061118 | Ineligible study design |
| 1. Casale, M., Vella, P., Moffa, A., Grimaldi, V., Sabatino, L., Rinaldi, V., Lopez, M. A., Baptista, P., & Salvinelli, F. (2017). The efficacy of topical hyaluronan in rhinosinusitis: A systematic review. Journal of Biological Regulators and Homeostatic Agents, 31(4), 71-80. | Ineligible study design |
| 1. Chandran, R. (2001). Should we prescribe antibiotics for acute bronchitis? American Family Physician, 64(1), 135-138. | Ineligible study design |
| 1. Cohen, J. F., Pauchard, J. Y., Hjelm, N., Cohen, R., & Chalumeau, M. (2020). Efficacy and safety of rapid tests to guide antibiotic prescriptions for sore throat. Cochrane Database of Systematic Reviews(6). https://doi.org/10.1002/14651858.CD012431.pub2 | Ineligible study design |
| 1. Croessmann, M., & Rose, M. A. (2016). Holistic therapeutic options for the common cold. Pneumologe, 13(4), 262-273. https://doi.org/10.1007/s10405-016-0052-3 | Ineligible study design |
| 1. Damoiseaux, R. A., van Balen, F. A., Hoes, A. W., & de Melker, R. A. (1998). Antibiotic treatment of acute otitis media in children under two years of age: evidence based? British Journal of General Practice, 48(437), 1861-1864. | Ineligible study design |
| 1. Deot, N., Barr, J., Mankowski, N., Brunner, J., & McCoul, E. D. (2019). Effect of Intranasal Corticosteroids on Secondary Sinonasal Symptoms: A Systematic Review of Randomized Trials. American Journal of Rhinology & Allergy, 33(5), 601-607. | Ineligible study design |
| 1. Dilger, A. E., Peters, A. T., Wunderink, R. G., Tan, B. K., Kern, R. C., Conley, D. B., Welch, K. C., Holl, J. L., & Smith, S. S. (2019). Procalcitonin as a Biomarker in Rhinosinusitis: A Systematic Review. American Journal of Rhinology and Allergy, 33(2), 103-112. https://doi.org/10.1177/1945892418810293 | Ineligible study design |
| 1. Dineen-Griffin, S., Garcia-Cardenas, V., Williams, K., & Benrimoj, S. I. (2019). Helping patients help themselves: A systematic review of self-management support strategies in primary health care practice. PLoS One, 14(8), e0220116. https://doi.org/10.1371/journal.pone.0220116 | Ineligible study design |
| 1. Diaz-Saez, G., Diaz-Diez, C., Sacristan-Rubio, A., Garcia-Gomez, O., Dominguez-Agüero, M. N., & Ramirez-Lapausa, M. (2018). Review of effectiveness studies of homeopathy for respiratory and ENT complaints [Conference Abstract]. Homeopathy, 107. https://doi.org/10.1055/s-0038-1633319 | Ineligible study design |
| 1. Dudasova, A., Hyyrylainen, A., & Franciosi, L. (2010). A preliminary investigation into the baseline characteristics and treatment effect measures of various study populations of cough patients. American Journal of Respiratory and Critical Care Medicine, 181(1). | Ineligible study design |
| 1. Eaton, C. B. (2001). Should we prescribe antibiotics for acute otitis media? American Family Physician, 64(3), 469-470. | Ineligible study design |
| 1. Edwards, G., Newbould, L., Nesbitt, C., Rogers, M., Morris, R. L., Hay, A. D., Campbell, S. M., & Hayward, G. (2021). Predicting poor outcomes in children aged 1–12 with respiratory tract infections: A systematic review. PLoS ONE, 16(4 April). https://doi.org/10.1371/journal.pone.0249533 | Ineligible study design |
| 1. Engel, M. F., Paling, F. P., Hoepelman, A. I., van der Meer, V., & Oosterheert, J. J. (2012). Evaluating the evidence for the implementation of C-reactive protein measurement in adult patients with suspected lower respiratory tract infection in primary care: a systematic review. Family Practice, 29(4), 383-393. https://doi.org/https://dx.doi.org/10.1093/fampra/cmr119 | Ineligible study design |
| 1. Fixsen, A. (2013). Should homeopathy be considered as part of a treatment strategy for otitis media with effusion in children? Homeopathy: the Journal of the Faculty of Homeopathy, 102(2), 145-150. https://doi.org/https://dx.doi.org/10.1016/j.homp.2013.01.004 | Ineligible study design |
| 1. Flottorp, S., Oxman, A. D., Cooper, J. G., Hjortdahl, P., Sandberg, S., & Vorland, L. H. (2000). Guidelines for diagnosis and treatment of sore throat. Läkartidningen, 97(40), 4437-4442, 4445-4446, 4448. | Ineligible study design |
| 1. Galli, L., Venturini, E., Bassi, A., Gattinara, G. C., Chiappini, E., Defilippi, C., Diociaiuti, A., Esposito, S., Garazzino, S., Giannattasio, A., Krzysztofiak, A., Latorre, S., Lo Vecchio, A., Marchisio, P., Montagnani, C., Nicolini, G., Novelli, A., Rossolini, G. M., Tersigni, C., . . . Neri, I. (2019). Common Community-acquired Bacterial Skin and Soft-tissue Infections in Children: an Intersociety Consensus on Impetigo, Abscess, and Cellulitis Treatment. Clinical Therapeutics, 41(3), 532-551.e517. https://doi.org/10.1016/j.clinthera.2019.01.010 | Ineligible study design |
| 1. Hamerlynck, J. V., Rietveld, R. P., & Hooft, L. (2007). [From the Cochrane Library: Marginally higher chance of cure by antibiotic treatment in acute bacterial conjunctivitis]. Nederlands Tijdschrift voor Geneeskunde, 151(10), 594-596. | Ineligible study design |
| 1. Hanson, L., Vandevusse, L., Jermé, M., Abad, C. L., & Safdar, N. (2016). Probiotics for Treatment and Prevention of Urogenital Infections in Women: A Systematic Review. Journal of Midwifery and Women's Health, 61(3), 339-355. https://doi.org/10.1111/jmwh.12472 | Ineligible study design |
| 1. Hartling, L., Wiebe, N., Russell, K., Patel, H., & Klassen, T. P. (2003). A meta-analysis of randomized controlled trials evaluating the efficacy of epinephrine for the treatment of acute viral bronchiolitis. Archives of Pediatrics & Adolescent Medicine, 157(10), 957-964. | Ineligible study design |
| 1. Heimer, K. A., Hart, A. M., Martin, L. G., & Rubio-Wallace, S. (2009). Examining the evidence for the use of vitamin C in the prophylaxis and treatment of the common cold. Journal of the American Academy of Nurse Practitioners, 21(5), 295-300. https://doi.org/10.1111/j.1745-7599.2009.00409.x | Ineligible study design |
| 1. Hemila, H., & Chalker, E. (2015). The effectiveness of high dose zinc acetate lozenges on various common cold symptoms: a meta-analysis. BMC Family Practice, 16, 24. https://doi.org/https://dx.doi.org/10.1186/s12875-015-0237-6 | Ineligible study design |
| 1. Hemila, H., Petrus, E. J., Fitzgerald, J. T., & Prasad, A. (2016). Zinc acetate lozenges for treating the common cold: an individual patient data meta-analysis. British Journal of Clinical Pharmacology, 82(5), 1393-1398. https://doi.org/https://dx.doi.org/10.1111/bcp.13057 | Ineligible study design |
| 1. Hulisz, D. (2004). Efficacy of zinc against common cold viruses: an overview. Journal of the American Pharmacists Association: JAPhA, 44(5), 594-603. | Ineligible study design |
| 1. Jat, K. R., & Mathew, J. L. (2019). Continuous positive airway pressure (CPAP) for acute bronchiolitis in children. Cochrane Database of Systematic Reviews(1). https://doi.org/10.1002/14651858.CD010473.pub3 | Ineligible study design |
| 1. Jefferis, J., Perera, R., Everitt, H., van Weert, H., Rietveld, R., Glasziou, P., & Rose, P. (2011). Acute infective conjunctivitis in primary care: who needs antibiotics? An individual patient data meta-analysis. British Journal of General Practice, 61(590), e542-548. https://doi.org/https://dx.doi.org/10.3399/bjgp11X593811 | Ineligible study design |
| 1. Jepson, R. G., & Craig, J. C. (2007). A systematic review of the evidence for cranberries and blueberries in UTI prevention. Molecular Nutrition & Food Research, 51(6), 738-745. | Ineligible study design |
| 1. Jiang, H., Liu, W., Li, G., Fan, T., & Mao, B. (2016). Chinese Medicinal Herbs in the Treatment of Upper Airway Cough Syndrome: A Systematic Review of Randomized, Controlled Trials. Alternative therapies in health and medicine, 22(3), 38-51. | Ineligible study design |
| 1. Johnson, D. W. (2014). Croup. BMJ clinical evidence, 2014. | Ineligible study design |
| 1. Kardos, P. (2015). Phytotherapy in acute bronchitis: what is the evidence? Clinical Phytoscience, 1(1). https://doi.org/10.1186/s40816-015-0003-2 | Ineligible study design |
| 1. Ah-See, K. (2011). Sinusitis (acute). BMJ clinical evidence, 2011. | Ineligible study design |
| 1. Ahovuo-Saloranta, A., Rautakorpi, U. M., Borisenko, O. V., Liira, H., Williams, J. W., & Mäkelä, M. (2015). Antibiotics for acute maxillary sinusitis in adults. Cochrane Database of Systematic Reviews, 2015(10), CD000243. https://doi.org/10.1002/14651858.CD000243.pub4 | Ineligible study design |
| 1. Arroll, B. (2011). Common cold. BMJ clinical evidence, 2011. | Ineligible study design |
| 1. Di Pasquale, M., Aliberti, S., Mantero, M., Gramegna, A., & Blasi, F. (2020). Pharmacotherapeutic management of bronchial infections in adults: non-cystic fibrosis bronchiectasis and chronic obstructive pulmonary disease. Expert Opinion on Pharmacotherapy, 21(16), 1975-1990. https://doi.org/10.1080/14656566.2020.1793958 | Ineligible study design |
| 1. Kenealy, T. (2014). Sore throat. Clinical Evidence, 04, 04. | Ineligible study design |
| 1. Keya, T. A., Leela, A., Fernandez, K., Habib, N., & Rashid, M. (2021). Effect of Vitamin C supplements on respiratory tract infections: A systematic review and meta-analysis. Current reviews in clinical and experimental pharmacology. https://doi.org/10.2174/2772432817666211230100723 | Ineligible study design |
| 1. Leung, A. K., Hon, K. L., & Chu, W. C. (2020). Acute bacterial sinusitis in children: an updated review. Drugs in Context, 9. https://doi.org/https://dx.doi.org/10.7573/dic.2020-9-3 | Ineligible study design |
| 1. Leung, A. K. C., Hon, K. L., Wong, A. H. C., & Wong, A. S. (2018). Bacterial conjunctivitis in childhood: Etiology, clinical manifestations, diagnosis, and management. Recent Patents on Inflammation and Allergy Drug Discovery, 12(2), 120-127. https://doi.org/10.2174/1872213X12666180129165718 | Ineligible study design |
| 1. Leung, A. K. C., & Wong, A. H. C. (2017). Acute otitis media in children [Review]. Recent Patents on Inflammation and Allergy Drug Discovery, 11(1), 32-40. https://doi.org/10.2174/1874609810666170712145332 | Ineligible study design |
| 1. Leung, A. K. C., Lam, J. M., Leong, K. F., & Hon, K. L. (2021). Pityriasis Rosea: An Updated Review. Current pediatric reviews, 17(3), 201-211. https://doi.org/10.2174/1573396316666200923161330 | Ineligible study design |
| 1. Levi, J. R., Brody, R. M., McKee-Cole, K., Pribitkin, E., & O'Reilly, R. (2013). Complementary and alternative medicine for pediatric otitis media. International Journal of Pediatric Otorhinolaryngology, 77(6), 926-931. https://doi.org/10.1016/j.ijporl.2013.03.009 | Ineligible study design |
| 1. Liu, W., Jiang, H. L., & Mao, B. (2013). Chinese herbal medicine for postinfectious cough: A systematic review of randomized controlled trials. Evidence-based Complementary and Alternative Medicine, 2013. https://doi.org/10.1155/2013/906765 | Ineligible study design |
| 1. Liu, X. H., Lu, C. L., Wang, L. Q., Shang, Y. X., Stub, T., Kristoffersen, A. E., Norheim, A. J., Musial, F., Alraek, T., Fonnebo, V., & Liu, J. P. (2019). Beneficial effectiveness and safety of homeopathic therapy for upper respiratory tract infection in children: a systematic review. Advances in Integrative Medicine, 6, S91. https://doi.org/10.1016/j.aimed.2019.03.263 | Ineligible study design |
| 1. Lund, V. J. (2008). Therapeutic targets in rhinosinusitis: infection or inflammation? Medscape journal of medicine, 10(4), 105. | Ineligible study design |
| 1. Lund, V. J., Grouin, J. M., Eccles, R., Bouter, C., & Chabolle, F. (2004). Efficacy of fusafungine in acute rhinopharyngitis: a pooled analysis. Rhinology, 42(4), 207-212. | Ineligible study design |
| 1. MacKay, D. N. (1996). Treatment of acute bronchitis in adults without underlying lung disease. Journal of General Internal Medicine, 11(9), 557-562. | Ineligible study design |
| 1. Marchisio, P., Galli, L., Bortone, B., Ciarcia, M., Motisi, M. A., Novelli, A., Pinto, L., Bottero, S., Pignataro, L., Piacentini, G., Mattina, R., Cutrera, R., Varicchio, A., Luigi Marseglia, G., Villani, A., Chiappini, E., & Italian Panel for the Management of Acute Otitis Media in, C. (2019). Updated Guidelines for the Management of Acute Otitis Media in Children by the Italian Society of Pediatrics: Treatment. Pediatric Infectious Disease Journal, 38(12S Suppl), S10-S21. https://doi.org/https://dx.doi.org/10.1097/INF.0000000000002452 | Ineligible study design |
| 1. Marcy, M., Takata, G., Chan, L. S., Shekelle, P., Mason, W., Wachsman, L., Ernst, R., Hay, J. W., Corley, P. M., Morphew, T., Ramicone, E., & Nicholson, C. (2000). Management of acute otitis media. Evidence Report: Technology Assessment (Summary)(15), 1-4. | Ineligible study design |
| 1. Marks, D. H., Prasad, S., De Souza, B., Burns, L. J., & Senna, M. M. (2020). Topical Antiandrogen Therapies for Androgenetic Alopecia and Acne Vulgaris. American Journal of Clinical Dermatology, 21(2), 245-254. https://doi.org/10.1007/s40257-019-00493-z | Ineligible study design |
| 1. Martinez-Gonzalez, N. A., Keizer, E., Plate, A., Coenen, S., Valeri, F., Verbakel, J. Y. J., Rosemann, T., Neuner-Jehle, S., & Senn, O. (2020). Point-of-Care C-Reactive Protein Testing to Reduce Antibiotic Prescribing for Respiratory Tract Infections in Primary Care: Systematic Review and Meta-Analysis of Randomised Controlled Trials. Antibiotics, 9(9), 16. https://doi.org/https://dx.doi.org/10.3390/antibiotics9090610 | Ineligible study design |
| 1. Morris, P. S. (1998). A systematic review of clinical research addressing the prevalence, aetiology, diagnosis, prognosis and therapy of otitis media in Australian Aboriginal children. Journal of Paediatrics and Child Health, 34(6), 487-497. https://doi.org/10.1046/j.1440-1754.1998.00299.x | Ineligible study design |
| 1. Williamson, I. (2007). Otitis media with effusion in children. BMJ clinical evidence, 2007. | Ineligible study design |
| 1. Zentz, S. E. (2011). Care of infants and children with bronchiolitis: A systematic review. Journal of Pediatric Nursing, 26(6), 519-529. https://doi.org/10.1016/j.pedn.2010.07.008 | Ineligible study design |
| 1. Zhao, N., Liu, C., Zhu, C., Dong, X., & Liu, X. (2019). Pidotimod: a review of its pharmacological features and clinical effectiveness in respiratory tract infections. Expert Review of Antiinfective Therapy, 17(10), 803-818. https://doi.org/https://dx.doi.org/10.1080/14787210.2019.1679118 | Ineligible study design |
| 1. Zorzela, L., Ardestani, S. K., McFarland, L. V., & Vohra, S. (2017). Is there a role for modified probiotics as beneficial microbes: A systematic review of the literature. Beneficial Microbes, 8(5), 739-754. https://doi.org/10.3920/BM2017.0032 | Ineligible study design |
| 1. Hajioff, D., & MacKeith, S. (2015). Otitis externa. BMJ Clin Evid, 2015. | Ineligible study design |
| 1. Jackson, E. A., & Geer, K. (2023). Acute Otitis Externa: Rapid Evidence Review. American Family Physician, 107(2), 145-151. | Ineligible study design |
| 1. Berkman, N. D., Wallace, I. F., Steiner, M. J., Harrison, M., Greenblatt, A. M., Lohr, K. N., Kimple, A., & Yuen, A. (2013). Agency for Healthcare Research and Quality, 05. | Ineligible study design |
| 1. Wang, M. X., Koh, J., & Pang, J. (2019). Association between micronutrient deficiency and acute respiratory infections in healthy adults: A systematic review of observational studies. Nutrition Journal, 18(1). https://doi.org/10.1186/s12937-019-0507-6 | Ineligible study design |
| 1. Wark, P. (2015). Bronchitis (acute). BMJ clinical evidence, 2015. | Ineligible study design |
| 1. Hajioff, D., & Mackeith, S. (2010). Otitis externa. BMJ Clin Evid, 2010. | Ineligible study design |
| 1. Mathioudakis, A. G., Chatzimavridou-Grigoriadou, V., Corlateanu, A., & Vestbo, J. (2017). Procalcitonin to guide antibiotic administration in COPD exacerbations: a meta-analysis. European Respiratory Review, 26(143). https://doi.org/https://dx.doi.org/10.1183/16000617.0073-2016 | Ineligible study design |
| 1. Abuelgasim, H., Albury, C., & Lee, J. (2021). Effectiveness of honey for symptomatic relief in upper respiratory tract infections: A systematic review and meta-analysis. BMJ Evidence-Based Medicine, 26(2), 57-64. https://doi.org/10.1136/bmjebm-2020-111336 | Ineligible outcome |
| 1. Ali, A. S., Hasan, S. S., Kow, C. S., & Merchant, H. A. (2021). Lactoferrin reduces the risk of respiratory tract infections: A meta-analysis of randomized controlled trials. Clinical Nutrition ESPEN, 45, 26-32. https://doi.org/10.1016/j.clnesp.2021.08.019 | Ineligible outcome |
| 1. Araujo, G. V., Oliveira Junior, M. H., Peixoto, D. M., & Sarinho, E. S. (2015). Probiotics for the treatment of upper and lower respiratory-tract infections in children: systematic review based on randomized clinical trials. Jornal de Pediatria, 91(5), 413-427. https://doi.org/https://dx.doi.org/10.1016/j.jped.2015.03.002 | Ineligible outcome |
| 1. Buckley Prof, D. I., & Heneghan Prof, C. (2013). Erratum: Duration of symptoms of respiratory tract infections in children: Systematic review(BMJ (Online) (2013) 347 (f7027) DOI:10.1136/bmj.f7027). BMJ (Online), 347. | Ineligible outcome |
| 1. Cao, A. M. Y., Choy, J. P., Mohanakrishnan, L. N., Bain, R. F., & van Driel, M. L. (2013). Chest radiographs for acute lower respiratory tract infections. Cochrane Database of Systematic Reviews(12). https://doi.org/10.1002/14651858.CD009119.pub2 | Ineligible outcome |
| 1. Carr, R. R., & Nahata, M. C. (2006). Complementary and alternative medicine for upper-respiratory-tract infection in children. American Journal of Health-System Pharmacy, 63(1), 33-39. https://doi.org/10.2146/ajhp040613 | Ineligible outcome |
| 1. Chenot, J. F., & Holzinger, F. (2011). Systematic review of clinical trials assessing the effectiveness of ivy leaf (Hedera Helix) for acute upper respiratory tract infections. Evidence-based Complementary and Alternative Medicine, 2011. https://doi.org/10.1155/2011/382789 | Ineligible outcome |
| 1. Fahey, T., Stocks, N., & Thomas, T. (1998). Systematic review of the treatment of upper respiratory tract infection. Archives of Disease in Childhood, 79(3), 225-230. https://doi.org/10.1136/adc.79.3.225 | Ineligible outcome |
| 1. Fixsen, A. (2018). Homeopathy in the Age of Antimicrobial Resistance: Is It a Viable Treatment for Upper Respiratory Tract Infections? Homeopathy, 107(2), 99-114. https://doi.org/10.1055/s-0037-1621745 | Ineligible outcome |
| 1. Grande, A. J., Keogh, J., Silva, V., & Scott, A. M. (2020). Exercise versus no exercise for the occurrence, severity, and duration of acute respiratory infections. Cochrane Database of Systematic Reviews(4). https://doi.org/10.1002/14651858.CD010596.pub3 | Ineligible outcome |
| 1. Guppy, M. P., Mickan, S. M., & Del Mar, C. B. (2004). "Drink plenty of fluids": a systematic review of evidence for this recommendation in acute respiratory infections. BMJ, 328(7438), 499-500. | Ineligible outcome |
| 1. Guppy, M. P., Mickan, S. M., & Del Mar, C. B. (2005). Advising patients to increase fluid intake for treating acute respiratory infections. Cochrane database of systematic reviews (Online)(4), CD004419. | Ineligible outcome |
| 1. Hawke, K., van Driel, M. L., Buffington, B. J., McGuire, T. M., & King, D. (2018). Homeopathic medicinal products for preventing and treating acute respiratory tract infections in children. Cochrane Database of Systematic Reviews(9). https://doi.org/10.1002/14651858.CD005974.pub5 | Ineligible outcome |
| 1. Holzinger, F., & Chenot, J. F. (2011). Systematic review of clinical trials assessing the effectiveness of ivy leaf (hedera helix) for acute upper respiratory tract infections. Evidence-Based Complementary & Alternative Medicine: eCAM, 2011, 382789. https://doi.org/https://dx.doi.org/10.1155/2011/382789 | Ineligible outcome |
| 1. Hu, X. Y., Wu, R. H., Logue, M., Blonde, C., Lai, L. Y., Stuart, B., Flower, A., Fei, Y. T., Moore, M., Liu, J. P., & Lewith, G. (2017). Andrographis paniculata for symptomatic relief of acute respiratory tract infections: A systematic review and meta-analysis. BMC Complementary and Alternative Medicine, 17. https://doi.org/10.1186/s12906-017-1782-4 | Ineligible outcome |
| 1. Huang, Z., Pan, X., Zhou, J., Leung, W. T., Li, C., & Wang, L. (2019). Chinese herbal medicine for acute upper respiratory tract infections and reproductive safety: A systematic review. Bioscience trends, 13(2), 117-129. https://doi.org/10.5582/bst.2018.01298 | Ineligible outcome |
| 1. Hunter, J., Arentz, S., Goldenberg, J., Yang, G., Beardsley, J., Myers, S. P., Mertz, D., & Leeder, S. (2021). Zinc for the prevention or treatment of acute viral respiratory tract infections in adults: A rapid systematic review and meta-analysis of randomised controlled trials. BMJ Open, 11(11). https://doi.org/10.1136/bmjopen-2020-047474 | Ineligible outcome |
| 1. Kamin, W., Funk, P., Seifert, G., Zimmermann, A., & Lehmacher, W. (2018). Eps 7630 is effective and safe in children under 6 years with acute respiratory tract infections: Clinical studies revisited. Current Medical Research and Opinion, 34(3), 475-485. https://doi.org/10.1080/03007995.2017.1402754 | Ineligible outcome |
| 1. Kasyan, V. N., Zaitsev, A. V., Perepanova, T. A., Pivazyan, L. G., Grigoryan, B. L., Kupriyanov, Y. A., Kasyan, G. R., & Pushkar, D. Y. (2021). Systematic review of the efficacy and safety of nifuratel for patients with lower urinary tract infection. Akusherstvo i Ginekologiya (Russian Federation), 2021(6), 168-176. https://doi.org/10.18565/AIG.2021.6.168-176 | Ineligible outcome |
| 1. King, D., Hawke, K., McGuire, T. M., & van Driel, M. (2021). Homeopathic Preparations for Preventing and Treating Acute Upper Respiratory Tract Infections in Children: A Systematic Review and Meta-Analysis. Academic Pediatrics, 21(2), 211-220. https://doi.org/10.1016/j.acap.2020.07.016 | Ineligible outcome |
| 1. King, S., Glanville, J., Sanders, M. E., Fitzgerald, A., & Varley, D. (2014). Effectiveness of probiotics on the duration of illness in healthy children and adults who develop common acute respiratory infectious conditions: A systematic review and meta-analysis. British Journal of Nutrition, 112(1), 41-54. https://doi.org/10.1017/S0007114514000075 | Ineligible outcome |
| 1. Nikolaeva, S. V., Melnikova, V. V., & Usenko, D. V. (2019). Efficacy of aromatherapy in the treatment and prevention of acute respiratory infections in children. Voprosy Prakticheskoi Pediatrii, 14(1), 63-69. https://doi.org/10.20953/1817-7646-2019-1-63-69 | Ineligible outcome |
| 1. Sierocinski, E., Holzinger, F., & Chenot, J. F. (2021). Ivy leaf (Hedera helix) for acute upper respiratory tract infections: an updated systematic review. European Journal of Clinical Pharmacology, 77(8), 1113-1122. https://doi.org/10.1007/s00228-021-03090-4 | Ineligible outcome |
| 1. Timmer, A., Günther, J., Motschall, E., Rücker, G., Antes, G., & Kern, W. V. (2013). Pelargonium sidoides extract for treating acute respiratory tract infections. Cochrane Database of Systematic Reviews(10). https://doi.org/10.1002/14651858.CD006323.pub3 | Ineligible outcome |
| 1. Vouloumanou, E. K., Makris, G. C., Karageorgopoulos, D. E., & Falagas, M. E. (2009). Probiotics for the prevention of respiratory tract infections: a systematic review. International Journal of Antimicrobial Agents, 34(3), 197.e191-197.e110. https://doi.org/10.1016/j.ijantimicag.2008.11.005 | Ineligible outcome |
| 1. Wang, Y., Li, X., Ge, T., Xiao, Y., Liao, Y., Cui, Y., Zhang, Y., Ho, W., Yu, G., & Zhang, T. (2016). Probiotics for prevention and treatment of respiratory tract infections in children: A systematic review and meta-analysis of randomized controlled trials. Medicine (United States), 95(31). https://doi.org/10.1097/MD.0000000000004509 | Ineligible outcome |
| 1. Alarcon-Andrade, G., & Cifuentes, L. (2018). Do inhaled corticosteroids have a role for bronchiolitis? Medwave, 18(2), e7183. https://doi.org/https://dx.doi.org/10.5867/medwave.2018.02.7182 | Ineligible outcome |
| 1. AlBalawi, Z. H., Othman, S. S., & AlFaleh, K. (2013). Intranasal ipratropium bromide for the common cold. Cochrane Database of Systematic Reviews(6). https://doi.org/10.1002/14651858.CD008231.pub3 | Ineligible outcome |
| 1. Arroll, B., & Kenealy, T. (2006). Are antibiotics effective for acute purulent rhinitis? Systematic review and meta-analysis of placebo controlled randomised trials. BMJ, 333(7562), 279. | Ineligible outcome |
| 1. Burgstaller, J. M., Steurer, J., Holzmann, D., Geiges, G., & Soyka, M. B. (2016). Antibiotic efficacy in patients with a moderate probability of acute rhinosinusitis: a systematic review. European Archives of Oto-Rhino-Laryngology, 273(5), 1067-1077. https://doi.org/https://dx.doi.org/10.1007/s00405-015-3506-z | Ineligible outcome |
| 1. Butler, C. C., & Van Der Voort, J. H. (2002). Oral or topical nasal steroids for hearing loss associated with otitis media with effusion in children. Cochrane database of systematic reviews (Online)(4), CD001935. | Ineligible outcome |
| 1. Cai, T., Anceschi, U., Tamanini, I., Migno, S., Rizzo, M., Liguori, G., Garcia-Larrosa, A., Palmieri, A., Verze, P., Mirone, V., & Bjerklund Johansen, T. E. (2021). Xyloglucan, Hibiscus and Propolis in the Management of Uncomplicated Lower Urinary Tract Infections: A Systematic Review and Meta-Analysis. Antibiotics, 11(1), 23. https://doi.org/https://dx.doi.org/10.3390/antibiotics11010014 | Ineligible outcome |
| 1. Carey, M. R., Vaughn, V. M., Mann, J., Townsend, W., Chopra, V., & Patel, P. K. (2020). Is Non-Steroidal Anti-Inflammatory Therapy Non-Inferior to Antibiotic Therapy in Uncomplicated Urinary Tract Infections: a Systematic Review. Journal of General Internal Medicine, 35(6), 1821-1829. https://doi.org/10.1007/s11606-020-05745-x | Ineligible outcome |
| 1. Coker, T. R., Chan, L. S., Newberry, S. J., Limbos, M. A., Suttorp, M. J., Shekelle, P. G., & Takata, G. S. (2010). Diagnosis, microbial epidemiology, and antibiotic treatment of acute otitis media in children: A systematic review. JAMA - Journal of the American Medical Association, 304(19), 2161-2169. https://doi.org/10.1001/jama.2010.1651 | Ineligible outcome |
| 1. Coleman, C., & Moore, M. (2008). Decongestants and antihistamines for acute otitis media in children. Cochrane Database of Systematic Reviews(3). https://doi.org/10.1002/14651858.CD001727.pub4 | Ineligible outcome |
| 1. Cronin, M. J., Khan, S., & Saeed, S. (2013). The role of antibiotics in the treatment of acute rhinosinusitis in children: a systematic review. Archives of Disease in Childhood, 98(4), 299-303. https://doi.org/https://dx.doi.org/10.1136/archdischild-2012-302983 | Ineligible outcome |
| 1. Damoiseaux, R. A., & Rovers, M. M. (2011). AOM in children. BMJ clinical evidence, 2011. https://www.embase.com/search/results?subaction=viewrecord&id=L611782221&from=export | Ineligible outcome |
| 1. de Ferranti, S. D., Ioannidis, J. P., Lau, J., Anninger, W. V., & Barza, M. (1998). Are amoxycillin and folate inhibitors as effective as other antibiotics for acute sinusitis? A meta-analysis. BMJ, 317(7159), 632-637. | Ineligible outcome |
| 1. Del Mar, C., Glasziou, P., & Hayem, M. (1997). Are antibiotics indicated as initial treatment for children with acute otitis media? A meta-analysis. BMJ, 314(7093), 1526-1529. | Ineligible outcome |
| 1. Douglas, R. M., Chalker, E. B., & Treacy, B. (2000). Vitamin C for preventing and treating the common cold. Cochrane Database of Systematic Reviews(2), CD000980. | Ineligible outcome |
| 1. Fahey, T., & Howie, J. (2001). Re-evaluation of a randomized controlled trial of antibiotics for minor respiratory illness in general practice. Family Practice, 18(3), 246-248. https://doi.org/10.1093/fampra/18.3.246 | Ineligible outcome |
| 1. Falagas, M. E., Giannopoulou, K. P., Vardakas, K. Z., Dimopoulos, G., & Karageorgopoulos, D. E. (2008). Comparison of antibiotics with placebo for treatment of acute sinusitis: a meta-analysis of randomised controlled trials. The Lancet Infectious Diseases, 8(9), 543-552. https://doi.org/https://dx.doi.org/10.1016/S1473-3099(08)70202-0 | Ineligible outcome |
| 1. Falagas, M. E., Kotsantis, I. K., Vouloumanou, E. K., & Rafailidis, P. I. (2009). Antibiotics versus placebo in the treatment of women with uncomplicated cystitis: a meta-analysis of randomized controlled trials. Journal of Infection, 58(2), 91-102. https://doi.org/https://dx.doi.org/10.1016/j.jinf.2008.12.009 | Ineligible outcome |
| 1. Foxlee, R., Johansson, A. C., Wejfalk, J., Dooley, L., & Del Mar, C. B. (2006). Topical analgesia for acute otitis media. Cochrane Database of Systematic Reviews(3). https://doi.org/10.1002/14651858.CD005657.pub2 | Ineligible outcome |
| 1. George, A., & Rubin, G. (2003). A systematic review and meta-analysis of treatments for impetigo. British Journal of General Practice, 53(491), 480-487. | Ineligible outcome |
| 1. Gevorgyan, A., Segboer, C., Gorissen, R., van Drunen, C. M., & Fokkens, W. (2015). Capsaicin for non‐allergic rhinitis. Cochrane Database of Systematic Reviews(7). https://doi.org/10.1002/14651858.CD010591.pub2 | Ineligible outcome |
| 1. Guarch Ibáñez, B., Buñuel Álvarez, J. C., López Bermejo, A., & Mayol Canals, L. (2011). The role of antibiotics in acute sinusitis: A systematic review and metaanalysis. Anales de Pediatria, 74(3), 154-160. https://doi.org/10.1016/j.anpedi.2010.10.011 | Ineligible outcome |
| 1. Holm, N. H., Rusan, M., & Ovesen, T. (2020). Acute otitis media and antibiotics – a systematic review. Danish Medical Journal, 67(11), 1-10. | Ineligible outcome |
| 1. Jackson, J. L., Peterson, C., & Lesho, E. (1997). A meta-analysis of zinc salts lozenges and the common cold. Archives of Internal Medicine, 157(20), 2373-2376. | Ineligible outcome |
| 1. Kellner, J. D., Ohlsson, A., Gadomski, A. M., & Wang, E. E. (1996). Efficacy of bronchodilator therapy in bronchiolitis. A meta-analysis. Archives of Pediatrics & Adolescent Medicine, 150(11), 1166-1172. | Ineligible outcome |
| 1. Kodjikian, L., Lafuma, A., Khoshnood, B., Laurendeau, C., & Berdeaux, G. (2010). [Efficacy of moxifloxacin in treating bacterial conjunctivitis: a meta-analysis]. Journal Francais d Opthalmologie, 33(4), 227-233. https://doi.org/https://dx.doi.org/10.1016/j.jfo.2010.02.001 | Ineligible outcome |
| 1. Linde, K., Barrett, B., Wölkart, K., Bauer, R., & Melchart, D. (2006). Echinacea for preventing and treating the common cold. Cochrane Database of Systematic Reviews(1). https://doi.org/10.1002/14651858.CD000530.pub2 | Ineligible outcome |
| 1. Marshall, I. (2000). Zinc for the common cold. Cochrane database of systematic reviews (Online)(2), CD001364. https://www.embase.com/search/results?subaction=viewrecord&id=L31317753&from=export | Ineligible outcome |
| 1. Marshall, S. (1998). Zinc gluconate and the common cold: Review of randomized controlled trials. Canadian Family Physician, 44(MAY.), 1037-1042. | Ineligible outcome |
| 1. Pignataro, O., Pignataro, L. D., Gallus, G., Calori, G., & Cordaro, C. I. (1996). Otitis media with effusion and S-carboxymethylcysteine and/or its lysine salt: A critical overview. International Journal of Pediatric Otorhinolaryngology, 35(3), 231-241. https://doi.org/10.1016/0165-5876(95)01315-6 | Ineligible outcome |
| 1. Seresirikachorn, K., Khattiyawittayakun, L., Chitsuthipakorn, W., & Snidvongs, K. (2018). Antihistamines for treating rhinosinusitis: systematic review and meta-analysis of randomised controlled studies. Journal of Laryngology & Otology, 132(2), 105-110. https://doi.org/https://dx.doi.org/10.1017/S002221511700192X | Ineligible outcome |
| 1. Takata, G. S., Chan, L. S., Shekelle, P., Morton, S. C., Mason, W., & Marcy, S. M. (2001). Evidence assessment of management of acute otitis media: I. The role of antibiotics in treatment of uncomplicated acute otitis media. Pediatrics, 108(2), 239-247. | Ineligible outcome |
| 1. Epling, J. (2012). Bacterial conjunctivitis. BMJ clinical evidence, 2012. | Ineligible outcome |
| 1. Farley, R., Spurling, G. K., Eriksson, L., & Del Mar, C. B. (2014). Antibiotics for bronchiolitis in children under two years of age. Cochrane Database of Systematic Reviews, 2014(10). https://doi.org/10.1002/14651858.CD005189.pub4 | Ineligible outcome |
| 1. Alarcon-Andrade, G., Bravo-Soto, G., & Rada, G. (2017). Are systemic corticosteroids useful for the management of acute pharyngitis? Medwave, 17(9), e7111. https://doi.org/https://dx.doi.org/10.5867/medwave.2017.09.7111 | Ineligible outcome |
| 1. Alarcon-Andrade, G., & Cifuentes, L. (2018). Should systemic corticosteroids be used for bronchiolitis? Medwave, 18(3), e7207. https://doi.org/https://dx.doi.org/10.5867/medwave.2018.03.7206 | Ineligible outcome |
| 1. Barrons, R., & Tassone, D. (2008). Use of Lactobacillus probiotics for bacterial genitourinary infections in women: A review. Clinical Therapeutics, 30(3), 453-468. https://doi.org/10.1016/j.clinthera.2008.03.013 | Ineligible outcome |
| 1. Bedane, C. (1997). [Acute bacterial skin infections in the adult]. Annales de Dermatologie et de Venereologie, 124(1), 57-60. | Ineligible outcome |
| 1. Bergmann, M., Haasenritter, J., Beidatsch, D., Schwarm, S., Hörner, K., Bösner, S., Grevenrath, P., Schmidt, L., Viniol, A., Donner-Banzhoff, N., & Becker, A. (2021). Coughing children in family practice and primary care: a systematic review of prevalence, aetiology and prognosis. BMC Pediatrics, 21(1). https://doi.org/10.1186/s12887-021-02739-4 | Ineligible outcome |
| 1. Blom, D., Ermers, M., Bont, L., Van Aalderen, W. M. C., & Van Woensel, J. B. M. (2007). Inhaled corticosteroids during acute bronchiolitis in the prevention of post-bronchiolitic wheezing. Cochrane Database of Systematic Reviews(1). https://doi.org/10.1002/14651858.CD004881.pub2 | Ineligible outcome |
| 1. Cheng, Y., Gao, B., Jin, Y., Xu, N., & Guo, T. (2018). Acupuncture for common cold: A systematic review and meta-analyze protocol. Medicine, 97(10), e0061. https://doi.org/https://dx.doi.org/10.1097/MD.0000000000010061 | Ineligible outcome |
| 1. D'Agostino, R. B., Sr., Weintraub, M., Russell, H. K., Stepanians, M., D'Agostino, R. B., Jr., Cantilena, L. R., Jr., Graumlich, J. F., Maldonado, S., Honig, P., & Anello, C. (1998). The effectiveness of antihistamines in reducing the severity of runny nose and sneezing: a meta-analysis. Clinical Pharmacology & Therapeutics, 64(6), 579-596. | Ineligible outcome |
| 1. Gahlawat, G., Tesfaye, W., Bushell, M., Abrha, S., Peterson, G. M., Mathew, C., Sinnollareddy, M., McMillan, F., Samarawickrema, I., Calma, T., Chang, A. Y., Engelman, D., Steer, A., & Thomas, J. (2021). Emerging Treatment Strategies for Impetigo in Endemic and Nonendemic Settings: A Systematic Review. Clinical Therapeutics, 43(6), 986-1006. https://doi.org/https://dx.doi.org/10.1016/j.clinthera.2021.04.013 | Ineligible outcome |
| 1. Galindo, E., & Hebert, A. A. (2021). A comparative review of current topical antibiotics for impetigo. Expert Opinion on Drug Safety, 20(6), 677-683. https://doi.org/https://dx.doi.org/10.1080/14740338.2021.1902502 | Ineligible outcome |
| 1. Gallant, J. N., Basem, J. I., Turner, J. H., Shannon, C. N., & Virgin, F. W. (2018). Nasal saline irrigation in pediatric rhinosinusitis: A systematic review. International Journal of Pediatric Otorhinolaryngology, 108, 155-162. https://doi.org/10.1016/j.ijporl.2018.03.001 | Ineligible outcome |
| 1. Guo, R., Canter, P. H., & Ernst, E. (2006). Herbal medicines for the treatment of rhinosinusitis: A systematic review. Otolaryngology - Head and Neck Surgery, 135(4), 496-506. https://doi.org/10.1016/j.otohns.2006.06.1254 | Ineligible outcome |
| 1. Hildenbrand, T., Weber, R., Heubach, C., & Mosges, R. (2011). [Nasal douching in acute rhinosinusitis]. Laryngo- Rhino- Otologie, 90(6), 346-351. https://doi.org/https://dx.doi.org/10.1055/s-0031-1275317 | Ineligible outcome |
| 1. Jung, J., Park, J., Choi, J. Y., & Lee, J. A. (2018). Soshiho-tang for treating common cold in children younger than 12 years: A systematic review and meta-analysis of randomized controlled trials. Medicine, 97(45), e13045. https://doi.org/https://dx.doi.org/10.1097/MD.0000000000013045 | Ineligible outcome |
| 1. Keller, S., König, V., & Mösges, R. (2014). Thermal water applications in the treatment of upper respiratory tract diseases: A systematic review and meta-analysis. Journal of Allergy, 2014. https://doi.org/10.1155/2014/943824 | Ineligible outcome |
| 1. King, D., Mitchell, B., Williams, C. P., & Spurling, G. K. P. (2015). Saline nasal irrigation for acute upper respiratory tract infections. Cochrane Database of Systematic Reviews(4). https://doi.org/10.1002/14651858.CD006821.pub3 | Ineligible outcome |
| 1. Kollar, C., Schneider, H., Waksman, J., & Krusinska, E. (2007). Meta-analysis of the efficacy of a single dose of phenylephrine 10 mg compared with placebo in adults with acute nasal congestion due to the common cold. Clinical Therapeutics, 29(6), 1057-1070. | Ineligible outcome |
| 1. List, M., Headlee, D., & Kondratuk, K. (2016). Treatment of Skin Abscesses: A Review of Wound Packing and Post-Procedural Antibiotics. South Dakota medicine : the journal of the South Dakota State Medical Association, 69(3), 113-119. | Ineligible outcome |
| 1. Moretti, M., & Pozzi, E. (2017). Erdosteine is effective as a treatment for acute exacerbation of COPD: a systematic review of clinical trials. European Respiratory Journal, 50. https://doi.org/10.1183/1393003.congress-2017.PA685 | Ineligible outcome |
| 1. Munoz-Osores, E., & Arenas, D. (2017). What is the effectiveness of systemic corticosteroids in children with croup? Medwave, 17(Suppl2), e6965. https://doi.org/https://dx.doi.org/10.5867/medwave.2017.6965 | Ineligible outcome |
| 1. Nitsche, M. P., & Carreno, M. (2016). Is honey an effective treatment for acute cough in children? Medwave, 16 Suppl 2, e6454. https://doi.org/https://dx.doi.org/10.5867/medwave.2016.6454 | Ineligible outcome |
| 1. O'Sullivan, J. W., Harvey, R. T., Glasziou, P. P., & McCullough, A. (2016). Written information for patients (or parents of child patients) to reduce the use of antibiotics for acute upper respiratory tract infections in primary care. Cochrane Database of Systematic Reviews(11). https://doi.org/10.1002/14651858.CD011360.pub2 | Ineligible outcome |
| 1. Shokri-Mashhadi, N., Kazemi, M., Saadat, S., & Moradi, S. (2021). Effects of select dietary supplements on the prevention and treatment of viral respiratory tract infections: a systematic review of randomized controlled trials. Expert Review of Respiratory Medicine, 15(6), 805-821. https://doi.org/10.1080/17476348.2021.1918546 | Ineligible outcome |
| 1. Singh, M., & Das, R. R. (2015). Zinc for the common cold. Cochrane Database of Systematic Reviews, 2015(4). https://doi.org/10.1002/14651858.CD001364.pub5 | Ineligible outcome |
| 1. Singh, M., Singh, M., Jaiswal, N., & Chauhan, A. (2017). Heated, humidified air for the common cold. Cochrane Database of Systematic Reviews(8). https://doi.org/10.1002/14651858.CD001728.pub6 | Ineligible outcome |
| 1. Skevaki, C. L., Galani, I. E., Pararas, M. V., Giannopoulou, K. P., & Tsakris, A. (2011). Treatment of viral conjunctivitis with antiviral drugs. Drugs, 71(3), 331-347. https://doi.org/10.2165/11585330-000000000-00000 | Ineligible outcome |
| 1. Somani, R., & Evans, M. F. (2001). Role of glucocorticoids in treating croup. Canadian Family Physician, 47, 733-735. | Ineligible outcome |
| 1. Spurling, G. K. P., Del Mar, C. B., Dooley, L., Clark, J., & Askew, D. A. (2017). Delayed antibiotic prescriptions for respiratory infections. Cochrane Database of Systematic Reviews(9). https://doi.org/10.1002/14651858.CD004417.pub5 | Ineligible outcome |
| 1. Steurer-Stey, C., Lagler, L., Straub, D. A., Steurer, J., & Bachmann, L. M. (2007). Oral purified bacterial extracts in acute respiratory tract infections in childhood: a systematic quantitative review. European Journal of Pediatrics, 166(4), 365-376. | Ineligible outcome |
| 1. Stuart, B., Hounkpatin, H., Becque, T., Yao, G., Zhu, S., Alonso-Coello, P., Altiner, A., Arroll, B., Böhning, D., Bostock, J., Bucher, H. C., Chao, J., De La Poza, M., Francis, N., Gillespie, D., Hay, A. D., Kenealy, T., Löffler, C., McCormick, D. P., . . . Little, P. (2021). Delayed antibiotic prescribing for respiratory tract infections: Individual patient data meta-analysis. The BMJ, 373. https://doi.org/10.1136/bmj.n808 | Ineligible outcome |
| 1. Wang, J., Xu, H., Liu, P., & Li, M. (2017). Network meta-analysis of success rate and safety in antibiotic treatments of bronchitis. International Journal of COPD, 12, 2391-2405. https://doi.org/https://dx.doi.org/10.2147/COPD.S139521 | Ineligible outcome |
| 1. Wilcox, C. R., Stuart, B., Leaver, H., Lown, M., Willcox, M., Moore, M., & Little, P. (2019). Effectiveness of the probiotic Streptococcus salivarius K12 for the treatment and/or prevention of sore throat: a systematic review. Clinical Microbiology and Infection, 25(6), 673-680. https://doi.org/10.1016/j.cmi.2018.12.031 | Ineligible outcome |
| 1. Woods, J. A., Wheeler, J. S., Finch, C. K., & Pinner, N. A. (2014). Corticosteroids in the treatment of acute exacerbations of chronic obstructive pulmonary disease. International Journal of COPD, 9, 421-430. https://doi.org/10.2147/COPD.S51012 | Ineligible outcome |
| 1. Wopker, P. M., Schwermer, M., Sommer, S., Längler, A., Fetz, K., Ostermann, T., & Zuzak, T. J. (2020). Complementary and alternative medicine in the treatment of acute bronchitis in children: A systematic review. Complementary Therapies in Medicine, 49. https://doi.org/10.1016/j.ctim.2019.102217 | Ineligible outcome |
| 1. Yan, L. J., Wang, Z. J., Fang, M., Lan, H. D., Moore, M., Willcox, M., Trill, J., Hu, X. Y., & Liu, J. P. (2021). Bupleuri radix for Acute Uncomplicated Respiratory Tract Infection: A Systematic Review of Randomized Controlled Trials. Frontiers in Pharmacology, 12, 787084. https://doi.org/https://dx.doi.org/10.3389/fphar.2021.787084 | Ineligible outcome |
| 1. Zhang, K., Xie, K., Zhang, C., Liang, Y., Chen, Z., & Wang, H. (2022). C-reactive protein testing to reduce antibiotic prescribing for acute respiratory infections in adults: a systematic review and meta-analysis. Journal of Thoracic Disease, 14(1), 123-134. https://doi.org/10.21037/jtd-21-705 | Ineligible outcome |
| 1. Lopes, L. C., Silva, M. C., Motta, C. B., Macho Quiros, A., Biavatti, M. W., de Oliveira, J. C., & Guyatt, G. (2014). Brazilian medicinal plants to treat upper respiratory tract and bronchial illness: systematic review and meta-analyses-study protocol. BMJ Open, 4(7), e005267. https://doi.org/https://dx.doi.org/10.1136/bmjopen-2014-005267 | Ineligible outcome |
| 1. Nahas, R., & Balla, A. (2011). Complementary and alternative medicine for prevention and treatment of the common cold. Canadian Family Physician, 57(1), 31-36. | Ineligible outcome |
| 1. Ng, C., Foran, M., & Koyfman, A. (2014). Do glucocorticoids provide benefit to children with bronchiolitis? Annals of Emergency Medicine, 64(4), 389-391. https://doi.org/https://dx.doi.org/10.1016/j.annemergmed.2014.01.026 | Ineligible outcome |
| 1. O'Neill, P., Roberts, T., & Bradley Stevenson, C. (2007). Otitis media in children (acute). BMJ clinical evidence, 2007. | Ineligible outcome |
| 1. Oduwole, O., Meremikwu, M. M., Oyo-Ita, A., & Udoh, E. E. (2014). Honey for acute cough in children. Cochrane Database of Systematic Reviews, 2014(12). https://doi.org/10.1002/14651858.CD007094.pub4 | Ineligible outcome |
| 1. Padberg, J. (2012). [The common cold: are non-steroidal antiinflammatory drugs effective?]. Deutsche Medizinische Wochenschrift, 137(41), 2087. https://doi.org/https://dx.doi.org/10.1055/s-0032-1329129 | Ineligible outcome |
| 1. Perić, A., Soklič Košak, T., Aleksić, A., Kopacheva-Barsova, G., & Perić, A. V. (2021). Efficacy and safety of myrtol® standardized in the treatment of acute and chronic rhinosinusitis: A review of literature. Erciyes Medical Journal, 43(1), 3-8. https://doi.org/10.14744/etd.2020.34467 | Ineligible outcome |
| 1. Quidel, S., Gomez, E., Bravo-Soto, G., & Ortigoza, A. (2018). What are the effects of vitamin C on the duration and severity of the common cold? Medwave, 18(6), e7261. https://doi.org/https://dx.doi.org/10.5867/medwave.2018.06.7260 | Ineligible outcome |
| 1. Schachner, L., Andriessen, A., Bhatia, N., Grada, A., & Patele, D. (2019). Topical Ozenoxacin Cream 1% for Impetigo: A Review. Journal of Drugs in Dermatology: JDD, 18(7), 655-661. | Ineligible outcome |
| 1. Schachner, L. A., Lynde, C. W., Kircik, L. H., Torrelo, A., Hohl, D., Kwong, P., Oza, V., Andriessen, A., & Hebert, A. A. (2021). Treatment of Impetigo and Antimicrobial Resistance. Journal of Drugs in Dermatology: JDD, 20(4), 366-372. https://doi.org/https://dx.doi.org/10.36849/JDD.2021.5795 | Ineligible outcome |
| 1. Schmelzle, J., Birtwhistle, R. V., & Tan, A. K. (2008). Acute otitis media in children with tympanostomy tubes. Canadian Family Physician, 54(8), 1123-1127. | Ineligible outcome |
| 1. Schuetz, P., Wirz, Y., Sager, R., Christ‐Crain, M., Stolz, D., Tamm, M., Bouadma, L., Luyt, C. E., Wolff, M., Chastre, J., & et al. (2017). Procalcitonin to initiate or discontinue antibiotics in acute respiratory tract infections. Cochrane Database of Systematic Reviews(10). https://doi.org/10.1002/14651858.CD007498.pub3 | Ineligible outcome |
| 1. Senn, N. (2013). [Not too eager prescribing vitamin C for the common cold!]. Revue Medicale Suisse, 9(401), 1854-1855. | Ineligible outcome |
| 1. Shaheen, G., Akram, M., Jabeen, F., Ali Shah, S. M., Munir, N., Daniyal, M., Riaz, M., Tahir, I. M., Ghauri, A. O., Sultana, S., Zainab, R., & Khan, M. (2019). Therapeutic potential of medicinal plants for the management of urinary tract infection: A systematic review. Clinical and Experimental Pharmacology and Physiology, 46(7), 613-624. https://doi.org/10.1111/1440-1681.13092 | Ineligible outcome |
| 1. Shekelle, P., Takata, G., Chan, L. S., Mangione-Smith, R., Corley, P. M., Morphew, T., & Morton, S. (2002). Diagnosis, natural history, and late effects of otitis media with effusion. Evidence Report: Technology Assessment (Summary)(55), 1-5. | Ineligible outcome |
| 1. Sinopoli, A., Isonne, C., Santoro, M. M., & Baccolini, V. (2022). The effects of orally administered lactoferrin in the prevention and management of viral infections: A systematic review. Reviews in Medical Virology, 32(1), e2261. https://doi.org/https://dx.doi.org/10.1002/rmv.2261 | Ineligible outcome |
| 1. Smabrekke, L., & Melbye, H. (2009). [Pharmacological treatment of acute cough]. Tidsskrift for Den Norske Laegeforening, 129(10), 998-999. https://doi.org/https://dx.doi.org/10.4045/tidsskr.08.0438 | Ineligible outcome |
| 1. Smith, M. J. (2013). Evidence for the diagnosis and treatment of acute uncomplicated sinusitis in children: A systematic review. Pediatrics, 132(1), e284-e296. https://doi.org/10.1542/peds.2013-1072 | Ineligible outcome |
| 1. Vahid, F., & Rahmani, D. (2021). Can an anti-inflammatory diet be effective in preventing or treating viral respiratory diseases? A systematic narrative review. Clinical Nutrition ESPEN, 43, 9-15. https://doi.org/10.1016/j.clnesp.2021.04.009 | Ineligible outcome |
| 1. van den Broek, M. F., Gudden, C., Kluijfhout, W. P., Stam-Slob, M. C., Aarts, M. C., Kaper, N. M., & van der Heijden, G. J. (2014). No evidence for distinguishing bacterial from viral acute rhinosinusitis using symptom duration and purulent rhinorrhea: a systematic review of the evidence base. Otolaryngology - Head & Neck Surgery, 150(4), 533-537. https://doi.org/https://dx.doi.org/10.1177/0194599814522595 | Ineligible outcome |
| 1. Vassilev, Z. P., Kabadi, S., & Villa, R. (2010). Safety and efficacy of over-the-counter cough and cold medicines for use in children. Expert Opinion on Drug Safety, 9(2), 233-242. https://doi.org/https://dx.doi.org/10.1517/14740330903496410 | Ineligible outcome |
| 1. Visscher, K. L., Hutnik, C. M., & Thomas, M. (2009). Evidence-based treatment of acute infective conjunctivitis: Breaking the cycle of antibiotic prescribing. Canadian Family Physician, 55(11), 1071-1075. | Ineligible outcome |
| 1. Vitter, J. S. (2011). Do antibiotics improve the treatment of acute otitis media? American Family Physician, 84(9). | Ineligible outcome |
| 1. Wang, M. X., Win, S. S., & Pang, J. (2020). Zinc supplementation reduces common cold duration among healthy adults: A systematic review of randomized controlled trials with micronutrients supplementation. American Journal of Tropical Medicine and Hygiene, 103(1), 86-99. https://doi.org/10.4269/ajtmh.19-0718 | Ineligible outcome |
| 1. Moshtagh, M., & Amiri, R. (2020). Role of zinc supplementation in the improvement of acute respiratory infections among iranian children: A systematic review. Tanaffos, 19(1), 1-9. | Ineligible outcome |
| 1. Becker, L., Glazier, R., McIsaac, W., & Smucny, J. (2000). Antibiotics for acute bronchitis. Cochrane database of systematic reviews (Online)(2), CD000245. | Ineligible outcome |
| 1. Becker, L. A., Hom, J., Villasis-Keever, M., & van der Wouden, J. C. (2011). Beta2-agonists for acute bronchitis. Cochrane database of systematic reviews (Online)(7), CD001726. | Ineligible outcome |
| 1. Zalmanovici Trestioreanu, A., Lador, A., Sauerbrun‐Cutler, M. T., & Leibovici, L. (2015). Antibiotics for asymptomatic bacteriuria. Cochrane Database of Systematic Reviews(4). https://doi.org/10.1002/14651858.CD009534.pub2 | Ineligible population |
| 1. Neilson, L. J., & Hussain, S. S. (2008). Management of granular myringitis: a systematic review. J Laryngol Otol, 122(1), 3-10. https://doi.org/10.1017/s0022215107008924 | Ineligible population |
| 1. Morris, P., & Leach, A. (2002). Antibiotics for persistent nasal discharge (rhinosinusitis) in children. Cochrane Database of Systematic Reviews(4), CD001094. | Ineligible population |
| 1. Chu, S., Michelle, L., Ekelem, C., Sung, C. T., Rojek, N., & Mesinkovska, N. A. (2021). Oral isotretinoin for the treatment of dermatologic conditions other than acne: a systematic review and discussion of future directions. Archives of Dermatological Research, 313(6), 391-430. https://doi.org/https://dx.doi.org/10.1007/s00403-020-02152-4 | Ineligible population |
| 1. Kua, K. P., & Lee, S. W. (2017). Complementary and alternative medicine for the treatment of bronchiolitis in infants: A systematic review. PLoS ONE [Electronic Resource], 12(2), e0172289. https://doi.org/https://dx.doi.org/10.1371/journal.pone.0172289 | Ineligible |
| 1. Mohammed, A. A., Hamdy, T. A., Askoura, A. M., & Abdulhakim, A. M. (2021). Role of intratympanic steroid injection in treatment of otitis media with effusion: A systematic review/Meta -Analysis. QJM, 114(SUPPL 1). https://doi.org/10.1093/qjmed/hcab094.003 | no placebo |
| 1. Wei, J., Ni, J., Wu, T., Chen, X., Duan, X., Liu, G., Yiao, J., Wang, Q., Zhen, J., & Zhou, L. (2006). A systematic review of Chinese medicinal herbs for acute bronchitis. Journal of Alternative & Complementary Medicine, 12(2), 159-169. | no placebo |
| 1. Weinstein, M. E., & Axelrod, D. (2010). The role of inhaled corticosteroids in the treatment of virus- triggered wheezing in pediatric patients. Journal of Allergy and Clinical Immunology, 125(2), AB199. https://doi.org/10.1016/j.jaci.2009.12.776 | no placebo |
| 1. Wren, C., Bell, E., & Eiland, L. S. (2018). Ozenoxacin: A Novel Topical Quinolone for Impetigo. Annals of Pharmacotherapy, 52(12), 1233-1237. https://doi.org/https://dx.doi.org/10.1177/1060028018786510 | No placebo |
| 1. Wu, G., Abraham, T., & Saad, N. (2014). Role of tigecycline for the treatment of urinary tract infections. Journal of Pharmacy Technology, 30(3), 87-92. https://doi.org/10.1177/8755122513519332 | no placebo |
| 1. Mistry, K., Sharma, S., Patel, M., Grindlay, D., Janjuha, R., Smart, P., & Levell, N. J. (2021). Clinical response to antibiotic regimens in lower limb cellulitis: a systematic review. Clinical & Experimental Dermatology, 46(1), 42-49. https://doi.org/https://dx.doi.org/10.1111/ced.14398 | No placebo |
| 1. Abes, G., Espallardo, N., Tong, M., Subramaniam, K. N., Hermani, B., Lasiminigrum, L., & Anggraeni, R. (2003). A systematic review of the effectiveness of ofloxaxin otic solution for the treatment of suppurative otitis media. Orl; Journal of Oto-Rhino-Laryngology & its Related Specialties, 65(2), 106-116. https://doi.org/10.1159/000070775 | No placebo group |
| 1. Alfaresi, M., Hassan, K., & Alnjadat, R. M. H. (2019). Single-dose fosfomycin trometamol versus other antimicrobial regimens for treatment of uncomplicated lower urinary tract infection: A systematic review and meta-analysis. Open Microbiology Journal, 13(1), 193-199. https://doi.org/10.2174/1874285801913010193 | No placebo group |
| 1. Cai, T., Tamanini, I., Tascini, C., Köves, B., Bonkat, G., Gacci, M., Novelli, A., Horcajada, J. P., Bjerklund Johansen, T. E., & Zanel, G. (2020). Fosfomycin Trometamol versus Comparator Antibiotics for the Treatment of Acute Uncomplicated Urinary Tract Infections in Women: A Systematic Review and Meta-Analysis. The Journal of urology, 203(3), 570-578. https://doi.org/10.1097/JU.0000000000000620 | No placebo group |
| 1. Chang, M. H., & Fung, H. B. (2010). Besifloxacin: a topical fluoroquinolone for the treatment of bacterial conjunctivitis. Clinical Therapeutics, 32(3), 454-471. https://doi.org/https://dx.doi.org/10.1016/j.clinthera.2010.03.013 | No placebo group |
| 1. Chen, S. Q., Yang, X. Y., Tang, X. Y., Li, M., Liu, S., Liang, J., Zhang, X. Y., & Shang, H. C. (2017). [Systematic review of Chuankezhi injection for treating acute exacerbation of chronic obstructive pulmonary disease]. Zhongguo Zhong Yao Za Zhi, 42(14), 2789-2795. https://doi.org/10.19540/j.cnki.cjcmm.20170523.009 | No placebo group |
| 1. Cox, V. C., & Zed, P. J. (2004). Once-daily cefazolin and probenecid for skin and soft tissue infections. Annals of Pharmacotherapy, 38(3), 458-463. | No placebo group |
| 1. Da Silva, I. B., Rafaela Damasceno, S., Maco, D. P. C., & Randau, K. P. (2018). Use of medicinal plants in the treatment of erysipelas: A review. Pharmacognosy Reviews, 12(24), 200-207. https://doi.org/10.4103/phrev.phrev_5_18 | No placebo group |
| 1. Dubreuil, C. (2001). The new fluoroquinones in the treatment of acute bacterial sinusitis. Medecine et Maladies Infectieuses, 31(SUPPL. 5), 657-659. https://doi.org/10.1016/S0399-077X(01)80110-4 | No placebo group |
| 1. Elliott, S. A., Gaudet, L. A., Fernandes, R. M., Vandermeer, B., Freedman, S. B., Johnson, D. W., Plint, A. C., Klassen, T. P., Allain, D., & Hartling, L. (2021). Comparative Efficacy of Bronchiolitis Interventions in Acute Care: A Network Meta-analysis. Pediatrics, 147(5), 05. https://doi.org/https://dx.doi.org/10.1542/peds.2020-040816 | No placebo group |
| 1. Fahimi, J., Singh, A., & Frazee, B. (2012). Antibiotics for the treatment of abscesses: A meta-analysis. Academic Emergency Medicine, 19, S177-S178. https://doi.org/10.1111/j.1553-2712.2012.01332.x | No placebo group |
| 1. Farrell, K., Tandan, M., Hernandez Santiago, V., Gagyor, I., Braend, A. M., Skow, M., Vik, I., Jansaaker, F., Hayward, G., & Vellinga, A. (2021). Treatment of uncomplicated UTI in males: a systematic review of the literature. Bjgp Open, 5(2). https://doi.org/https://dx.doi.org/10.3399/bjgpopen20X101140 | No placebo group |
| 1. Gao, L. N., Lyu, J., Wang, Z. F., Yu, D. D., & Sun, M. H. (2019). [Meta-analysis of randomized controlled trials on effect of Tanreqing Injection combined with Western medicine on acute exacerbation of chronic bronchitis]. Zhongguo Zhong Yao Za Zhi/Zhongguo Zhongyao Zazhi/China Journal of Chinese Materia Medica, 44(24), 5313-5321. https://doi.org/https://dx.doi.org/10.19540/j.cnki.cjcmm.20190924.501 | No placebo group |
| 1. George, S. M. C., Karanovic, S., Harrison, D. A., Rani, A., Birnie, A. J., Bath‐Hextall, F. J., Ravenscroft, J. C., & Williams, H. C. (2019). Interventions to reduce Staphylococcus aureus in the management of eczema. Cochrane Database of Systematic Reviews(10). https://doi.org/10.1002/14651858.CD003871.pub3 | No placebo group |
| 1. Goodlet, K. J., Benhalima, F. Z., & Nailor, M. D. (2019). A systematic review of single-dose aminoglycoside therapy for urinary tract infection: Is it time to resurrect an old strategy? Antimicrobial Agents and Chemotherapy, 63(1). https://doi.org/10.1128/AAC.02165-18 | No placebo group |
| 1. Guo, C., Sun, X., Wang, X., Guo, Q., & Chen, D. (2018). Network Meta-Analysis Comparing the Efficacy of Therapeutic Treatments for Bronchiolitis in Children. Jpen: Journal of Parenteral & Enteral Nutrition, 42(1), 186-195. https://doi.org/https://dx.doi.org/10.1002/jpen.1030 | No placebo group |
| 1. Guo, W., Zhu, C., Stevens, G., & Silverstein, D. (2021). Analyzing the Efficacy of Isotretinoin in Treating Dissecting Cellulitis: A Literature Review and Meta-Analysis. Drugs in R and D, 21(1), 29-37. https://doi.org/10.1007/s40268-020-00335-y | No placebo group |
| 1. Lee, H., Kang, B., Hong, M., Lee, H. L., Choi, J. Y., & Lee, J. A. (2020). Eunkyosan for the common cold: A PRISMA-compliment systematic review of randomised, controlled trials. Medicine, 99(31), e21415. https://doi.org/10.1097/MD.0000000000021415 | No placebo group |
| 1. Li, G., Cai, L., Jiang, H., Dong, S., Fan, T., Liu, W., Xie, L., & Mao, B. (2015). Compound Formulas of Traditional Chinese Medicine for the Common Cold: Systematic Review of Randomized, Placebo-controlled Trials. Alternative therapies in health and medicine, 21(6), 48-57. https://www.embase.com/search/results?subaction=viewrecord&id=L608957362&from=export | No placebo group |
| 1. Li, L., Xie, Y., Chai, Y., Liao, X., & Pei, W. (2016). Qingkailing injection for uncomplicated upper respiratory tract infection: A systematic review and meta-analysis. European Journal of Integrative Medicine, 8(5), 609-618. https://doi.org/10.1016/j.eujim.2016.06.019 | No placebo group |
| 1. Li, S., Yue, J., Dong, B. R., Yang, M., Lin, X., & Wu, T. (2013). Acetaminophen (paracetamol) for the common cold in adults. Cochrane Database of Systematic Reviews(7). https://doi.org/10.1002/14651858.CD008800.pub2 | No placebo group |
| 1. Lin, H. S., Lin, P. T., Tsai, Y. S., Wang, S. H., & Chi, C. C. (2021). Interventions for bacterial folliculitis and boils (furuncles and carbuncles). Cochrane Database of Systematic Reviews(2). https://doi.org/10.1002/14651858.CD013099.pub2 | No placebo group |
| 1. Liu, K. X., Xu, B., Wang, J., Zhang, J., Ding, H., Ariani, F., Qu, J. M., & Lin, Q. C. (2014). Efficacy and safety of moxifloxacin in acute exacerbations of chronic bronchitis and COPD: A systematic review and meta-analysis. Journal of Thoracic Disease, 6(3), 221-229. https://doi.org/10.3978/j.issn.2072-1439.2013.11.12 | No placebo group |
| 1. Lin, C., & Pang, Q. (2018). Meta-analysis and systematic review of procalcitonin-guided treatment in acute exacerbation of chronic obstructive pulmonary disease [Review]. Clinical Respiratory Journal, 12(1), 10-15. https://doi.org/10.1111/crj.12519 | No placebo group |
| 1. Lyu, J., Xie, Y., Sun, M., Zhang, C., & Wang, L. (2020). Sanjin tablet combined with antibiotics for treating patients with acute lower urinary tract infections: A meta-analysis and GRADE evidence profile. Experimental and Therapeutic Medicine, 19(1), 683-695. https://doi.org/10.3892/etm.2019.8252 | No placebo group |
| 1. Marom, T., Marchisio, P., Tamir, S. O., Torretta, S., Gavriel, H., & Esposito, S. (2016). Complementary and Alternative Medicine Treatment Options for Otitis Media: A Systematic Review. Medicine, 95(6), e2695. https://doi.org/https://dx.doi.org/10.1097/MD.0000000000002695 | No placebo group |
| 1. Moraa, I., Sturman, N., McGuire, T. M., & van Driel, M. L. (2021). Heliox for croup in children. Cochrane Database of Systematic Reviews(8). https://doi.org/10.1002/14651858.CD006822.pub6 | No placebo group |
| 1. Ong Lopez, A. M. C., Tan, C. J. L., Yabon, A. S., & Masbang, A. N. (2021). Symptomatic treatment (using NSAIDS) versus antibiotics in uncomplicated lower urinary tract infection: a meta-analysis and systematic review of randomized controlled trials. BMC Infectious Diseases, 21(1). https://doi.org/10.1186/s12879-021-06323-0 | No placebo group |
| 1. Panpanich, R., Lerttrakarnnon, P., & Laopaiboon, M. (2004). Azithromycin for acute lower respiratory tract infections. Cochrane Database of Systematic Reviews(4), CD001954. | No placebo group |
| 1. Porreca, A., D'Agostino, D., Romagnoli, D., Del Giudice, F., Maggi, M., Palmer, K., Falabella, R., De Berardinis, E., Sciarra, A., Ferro, M., Artibani, W., Mirone, V., & Busetto, G. M. (2021). The Clinical Efficacy of Nitrofurantoin for Treating Uncomplicated Urinary Tract Infection in Adults: A Systematic Review of Randomized Control Trials. Urologia Internationalis, 105(7), 531-540. https://doi.org/10.1159/000512582 | No placebo group |
| 1. Ran, L., Zhao, W., Wang, H., Zhao, Y., & Bu, H. (2020). Vitamin C as a Supplementary Therapy in Relieving Symptoms of the Common Cold: A Meta-Analysis of 10 Randomized Controlled Trials. BioMed Research International, 2020, 8573742. https://doi.org/https://dx.doi.org/10.1155/2020/8573742 | No placebo group |
| 1. Ranakusuma, R. W., Pitoyo, Y., Safitri, E. D., Thorning, S., Beller, E. M., Sastroasmoro, S., & Del Mar, C. B. (2018). Systemic corticosteroids for acute otitis media in children. Cochrane Database of Systematic Reviews(3). https://doi.org/10.1002/14651858.CD012289.pub2 | No placebo group |
| 1. Son, M. J., Kim, Y. E., Song, Y. I., & Kim, Y. H. (2017). Herbal medicines for treating acute otitis media: A systematic review of randomised controlled trials. Complementary Therapies in Medicine, 35, 133-139. https://doi.org/10.1016/j.ctim.2017.11.001 | No placebo group |
| 1. van Driel, M. L., De Sutter, A. I., Thorning, S., & Christiaens, T. (2021). Different antibiotic treatments for group A streptococcal pharyngitis. Cochrane Database of Systematic Reviews, 3, CD004406. https://doi.org/https://dx.doi.org/10.1002/14651858.CD004406.pub5 | No placebo group |
| 1. Vorwerk, C., & Coats, T. (2010). Heliox for croup in children. Cochrane database of systematic reviews (Online), 2, CD006822. | No placebo group |
| 1. Walters, J. A. E., Tan, D. J., White, C. J., & Wood‐Baker, R. (2018). Different durations of corticosteroid therapy for exacerbations of chronic obstructive pulmonary disease. Cochrane Database of Systematic Reviews(3). https://doi.org/10.1002/14651858.CD006897.pub4 | No placebo group |
| 1. Wang, S. Z., Hu, J. T., Zhang, C., Zhou, W., Chen, X. F., Jiang, L. Y., & Tang, Z. H. (2014). The safety and efficacy of daptomycin versus other antibiotics for skin and soft-tissue infections: a meta-analysis of randomised controlled trials. BMJ Open, 4(6), e004744. https://doi.org/https://dx.doi.org/10.1136/bmjopen-2013-004744 | No placebo group |
| 1. Wang, T., Wu, G., Wang, J., Cui, Y., Ma, J., Zhu, Z., Qiu, J., & Wu, J. (2020). Comparison of single-dose fosfomycin tromethamine and other antibiotics for lower uncomplicated urinary tract infection in women and asymptomatic bacteriuria in pregnant women: A systematic review and meta-analysis. International Journal of Antimicrobial Agents, 56(1), 106018. https://doi.org/https://dx.doi.org/10.1016/j.ijantimicag.2020.106018 | No placebo group |
| 1. Wei, J., Ni, J., Wu, T., Chen, X., Duan, X., Liu, G., Yiao, J., Wang, Q., Zhen, J., & Zhou, L. (2006). A systematic review of Chinese medicinal herbs for acute bronchitis. Journal of Alternative & Complementary Medicine, 12(2), 159-169. | No placebo group |
| 1. Wu, T., Chen, X., Duan, X., Juan, N., Liu, G., Qiao, J., Wang, Q., Wei, J., Zhen, J., & Zhou, L. (2005). Chinese medicinal herbs for acute bronchitis. Cochrane database of systematic reviews (Online)(3), CD004560. | No placebo group |
| 1. Xia, R. Y., Hu, X. Y., Fei, Y. T., Willcox, M., Wen, L. Z., Yu, M. K., Zhang, L. S., Dai, M. Y., Fei, G. H., Thomas, M., Francis, N., Wilkinson, T., Moore, M., & Liu, J. P. (2020). Shufeng jiedu capsules for treating acute exacerbations of chronic obstructive pulmonary disease: A systematic review and meta-analysis. BMC Complementary Medicine and Therapies, 20(1). https://doi.org/10.1186/s12906-020-02924-5 | No placebo group |
| 1. Zalmanovici Trestioreanu, A., Green, H., Paul, M., Yaphe, J., & Leibovici, L. (2010). Antimicrobial agents for treating uncomplicated urinary tract infection in women. Cochrane Database of Systematic Reviews(10). https://doi.org/10.1002/14651858.CD007182.pub2 | No placebo group |
| 1. Zhang, L., Wang, R., Falagas, M. E., Chen, L. A., & Liu, Y. N. (2012). Gemifloxacin for the treatment of community-acquired pneumonia and acute exacerbation of chronic bronchitis: a meta-analysis of randomized controlled trials. Chinese Medical Journal, 125(4), 687-695. | No placebo group |
| 1. Zhang, W. B., Jiang, H. L., Zhou, W., Zhong, Y. Q., Yang, H. M., Fu, J. J., & Mao, B. (2009). Chinese medicine for acute upper respiratory tract infection: A systematic review of randomized controlled trials. Journal of Chinese Integrative Medicine, 7(8), 706-716. https://doi.org/10.3736/jcim20090802 | No placebo group |
| 1. Zhang, Y. Y., Xia, R. Y., Liang, S. B., Hu, X. Y., Dai, M. Y., Li, Y. L., Zhao, L. Y., Moore, M., Fei, Y. T., & Liu, J. P. (2021). Chinese patent herbal medicine (Shufeng Jiedu capsule) for acute upper respiratory tract infections: A systematic review and meta-analysis. Integrative Medicine Research, 10(3). https://doi.org/10.1016/j.imr.2021.100726 | No placebo group |
| 1. Acute sinusitis. (2006). MeReC Bulletin, 17(3), 6-8. | Study not found |
|  |  |

**Additional Table 3:** List of excluded primary studies

| **Primary studies** | **Reason** |
| --- | --- |
| 1. Bjornson, C. L., Klassen, T. P., Williamson, J., Brant, R., Mitton, C., Plint, A., Bulloch, B., Evered, L., & Johnson, D. W. (2004). A randomized trial of a single dose of oral dexamethasone for mild croup. *New England Journal of Medicine*, *351*(13), 1306-1313. https://doi.org/10.1056/NEJMoa033534 | Ineligible population |
| 1. Geelhoed, G. C., Turner, J., & Macdonald, W. B. (1996). Efficacy of a small single dose of oral dexamethasone for outpatient croup: a double blind placebo controlled clinical trial. *BMJ*, *313*(7050), 140-142. | Ineligible population |
| 1. Johnson, D. W., Schuh, S., Koren, G., & Jaffe, D. M. (1996). Outpatient treatment of croup with nebulized dexamethasone. *Archives of Pediatrics and Adolescent Medicine*, *150*(4), 349-355. https://doi.org/10.1001/archpedi.1996.02170290015002 | Ineligible population |
| 1. Klassen, T. P., Feldman, M. E., Watters, L. K., Sutcliffe, T., & Rowe, P. C. (1994). Nebulized budesonide for children with mild-to-moderate croup. *New England Journal of Medicine*, *331*(5), 285-289. | Ineligible population |
| 1. Kristjansson, S., Berg-Kelly, K., & Winso, E. (1994). Inhalation of racemic adrenaline in the treatment of mild and moderately severe croup. Clinical symptom score and oxygen saturation measurements for evaluation of treatment effects. *Acta Paediatrica*, *83*(11), 1156-1160. | Ineligible population |
| 1. Duong, M., Markwell, S., Peter, J., & Barenkamp, S. (2010). Randomized, controlled trial of antibiotics in the management of community-acquired skin abscesses in the pediatric patient. *Annals of Emergency Medicine*, *55*(5), 401-407. https://doi.org/https://dx.doi.org/10.1016/j.annemergmed.2009.03.014 | Ineligible population |
| 1. Lopez, J., Gomez, G., Rodriguez, K., Davila, J., Nunez, J., & Anaya, L. (2018). Comparative Study of Drainage and Antibiotics versus Drainage Only in the Management of Primary Subcutaneous Abscesses. *Surgical Infections*, *19*(3), 345-351. https://doi.org/https://dx.doi.org/10.1089/sur.2017.225 | Ineligible population |
| 1. Rajendran, P. M., Young, D., Maurer, T., Chambers, H., Perdreau-Remington, F., Ro, P., & Harris, H. (2007). Randomized, double-blind, placebo-controlled trial of cephalexin for treatment of uncomplicated skin abscesses in a population at risk for community-acquired methicillin-resistant Staphylococcus aureus infection. *Antimicrobial Agents & Chemotherapy*, *51*(11), 4044-4048. | Ineligible population |
| 1. Management of erysipela and necrotizing faciitis. (2000). Annales de Dermatologie et de Venereologie, 127(12), 1118-1137. | Ineligible population |
| 1. Bacterial skin infections: impetigo, furunculosis, erysipelas. Etiology, diagnosis, clinical course, treatment. (2000). Annales de Dermatologie et de Venereologie, 127(Spec No 1), A98-103. | Ineligible design |
| 1. Abrantes, M. A., & Lavareda, C. (1997). [A patient with erysipela, how to treat him?]. *Servir*, *45*(3), 148-156. | Ineligible design |
| 1. Anonymous. (1887). The Prognosis of Croup. *The Southern Medical Record*, *17*(1), 14-15. | Ineligible population |
| 1. Aramburo, A. (2003). Carbuncle: An old disease for a new era. *Acta Pediatrica Espanola*, *61*(1), 2-8. | Ineligible design |
| 1. Barkhatova, N. A. (2009). [Detoxication and anti-inflammatory therapy in the treatment of generalized soft tissue infections]. *Khirurgiia*(5), 27-30. | Ineligible design |
| 1. Bonnetblanc, J. M. (1990). [Erysipelas, lymphangitis. Etiology, diagnosis, clinical course and prognosis, treatment]. *Revue du Praticien*, *40*(21), 2005-2006. | Ineligible design |
| 1. Chen, M. N., Shen, Y. Z., Huang, Z. X., & Zhu, J. (2015). The curative observation and nursing care of cold compress with Si-huang Honeyed Powder in the treatment of acute skin lesions that suffered from erysipelas. *Journal of emergency in traditional chinese medicine [zhong guo zhong yi ji zheng]*, *24*(4), 678‐680. | Ineligible design |
| 1. Crickx, B. (1993). [Bacterial cutaneous infections: erysipelas. Etiology, diagnosis, clinical course amd treatment]. *Revue du Praticien*, *43*(7), 876-878. | Ineligible design |
| 1. Fitzgerald, D., Mellis, C., Johnson, M., Allen, H., Cooper, P., & Van Asperen, P. (1996). Nebulized budesonide is as effective as nebulized adrenaline in moderately severe croup. *Pediatrics*, *97*(5), 722-725. | Ineligible population |
| 1. Fleisher, G. (1980). Pediatric skin, soft tissue, and bone infections. *Delaware Medical Journal*, *52*(11), 587-592. | Ineligible design |
| 1. Galbiati, G., Righini, V., Renzetti, D., & Bevilacqua, M. (2002). Double-blind clinical study of a topical preparation versus placebo in women with edematofibrous panniculopathy localized in the thighs. *Giornale Italiano di Dermatologia e Venereologia*, *137*(3), 217-224 | Ineligible design |
| 1. Geelhoed, G. (2003). Acute upper airways obstruction in children. *Medicine Today*, *4*(3), 43-46. | Ineligible design |
| 1. Ieki, R. (1999). [Croup]. *Ryoikibetsu Shokogun Shirizu*(24 Pt 2), 80-82 | Ineligible population |
| 1. Irct20190914044765N. (2019). Comparison the effect of oral and intravenous dexamethasone effect on the mild and moderate Croup treatment in children. | Ineligible population |
| 1. Irct20210913052453N. (2022). Treatment of Upper Respiratory Infection. *https://trialsearch.who.int/Trial2.aspx?TrialID=IRCT20210913052453N2*. https://www.cochranelibrary.com/central/doi/10.1002/central/CN-02429649/full | Ineligible population |
| 1. Isrctn. (2000). Pragmatic primary care randomised placebo controlled trial of steroids (oral or nebulised) in the treatment of croup. | Ineligible population |
| 1. Jaussaud, R., Kaeppler, E., Strady, C., Beguinot, I., Waldner, A., & Remy, G. (2001). [Should NSAID/corticoids be considered when treating erysipelas?]. *Annales de Dermatologie et de Venereologie*, *128*(3 Pt 2), 348-351 | Ineligible design |
| 1. Kavlakov, P., & Atanasov, D. (1977). [Incidence, location, clinical course and therapy of abscesses and cellulitis in the maxillofacial region]. *Stomatologiia*, *59*(5), 332-337. | Ineligible population |
| 1. Klassen, T. P., Watters, L. K., Feldman, M. E., Sutcliffe, T., & Rowe, P. C. (1996). The efficacy of nebulized budesonide in dexamethasone-treated outpatients with croup. *Pediatrics*, *97*(4), 463-466. | Ineligible population |
| 1. Kristjansson, S., Berg-Kelly, K., & Winso, E. (1994). Inhalation of racemic adrenaline in the treatment of mild and moderately severe croup. Clinical symptom score and oxygen saturation measurements for evaluation of treatment effects. *Acta Paediatrica*, *83*(11), 1156-1160. | Ineligible population |
| 1. Kriukova, S. A. (1980). [Prognostic importance of determining the nonspecific resistance and allergy indices in erysipelas]. *Vrachebnoe Delo*(4), 107-109. | Ineligible design |
| 1. Kriukova, S. A., & Panichkina, L. N. (1985). [Prognostic value of studying indices of nonspecific resistance and humoral immunity in patients with erysipelas]. Laboratornoe Delo(9), 555-556. | Ineligible design |
| 1. Kriukova, Z. V., Panichkina, L. N., & Kriukova, S. A. (1984). [Characteristics of the clinical course of erysipelas and various indicators of nonspecific resistance in middle-aged patients]. Vestnik Dermatologii i Venerologii(8), 48-50. | Ineligible design |
| 1. Kurokawa, I., & Nishijima, S. (1999). [Carbuncle]. Ryoikibetsu Shokogun Shirizu(23 Pt 1), 574-575. | Ineligible outcome |
| 1. List, M., Headlee, D., & Kondratuk, K. (2016). Treatment of Skin Abscesses: A Review of Wound Packing and Post-Procedural Antibiotics. South Dakota Medicine: The Journal of the South Dakota State Medical Association, 69(3), 113-119. | Ineligible population |
| 1. Lopatina, Z. M. (1961). Certain characteristics of the clinical course of recurrent erysipelas. Sovetskaia meditsina, 25, 81-87. | Ineligible outcome |
| 1. Meyer-Rohn, J. (1974). [Bacterial diseases of the skin]. Hautarzt, 25(6), 277-283. | Ineligible design |
| 1. Moon, K. T. (2011). Drainage sufficient treatment for smaller Uncomplicated Abscesses. American Family Physician, 83(8). | Ineligible population |
| 1. Nct. (2005). Placebo Controlled Study of Antibiotic Treatment of Soft Tissue Infection. *https://clinicaltrials.gov/show/NCT00187759*. https://www.cochranelibrary.com/central/doi/10.1002/central/CN-01585588/full | Ineligible outcome |
| 1. Nct. (2006). An Attempt to Reduce Community-Acquired Methicillin-Resistant Staphylococcus Aureus (MRSA) Infection in Soldiers. *https://clinicaltrials.gov/show/NCT00289588*. https://www.cochranelibrary.com/central/doi/10.1002/central/CN-01480962/full | Ineligible outcome |
| 1. Nct. (2007). The Natural History of Community-Associated MRSA Infections and Decolonization Strategies. *https://clinicaltrials.gov/show/NCT00513799*. https://www.cochranelibrary.com/central/doi/10.1002/central/CN-02028901/full | Ineligible outcome |
| 1. Nct. (2008). Strategies Using Off-Patent Antibiotics for Methicillin Resistant S. Aureus "STOP MRSA". *https://clinicaltrials.gov/show/NCT00729937*. https://www.cochranelibrary.com/central/doi/10.1002/central/CN-02027209/full | Ineligible outcome |
| 1. Nct. (2009). Trial of Antibiotic Treatment for Skin Abscess in Patients at Risk for Methicillin-Resistant Staphylococcus Aureus (MRSA) Infection. *https://clinicaltrials.gov/show/NCT00829686*. https://www.cochranelibrary.com/central/doi/10.1002/central/CN-01523790/full | Study not found |
| 1. Nct. (2012). Evaluation of a Staphylococcus Eradication Protocol for Patients Who Present to the ED With Cutaneous Abscess. *https://clinicaltrials.gov/show/NCT01537783*. https://www.cochranelibrary.com/central/doi/10.1002/central/CN-01535712/full | Study not found |
| 1. Nct. (2013). A Study Assessing the Impact of Dermatology Consultation in Patients Presenting With Possible Cellulitis. *https://clinicaltrials.gov/show/NCT01795092*. https://www.cochranelibrary.com/central/doi/10.1002/central/CN-02021651/full | Study not found |
| 1. Nct. (2014). US Guided Versus BlindI&D for Treatment of Soft Tissue Abscesses in the ED. *https://clinicaltrials.gov/show/NCT02264392*. https://www.cochranelibrary.com/central/doi/10.1002/central/CN-02040060/full | Study not found |
| 1. Neefjes, C. P. (1974). [Pseudocroup]. *Nederlands Tijdschrift voor Geneeskunde*, *118*(31), 1181-1185. | Ineligible population |
| 1. Okamoto, O., Suzuki, R., Kusatsu, M., Matsuda, K., Sato, S., Kai, Y., Shiota, S., & Hashimoto, H. (2018). A Statistical Study about Clinical and Laboratory Trends in Cellulitis/Erysiperas. *Nishinihon Journal of Dermatology*, *80*(3), 231-238. https://doi.org/10.2336/nishinihonhifu.80.231 | No placebo |
| 1. Postovit, V. A., & Mel'k, M. V. (1981). [Characteristics of the clinical course of erysipelas in the middle-aged and elderly]. *Sovetskaia meditsina*(3), 100-104. | Ineligible outcome |
| 1. Rajendran, P. M., Young, D., Maurer, T., Chambers, H., Perdreau-Remington, F., Ro, P., & Harris, H. (2007). Randomized, double-blind, placebo-controlled trial of cephalexin for treatment of uncomplicated skin abscesses in a population at risk for community-acquired methicillin-resistant Staphylococcus aureus infection. *Antimicrobial Agents & Chemotherapy*, *51*(11), 4044-4048. | Ineligible population |
| 1. Rustamov Ya, A., Iskulov, F. S., Markova, A. V., & Dvoriakova, Z. I. (1975). The clinical course and treatment of croup syndrome in children (Russian). *Zdravookhranenie Tadzhikistana*, *No.1*, 34-36. | Ineligible population |
| 1. Slawson, J. G. (1996). Efficacy of oral dexamethasone for outpatient croup. *Journal of Family Practice*, *43*(5), 436. | Ineligible population |
| 1. Suleimenova, Z. I., Turkpenbaeva, G., Tsoi, I. G., & Turkpenbaeva, A. (1985). [Characteristics of the clinical course of erysipelas]. *Klinicheskaia Meditsina*, *63*(1), 125-128. | No placebo |
| 1. Sumboonnanonda, A., Suwanjutha, S., & Sirinavin, S. (1997). Randomized controlled trial of dexamethasone in infectious croup. *Journal of the Medical Association of Thailand*, *80*(4), 262-265. | Ineligible population |
| 1. Tarasov, V. I., Zinchuk, N. V., & Dergacheva, S. I. (1977). [Morbidity and clinical course of erysipelas in adults]. *Vrachebnoe Delo*(4), 135-137. | Ineligible outcome |
| 1. Ellis, M. W., Griffith, M. E., Dooley, D. P., McLean, J. C., Jorgensen, J. H., Patterson, J. E., Davis, K. A., Hawley, J. S., Regules, J. A., Rivard, R. G., & et al. (2007). Targeted intranasal mupirocin to prevent colonization and infection by community-associated methicillin-resistant Staphylococcus aureus strains in soldiers: a cluster randomized controlled trial. *Antimicrobial agents and chemotherapy*, *51*(10), 3591‐3598. https://doi.org/10.1128/AAC.01086-06 | Ineligible outcome |
| 1. Lee, G. C., Dallas, S. D., Winkler, P., Du, L. C., Trevino, L., Trevino, S., Pena, J., Mann, M., Munoz, A., Marcos, Y., Rocha, G., Koretsky, S., Esparza, S., Finnie, M., & Frei, C. R. (2014). Prospective comparison of clinical outcomes of community-associated methicillin-resistant staphylococcus aureus (Ca-Mrsa) and methicillin-susceptible staphylococcus aureus (Ca-Mssa) skin and soft tissue infections (Sstis): A starnet study. *Value in Health*, *17*(3), A268. https://doi.org/10.1016/j.jval.2014.03.1560 | Ineligible outcome |
| 1. Geelhoed, G. C., & Macdonald, W. B. (1995). Oral and inhaled steroids in croup: a randomized, placebo-controlled trial. *Pediatric Pulmonology*, *20*(6), 355-361. | Ineligible population |
| 1. Johnson, D. W., Schuh, S., Koren, G., & Jaffe, D. M. (1996). Outpatient treatment of croup with nebulized dexamethasone. *Archives of Pediatrics and Adolescent Medicine*, *150*(4), 349-355. https://doi.org/10.1001/archpedi.1996.02170290015002 | Ineligible population |
| 1. Neto, G. M., Kentab, O., Klassen, T. P., & Osmond, M. H. (2002). A randomized controlled trial of mist in the acute treatment of moderate croup. *Academic Emergency Medicine*, *9*(9), 873-879. | Ineligible population |
| 1. Custer, J. R. (1993). Croup and related disorders. *Pediatrics in Review*, *14*(1), 19-29. | Ineligible population |
| 1. Willman, M. (2016). Factors associated with guideline discordant antibiotic prescribing for emergency department cutaneous abscesses. *Academic Emergency Medicine*, *23*, S232-S233. https://doi.org/10.1111/acem.12974 | Ineligible population |
| 1. Pallin, D. J., Allen, M. B., Binder, W. D., Filbin, M. R., Parmar, S., Hooper, D. C., & Camargo, C. A. (2012). Randomized, double-blind, placebo-controlled trial of trimethoprim-sulfamethoxazole added to cephalexin for treatment of cellulitis in the age of CA-MRSA. *Annals of Emergency Medicine*, *60*(4), S45-S46. https://doi.org/10.1016/j.annemergmed.2012.06.102 | No placebo |
| 1. Cutrera, R., Baraldi, E., Indinnimeo, L., Miraglia Del Giudice, M., Piacentini, G., Scaglione, F., Ullmann, N., Moschino, L., Galdo, F., & Duse, M. (2017). Management of acute respiratory diseases in the pediatric population: the role of oral corticosteroids. *Italian Journal of Pediatrics*, *43*(1), 31. https://doi.org/https://dx.doi.org/10.1186/s13052-017-0348-x | Ineligible population |
| 1. Li, D. G., Di Xia, F., Khosravi, H., Dewan, A. K., Pallin, D. J., Baugh, C. W., Laskowski, K., Joyce, C., & Mostaghimi, A. (2018). Outcomes of early dermatology consultation for inpatients diagnosed with cellulitis. *JAMA Dermatology*, *154*(5), 537-543. https://doi.org/10.1001/jamadermatol.2017.6197 | No placebo |
| 1. Bjornson, C. L., Klassen, T. P., Williamson, J., Brant, R., Mitton, C., Plint, A., Bulloch, B., Evered, L., & Johnson, D. W. (2004). A randomized trial of a single dose of oral dexamethasone for mild croup. *New England Journal of Medicine*, *351*(13), 1306-1313. https://doi.org/10.1056/NEJMoa033534 | Ineligible population |
| 1. Chinnock, B., & Hendey, G. W. (2016). Irrigation of Cutaneous Abscesses Does Not Improve Treatment Success. *Annals of Emergency Medicine*, *67*(3), 379-383. https://doi.org/https://dx.doi.org/10.1016/j.annemergmed.2015.08.007 | Ineligible population |
| 1. Roorda, R. J., & Walhof, C. M. (1998). Effects of inhaled fluticasone propionate administered with metered dose inhaler and spacer in mild to moderate croup: a negative preliminary report. *Pediatric Pulmonology*, *25*(2), 114-117. | Ineligible population |
| 1. Olderog, C. K., Schmitz, G. R., Bruner, D. R., Pittoti, R., Williams, J., & Ouyang, K. (2012). Clinical and epidemiologic characteristics as predictors of treatment failures in uncomplicated skin abscesses within seven days after incision and drainage. *Journal of Emergency Medicine*, *43*(4), 605-611. https://doi.org/https://dx.doi.org/10.1016/j.jemermed.2011.09.037 | Ineligible population |
| 1. Dobrovoljac, M., & Geelhoed, G. C. (2012). How fast does oral dexamethasone work in mild to moderately severe croup? A randomized double-blinded clinical trial. *Emergency Medicine Australasia*, *24*(1), 79-85. https://doi.org/https://dx.doi.org/10.1111/j.1742-6723.2011.01475.x | Ineligible population |
| 1. Geelhoed, G. C. (2005). Budesonide offers no advantage when added to oral dexamethasone in the treatment of croup. *Pediatric Emergency Care*, *21*(6), 359-362 | Ineligible population |
| 1. Adelman, A. (1996). Treatment of croup with nebulized dexamethasone. *Journal of Family Practice*, *43*(1), 19-20. | Ineligible population |
| 1. Cates, J. E., Mitrani-Gold, F. S., Li, G., & Mundy, L. M. (2015). Systematic Review and Meta-Analysis To Estimate Antibacterial Treatment Effect in Acute Bacterial Skin and Skin Structure Infection. *Antimicrobial Agents & Chemotherapy*, *59*(8), 4510-4520. https://doi.org/https://dx.doi.org/10.1128/AAC.00679-15 | Ineligile design |
| 1. Nishat, M., Latif, A., Chaudhry, N., Ansar, A., Choudry, Z. A. L. I., Butt, M. Q., & Shabbir, F. (2018). Management of carbuncle; prognosis of surgical treatment. *Pakistan Journal of Medical and Health Sciences*, *12*(2), 637-639. | Ineligible outcome |
| 1. Taussig, L. M., Castro, O., Beaudry, P. H., & Fox, W. W. (1975). Treatment of laryngotracheobronchitis (croup). Use of intermittent positive pressure breathing and racemic epinephrine. *American journal of diseases of children (1960)*, *129*(7), 790‐793. https://www.cochranelibrary.com/central/doi/10.1002/central/CN-00341598/full | Ineligible population |
| 1. Arakaki, R. Y., Strazzula, L., Woo, E., & Kroshinsky, D. (2014). The impact of dermatology consultation on diagnostic accuracy and antibiotic use among patients with suspected cellulitis seen at outpatient internal medicine offices: a randomized clinical trial. *JAMA Dermatology*, *150*(10), 1056‐1061. https://doi.org/10.1001/jamadermatol.2014.1085 | No placebo |
| 1. Gaspari, R. J., Resop, D., Mendoza, M., Kang, T., & Blehar, D. (2011). A randomized controlled trial of incision and drainage versus ultrasonographically guided needle aspiration for skin abscesses and the effect of methicillin-resistant Staphylococcus aureus. *Annals of Emergency Medicine*, *57*(5), 483-491.e481. https://doi.org/https://dx.doi.org/10.1016/j.annemergmed.2010.11.021 | Ineligible population |
| 1. Geelhoed, G. C., & Macdonald, W. B. (1995). Oral dexamethasone in the treatment of croup: 0.15 mg/kg versus 0.3 mg/kg versus 0.6 mg/kg. *Pediatric Pulmonology*, *20*(6), 362-368. | Ineligible population |
| 1. Knutson, D., & Aring, A. (2004). Viral croup. *American Family Physician*, *69*(3), 535-540. | Ineligible population |
| 1. Lin, H. S., Lin, P. T., Tsai, Y. S., Wang, S. H., & Chi, C. C. (2021). Interventions for bacterial folliculitis and boils (furuncles and carbuncles). *Cochrane Database of Systematic Reviews*, *2*, CD013099. https://doi.org/https://dx.doi.org/10.1002/14651858.CD013099.pub2 | Ineligible design |
| 1. Klassen, T. P., Craig, W. R., Moher, D., Osmond, M. H., Pasterkamp, H., Sutcliffe, T., Watters, L. K., & Rowe, P. C. (1998). Nebulized budesonide and oral dexamethasone for treatment of croup: a randomized controlled trial. *JAMA*, *279*(20), 1629-1632 | Ineligible population |
| 1. Donaldson, D., Poleski, D., Knipple, E., Filips, K., Reetz, L., Pascual, R. G., & Jackson, R. E. (2003). Intramuscular versus oral dexamethasone for the treatment of moderate-to-severe croup: a randomized, double-blind trial. *Academic Emergency Medicine*, *10*(1), 16-21. | Ineligible population |
| 1. Luria, J. W., Gonzalez-del-Rey, J. A., DiGiulio, G. A., McAneney, C. M., Olson, J. J., & Ruddy, R. M. (2001). Effectiveness of oral or nebulized dexamethasone for children with mild croup. *Archives of Pediatrics & Adolescent Medicine*, *155*(12), 1340-1345. | Ineligible population |
| 1. Lopez, J., Gomez, G., Rodriguez, K., Davila, J., Nunez, J., & Anaya, L. (2018). Comparative Study of Drainage and Antibiotics versus Drainage Only in the Management of Primary Subcutaneous Abscesses. *Surgical Infections*, *19*(3), 345-351. https://doi.org/https://dx.doi.org/10.1089/sur.2017.225 | Ineligible population |
| 1. Brindle, R. (2016). The natural history of antibiotic-treated limb cellulitis: Data derived from a randomised trial of cellulitis of over 400 patients allows the determination of time to recovery and clarifies the utility of systemic observations, blood tests, pain scores, and local limb measurements. *Open Forum Infectious Diseases*, *3*. https://doi.org/10.1093/ofid/ofw172.862 | No placebo |
| 1. Klassen, T. P., Feldman, M. E., Watters, L. K., Sutcliffe, T., & Rowe, P. C. (1994). Nebulized budesonide for children with mild-to-moderate croup. *New England Journal of Medicine*, *331*(5), 285-289. | Ineligible population |
| 1. Garbutt, J. M., Conlon, B., Sterkel, R., Baty, J., Schechtman, K. B., Mandrell, K., Leege, E., Gentry, S., & Stunk, R. C. (2013). The comparative effectiveness of prednisolone and dexamethasone for children with croup: a community-based randomized trial. *Clinical Pediatrics*, *52*(11), 1014-1021. https://doi.org/https://dx.doi.org/10.1177/0009922813504823 | Ineligible population |
| 1. Pallin, D. J., Binder, W. D., Allen, M. B., Lederman, M., Parmar, S., Filbin, M. R., Hooper, D. C., & Camargo, C. A., Jr. (2013). Clinical trial: comparative effectiveness of cephalexin plus trimethoprim-sulfamethoxazole versus cephalexin alone for treatment of uncomplicated cellulitis: a randomized controlled trial. *Clinical Infectious Diseases*, *56*(12), 1754-1762. https://doi.org/https://dx.doi.org/10.1093/cid/cit122 | No placebo |
| 1. Asmundsson, A. S., Arms, J., Kaila, R., Roback, M. G., Theiler, C., Davey, C. S., & Louie, J. P. (2019). Hospital Course of Croup After Emergency Department Management. *Hospital Pediatrics*, *9*(5), 326-332. https://doi.org/https://dx.doi.org/10.1542/hpeds.2018-0066 | Ineligible population |
| 1. Schmitz, G. R., Pitotti, R., Olderog, C., Livengood, T., & Williams, J. (2009). Prospective randomized trial of trimethoprim-sulfamethoxazole vs placebo on 30-day recurrence rates for uncomplicated skin abscesses in patients at risk for community-acquired methicillin-resistant staphylococcus aureus infection: An interim analysis. *Annals of Emergency Medicine*, *54*(3), S104. | Ineligible population |
| 1. Fahimi, J., Singh, A., & Frazee, B. (2012). Antibiotics for the treatment of abscesses: A meta-analysis. *Academic Emergency Medicine*, *19*, S177-S178. https://doi.org/10.1111/j.1553-2712.2012.01332.x | Ineligible design |
| 1. Gaspari, R., Blehar, D., Mendoza, M., Montoya, A., Moon, C., & Polan, D. (2009). Use of ultrasound elastography for skin and subcutaneous abscesses. *Journal of Ultrasound in Medicine*, *28*(7), 855-860. | Ineligible population |
| 1. Fifoot, A. A., & Ting, J. Y. (2007). Comparison between single-dose oral prednisolone and oral dexamethasone in the treatment of croup: a randomized, double-blinded clinical trial. *Emergency Medicine Australasia*, *19*(1), 51-58. | Ineligible population |
| 1. Cetinkaya, F., Tufekci, B. S., & Kutluk, G. (2004). A comparison of nebulized budesonide, and intramuscular, and oral dexamethasone for treatment of croup. *International Journal of Pediatric Otorhinolaryngology*, *68*(4), 453-456. | Ineligible population |
| 1. Haran, J. P., Goulding, M., Campion, M., Scully, G., Chandra, A., Goldberg, R., Day, A., McLendon, E., & Clark, M. A. (2020). Reduction of Inappropriate Antibiotic Use and Improved Outcomes by Implementation of an Algorithm-Based Clinical Guideline for Nonpurulent Skin and Soft Tissue Infections. *Annals of Emergency Medicine*, *76*(1), 56-66. https://doi.org/https://dx.doi.org/10.1016/j.annemergmed.2019.12.012 | Ineligible design |
| 1. Duong, M., Markwell, S., Peter, J., & Barenkamp, S. (2010). Randomized, controlled trial of antibiotics in the management of community-acquired skin abscesses in the pediatric patient. *Annals of Emergency Medicine*, *55*(5), 401-407. https://doi.org/https://dx.doi.org/10.1016/j.annemergmed.2009.03.014 | Ineligible population |
| 1. Geelhoed, G. C., Turner, J., & Macdonald, W. B. (1996). Efficacy of a small single dose of oral dexamethasone for outpatient croup: a double blind placebo controlled clinical trial. *BMJ*, *313*(7050), 140-142. | Ineligible population |
| 1. Glenn, I. C., Bruns, N. E., Soldes, O. S., & Ponsky, T. A. (2018). Prospective observational study to assess the need for postoperative antibiotics following surgical incision and drainage of skin and soft tissue abscess in pediatric patients. *Journal of Pediatric Surgery*, *53*(8), 1469-1471. https://doi.org/https://dx.doi.org/10.1016/j.jpedsurg.2017.08.004 | Ineligible population |
| 1. Li, D., Xia, F., Khosravi, H., Dewan, A., Pallin, D., Baugh, C., Laskowski, K., & Mostaghimi, A. (2018). Impact of dermatology intervention on cellulitis management: A prospective study. *Journal of the American Academy of Dermatology*, *79*(3), AB158. https://doi.org/10.1016/j.jaad.2018.05.650 | No placebo |
| 1. Vorwerk, C., & Coats, T. J. (2008). Use of helium-oxygen mixtures in the treatment of croup: a systematic review. *Emergency Medicine Journal*, *25*(9), 547-550. https://doi.org/https://dx.doi.org/10.1136/emj.2007.052878 | Ineligible population |
| 1. Shah, N., Buice, J., Shields, A., & Pruitt, C. (2020). Does Point-of-Care Ultrasound Affect Outcomes in Pediatric Patients with Skin and Soft Tissue Infections? *Southern Medical Journal*, *113*(12), 645-650. https://doi.org/https://dx.doi.org/10.14423/SMJ.0000000000001185 | Ineligible outcome |
| 1. Lee, G. C., Hall, R. G., Boyd, N. K., Dallas, S. D., Du, L. C., Trevino, L. B., Trevino, S. B., Retzloff, C., Lawson, K. A., Wilson, J., Olsen, R. J., Wang, Y., & Frei, C. R. (2016). A prospective observational cohort study in primary care practices to identify factors associated with treatment failure in Staphylococcus aureus skin and soft tissue infections. *Annals of Clinical Microbiology & Antimicrobials*, *15*(1), 58. | Ineligible outcome |
| 1. Mangione-Smith, R., Zhou, C., Williams, D. J., Johnson, D. P., Kenyon, C. C., Tyler, A., Quinonez, R., Vachani, J., McGalliard, J., Tieder, J. S., Simon, T. D., Wilson, K. M., & Pediatric Research in Inpatient Settings, N. (2019). Pediatric Respiratory Illness Measurement System (PRIMES) Scores and Outcomes. *Pediatrics*, *144*(2), 08. https://doi.org/https://dx.doi.org/10.1542/peds.2019-0242 | Ineligible outcome |
| 1. May, L. S., Zocchi, M., Zatorski, C., Jordan, J. A., Rothman, R. E., Ware, C. E., Eells, S., & Miller, L. (2015). Treatment Failure Outcomes for Emergency Department Patients with Skin and Soft Tissue Infections. *The Western Journal of Emergency Medicine*, *16*(5), 642-652. https://doi.org/https://dx.doi.org/10.5811/westjem.2015.7.26213 | Ineligible outcome |
| 1. Tercero Talavera, F. I., & Rapkin, R. H. (1974). Antibiotic usage in the management of acute laryngotracheobronchitis (croup). *Clinical Pediatrics*, *13*(12), 1074-1076. | Ineligible population |
| 1. Lee, J. H., Jung, J. Y., Lee, H. J., Kim, D. K., Kwak, Y. H., Chang, I., Kwon, H., Choi, Y. J., Park, J. W., Paek, S. H., & Cho, J. H. (2019). Efficacy of low-dose nebulized epinephrine as treatment for croup: A randomized, placebo-controlled, double-blind trial. *American Journal of Emergency Medicine*, *37*(12), 2171-2176. https://doi.org/https://dx.doi.org/10.1016/j.ajem.2019.03.012 | Ineligle population |
| 1. Ledwith, C. A., Shea, L. M., & Mauro, R. D. (1995). Safety and efficacy of nebulized racemic epinephrine in conjunction with oral dexamethasone and mist in the outpatient treatment of croup. *Annals of Emergency Medicine*, *25*(3), 331-337. | Ineligible population |
| 1. Qureshi, A. Z. (2012). The three musketeers: cellulitis, phlegmon and abscess. *JPMA - Journal of the Pakistan Medical Association*, *62*(11), 1258. | No placebo |
| 1. Ausejo, M., Saenz, A., Pham, B., Kellner, J. D., Johnson, D. W., Moher, D., & Klassen, T. P. (1999). The effectiveness of glucocorticoids in treating croup: meta-analysis. *BMJ*, *319*(7210), 595-600. | Ineligible population |
| 1. Garlicki, A. M., Jawien, M., Pancewicz, S., Moniuszko-Malinowska, A., Kalinowska-Nowak, A., & Bociaga-Jasik, M. (2020). Management of bacterial skin and soft tissue infections. *Przeglad Epidemiologiczny*, *74*(1), 89-107. https://doi.org/https://dx.doi.org/10.32394/pe.74.07 | No placebo |
| 1. Gibbons, J. A., Smith, H. L., Kumar, S. C., Duggins, K. J., Bushman, A. M., Danielson, J. M., Yost, W. J., & Wadle, J. J. (2017). Antimicrobial stewardship in the treatment of skin and soft tissue infections. *American Journal of Infection Control*, *45*(11), 1203-1207. https://doi.org/https://dx.doi.org/10.1016/j.ajic.2017.05.013 | Ineligible design |
| 1. Ellis, M. W., Hospenthal, D. R., Dooley, D. P., Gray, P. J., & Murray, C. K. (2004). Natural history of community-acquired methicillin-resistant Staphylococcus aureus colonization and infection in soldiers. *Clinical Infectious Diseases*, *39*(7), 971-979. | No placebo |
| 1. Huang, M. N., Chang, Y. C., Wu, C. H., Hsieh, S. C., & Yu, C. L. (2009). The prognostic values of soft tissue sonography for adult cellulitis without pus or abscess formation. *Internal Medicine Journal*, *39*(12), 841-844. | No placebo |

**Additional Table 4:** Characteristics of included systematic reviews.

| **Study ID** | **Year of review’s last search** | **No. of studies included (sample size)^1^** | | **Participants** | | | **Intervention** | **Comparator** | **Outcomes of relevance to natural history^2^** | | |
| --- | --- | --- | --- | --- | --- | --- | --- | --- | --- | --- | --- |
|  |  | **RCTs** | **Cohort** | **Age (years)** | **Inclusion criteria** | **Exclusion criteria** |  |  | **Mean duration of symptoms** | **Proportion of participants with symptom resolution at specified timepoint/s** |  |
| **Acute respiratory infections** | | | | | | | | | | | |
| **Acute bronchitis (Cough)** | | | | | | | | | | | |
| Becker 2015 | 2015 | 7 (522) | - | ≥ 2 years | Clinical diagnosis of acute bronchitis or acute cough | - Pre-existing pulmonary diseases, such as asthma, COPD (emphysema or chronic bronchitis, or cystic fibrosis. - Sinusitis, pertussis, or pneumonia | Beta2-agonists (oral or inhaled) | Placebo, alternative treatment, or no treatment | - | ✔ |  |
| Bergmann 2021 | 2020 | 1 (1503) | 6(9964) | Any age | Cough as a reason for encounters in primary care. | - Qualitative studies, case reports, studies from secondary and tertiary care | Antibiotics, complementary therapy | Placebo | ✔ | ✔ |  |
| Ebell 2013 | 2011 | 18 (4810) | 1(3402) | >18 years | Undifferentiated acute cough, studies of acute bronchitis | - chronic respiratory illness (e.g., pneumonia, sinusitis, anthrax, or influenza). - tuberculosis, vaccinations, asthma, allergies, anthrax, chronic lung disease, or cystic fibrosis, Rhinitis, postinfectious or chronic cough | NR | Placebo | ✔ | ✔ |  |
| Fahey 1996 | NR | 9 (939) | - | >12 years | acute cough with or without purulent sputum, not treated in the preceding week with antibiotics. | - chronic obstructive airway disease. | Antibiotics | placebo or no treatment | ✔ | ✔ |  |
| Hay 2002 | 1998 | 8 (1777) | 2 (1055) | <4 years | cough or undifferentiated acute respiratory tract infection with cough (UARTIC) | - tuberculosis, pertussis, bronchiolitis, cystic fibrosis, and recurrent wheezing. | Cough mixtures, decongestants, analgesics, ephedrine ‘Routine care for fever’ | Placebo | ✔ | ✔ |  |
| Smith 2017 | 2017 | 11 (5099) | - | Any age | cough with or without productive sputum Trials that allowed concurrent use of other medications, such as analgesics, antitussives, antipyretics, or mucolytics, in both groups | - pre-existing chronic bronchitis (i.e., acute exacerbation of chronic bronchitis). | Antibiotics | Placebo | ✔ | ✔ |  |
| Speich 2018 | 2017 | 6 (724) | - | ≥16 years | A cough of 3–8 weeks (subacute) | - known chronic respiratory diseases example, GORD, chronic obstructive pulmonary disease [COPD], or asthma) | Oral montelukast, gelatine, inhaled salbutamol plus ipratropium bromide, fluticasone | Placebo or usual care | ✔ | ✔ |  |
| Wagner 2015 | 2012 | 34 (7083) | - | Any age | cough as a symptom of URTI or a common cold. | - Other chronic diseases. - Studies where Phyto therapeutics were not the main intervention. | Combination of herbal preparations | Placebo, no intervention, and other agents | - | ✔ |  |
| **Common cold** | | | | | | | | | | | |
| De Sutter 2015 | 2015 | 18 (4342) | - | Any age | Common cold symptoms of recent onset of symptoms of runny and/or stuffy nose; and sneezing with or without symptoms of headache and cough | - allergic rhinitis. - chronic lower respiratory tract infections, such as pneumonia, bronchitis, bronchiolitis, chronic disease, atopic eczema, asthma, fever (> 38 °C), sinusitis, or exudative pharyngitis; | Antihistamines (either sedating or non-sedating) | Placebo or no treatment | - | ✔ |  |
| De Sutter 2022 | 2021 | 30 (6304) | - | ≥ 6 months | common cold symptoms comprising runny and/or stuffy nose and sneezing, with or without symptoms of headache and cough | - allergic rhinitis, other chronic diseases, atopic eczema, asthma, fever (> 38 °C), sinusitis, exudative pharyngitis, or had symptoms for over a week. | Combination therapies containing analgesics and/ or decongestants and/or antihistamines. | Active intervention, placebo, or no treatment | - | ✔ |  |
| Deckx 2016 | 2016 | 11 (1838) | - | Any age | Common cold, characterised by defined symptoms of an upper respiratory tract infection (URTI). | - influenza, sinusitis, or rhinitis) | Nasal and oral decongestants combinations | Placebo | ✔ | - |  |
| Douglas 2004 | 2004 | 21(11, 077) | - | Any age | trials of vitamin C to prevent or treat the common cold using oral doses of vitamin C of 200 mg/day or more. | - Dose less than 200 mg | orally administered vitamin C | Placebo | ✔ | - |  |
| Hayward 2015 | 2015 | 3 (353) | - | Any age | common cold, defined by clinical diagnosis. Trials that allowed concurrent use of other medications if they permitted equal access for patients in both groups, | - influenza or sinusitis. - experimentally induced common cold | Oral or inhaled corticosteroids | Standard care or placebo. | ✔ | - |  |
| Karsch‐Völk 2014 | 2013 | 24 (4631) | - | Any age | clinical diagnosis of common cold, influenza-like syndrome, or viral URTI | - Trials with no placebo arm - Experimentally induced colds | Echinacea mono-preparations | placebo | ✔ | - |  |
| Kenealy 2013 | 2015 | 11 (1047) | - | Any age | symptoms of acute upper respiratory tract infection (common cold). Trials allowing concurrent use of other medications if they allowed equal access for participants in both the antibiotic and placebo group | - Studies involving the use of active substances, if more than 5% of participants had swabs positive for beta haemolytic streptococcal infection, history of serious illness | Antibiotics | Placebo | - | ✔ |  |
| Kim 2015 | 2015 | 9 (1069) | - | Any age | symptoms of a common cold. Trials that allowed concurrent use of other medications if they permitted equal access for patients in treatment and placebo groups | - allergic rhinitis, lower or chronic respiratory infection or another chronic disease, atopic eczema, asthma, fever (> 38 °C), sinusitis or exudative pharyngitis | NSAIDS | placebo | ✔ | - |  |
| Lissiman 2014 | 2014 | 1 (146) | - | ≤17 years | common cold or non-specific viral upper respiratory tract infection (URTI). | - Influenza, myalgia, and fever greater than 38 °C. | Garlic (Allium sativum) | placebo, no treatment, or standard treatment | ✔ | - |  |
| Science 2012 | 2011 | 17 (2021) | - | Any age | common cold symptoms | - studies in which zinc was administered intranasally or that used zinc in a combined formulation with other minerals or vitamins | Zinc oral | Placebo | ✔ | ✔ |  |
| **Sore throat** |  |  |  |  |  |  |  |  |  |  |  |
| de Cassan 2020 | 2019 | 9 (1319) | - | > 3 years | sore throat symptoms | - hospitalised participants - glandular fever, sore throat following tonsillectomy or intubation, or peritonsillar abscess. | Steroid | Placebo/ standard care | ✔ | ✔ |  |
| Spinks 2021 | 2021 | 29 (15337) | - | Any age | Randomised controlled trials (RCTs) or quasi-RCTs | NR | Antibiotics | Placebo | - | ✔ |  |
| **Sinusitis** | | | | | | | | | | | |
| Lemiengre 2018 | 2018 | 15(3057) | - | Adults | upper respiratory tract infection or common cold if most participants had rhinosinusitis-like symptoms. | - if more than 50% of participants were considered to have a common cold, - signs, and symptoms for more than 30 days. | Antibiotics | Placebo or no treatment | ✔ | ✔ |  |
| Shaikh 2014 | Jun | 0^3^ | NR | ≤18 years | acute sinusitis, defined as 10 to 30 days of rhinorrhoea, congestion or daytime cough. | - Trials involving surgery or sinus puncture, chronic sinusitis (symptoms for more than 30 days), allergic rhinitis, or URTIs. | Antihistamine and decongestants | Placebo | - | - |  |
| Venekamp 2014 | 2014 | 5 (1133) | - | > 15 years | acute sinusitis | - corticosteroids delivered by the intranasal route or by inhalation | Corticosteroids (oral) | Placebo in four trials and NSAIDs in one trial | ✔ | ✔ |  |
| Zalmanovici 2013 | 2013 | 4 (1943) | - | Any age | acute sinusitis defined by clinical diagnosis and confirmed by radiological evidence or by nasal endoscopy | - Non-randomised; observational studies | Intranasal corticosteroids | no intervention or control group | ✔ | ✔ |  |
| **Otitis media with effusion** | | | | | | | | | | | |
| Griffin 2011 | 2011 | 16 (1880) | - | < 18 years | diagnosis of Otitis media with effusion | - acute otitis media, anatomical deformity, chronic immunocompromised states. | Antihistamine, decongestant, antihistamine/decongestant combination, steroids | Placebo or no treatment | - | ✔ |  |
| Venekamp 2016 | 2016 | 25 (3258) | - | ≤ 18 years | diagnosis (tympanometry alone or in combination with otoscopy) of unilateral or bilateral OME. | - ventilation tubes present, chronic suppurative otitis media, known immunodeficiency, Down syndrome, or craniofacial anomalies, including cleft palate. | Oral antibiotics | placebo, no treatment, or therapy of unproven effectiveness. | ✔ | ✔ |  |
| **Otitis media** | | | | | | | | | | | |
| Venekamp 2015 | 2015 | 13 (3401) | - | > one month to 15 years | RCTs of antimicrobial drugs versus placebo control. RCTs comparing immediate antibiotic versus expectant observation. | NR | Antibiotics, Immediate antibiotic versus expectant observation | Placebo | - | ✔ |  |
| **Otitis externa** | | | | | | | | | | | |
| Kaushik 2010 | 2009 | 19 (3382) |  | Any age | Otitis externa with intact tympanic membranes | - complicated acute otitis externa, otitis externa secondary to otitis media or chronic suppurative otitis media; chronic otitis, externa; fungal otitis externa (otomycosis); eczematous otitis externa; viral otitis externa and furunculosis | Topical or systemic medication including steroids and antibiotics | Ear cleaning or placebo |  | ✔ |  |
| Rosenfeld 2006 | 2006 | 20 (3289) |  | Any age | Studies of acute otitis externa | - Tympanic membrane perforation, non-intact tympanic membrane |  |  |  | ✔ |  |
| **Bronchiolitis** | | | | | | | | | | | |
| Gadomski 2014 | 2014 | 30 (1992) | - | ≤ 24 months | Symptoms of bronchiolitis | - Studies of inhaled steroids | Bronchodilator therapy | Placebo | ✔ | ✔ |  |
| **Acute exacerbation of Chronic Obstructive Pulmonary Diseases** | | | | | | | | | | | |
| Vollenweider 2012 | 2018 | 19 (2663) | - | >4 year | clinical (physician-based) diagnosis of COPD or, spirometrically confirmed. | - acute bronchitis, pneumonia, asthma, or bronchiectasis.  Studies that used antibiotics for the prevention of exacerbations. | Oral antibiotics | Placebo | ✔ | ✔ |  |
| **Laryngitis** |  |  |  |  |  |  |  |  |  |  |  |
| Reveiz 2015 | 2014 | 3 (351) | - | Adults | acute laryngitis defined by the International Classification of Health Problems in Primary Care. | - chronic underlying diseases, symptoms of laryngitis for more than three weeks (chronic laryngitis), and those receiving antibiotic therapy within the two weeks preceding diagnosis | Antibiotics | Placebo | - | ✔ |  |
| **Rhinitis** |  |  |  |  |  |  |  |  |  |  |  |
| Segboaer 2019 | 2019 | 13 (2045) | - | ≥ 12 years | non-allergic rhinitis. | - Perioperative studies, allergic rhinitis, infectious rhinitis; acute or chronic rhinosinusitis; auto-immune rhinitis; rhinitis related to anatomical abnormalities. | Any intranasal corticosteroid | Placebo (inactive vehicle of the intervention medication) | - | ✔ |  |
| **Conjunctivitis** | | | | | | | | | | | |
| Sheikh 2012 | 2022 | 11 (3673) | - | ≥ one month | RCTs) of antibiotic treatment compared with placebo/vehicle | NR | antibiotic | ‘Vehicle-control, antiseptic in some trails | ✔ | ✔ |  |
| **Multiple respiratory tract conditions** | | | | | | | | | | | |
| Rosenfeld 2003 | 2002 | 14 (2900) | 27 (10,063) | ≤ 18 years. | Studies of acute otitis media and OME. | NR | symptomatic therapy, including analgesics, nose drops, antihistamines, decongestant | Placebo or no treatment | ✔ | ✔ |  |
| Thompson 2013 | 2012 | 28 (3038) | 20 (5336) | ≤ 18 years | ARI in primary care or emergency settings, with earache (acute otitis media), sore throat (or pharyngitis or tonsillitis), cough (or acute bronchitis, bronchiolitis, or croup), and common cold (or upper respiratory tract infection) | - Chronic, recurrent, or complicated infections. - Experimentally induced infection, cystic fibrosis, immunodeficiency). | Symptomatic treatment or antibiotic | Placebo | ✔ | ✔ |  |
| **Urinary tract infections** | | | | | | | | | | | |
| Uncomplicated Urinary tract infection | | | | | | | | | | | |
| Hoffmann 2020 | 2019 | 3 (346) | - | Adults | Uncomplicated UTI | - asymptomatic UTIs; asymptomatic bacteriuria; complicated UTI (for pyelonephritis and sepsis); recurrent UTI; chronic UTI, emphysematous cystitis; Candida infection; haemorrhagic cystitis; interstitial cystitis; or schistosomiasis. | NR | No treatment or placebo | ✔ | ✔ |  |
| **Skin and soft tissue infections** | | | | | | | | | | | |
| **Abscess** | | | | | | | | | | | |
| Bowen 2017 | 2017 | 10 (4346) | 5 (48940) | Any age | RCTs, non-RCTs, and observational studies.  Any literature reporting susceptibility of GAS to SXT or TMP | NR | sulfamethoxazole-trimethoprim | placebo or other antibiotic | - | ✔ |  |
| Wang 2018 | 2017 | 14 (3541) | - | Any age | RCTs comparing antibiotics to no antibiotics | - Patients with diabetes, trial with only one event | Antibiotics | placebo | - | ✔ |  |
| **Impetigo** | | | | | | | | | | | |
| Hoffmann 2021 | 2020 | 7 (557) | - | Any age | Participants with impetigo in either the placebo group of randomised trials or in single-group prognostic studies that did not use antibiotics and measured time to resolution or improvement | NR | - | placebo including topical creams ointments, and an oral, povidone-iodine | ✔ | ✔ |  |
|  |  |  |  |  |  |  |  |  |  |  |  |

RCT: Randomised placebo-controlled study. OME: Otitis media with effusion

NR: Not reported.

^1^ Unless otherwise stated, this is the number of all studies included in the original review irrespective of their contribution to the outcome of interest.

^2^ Only the outcomes relevant to the scoping review objectives reported. Other outcomes are reported in the original reviews.

**Additional Table 5:** Natural history information, per condition (45^1^), as reported in the included 40 systematic reviews.

| **Study ID** | **Number of studies providing NH data**  **n/N** | | **Duration of illness before recruitment** | **Follow-up (data collection points in days)** | **x/n** | **Mean duration of symptom resolution^2^** | **y/n** | **Proportion of participants who are symptom-free, improved, clinically cured (at each time point)^3^ %** |  |  |
| --- | --- | --- | --- | --- | --- | --- | --- | --- | --- | --- |
|  | |  | | | **Systematic reviews with a focus on natural history** | | | | | |
| **Cough** |  | |  |  |  |  |  |  |  |  |
| Ebell 2013 | 13/19 | | NR | Ranged from 7 days to 2 months: (8, 16, 17, 21, 24) | 5/13 | Mean duration of any cough: 17.8 days (range = 15.3 to 28.6 days), [ as reported, pooled analysis, 1821 participants] | 3/13 | Clinically cured. Day 8: 20% to 73% [as reported, narratively 404 participants] |  | |
|  |  | |  |  | 2/13 | Daytime cough: 12.7 [as reported, pooled analysis, 172 participants] | 1/13 | ^4^Day 16: 9% [calculated, narrative report, in-text data 46 participants] |  | |
|  |  | |  |  | 2/13 | Nighttime cough: 10.4 [ as reported, pooled analysis, 153 participants] | 1/13 | ^4^Day 17: 27% [calculated, narrative report, in-text data, 104 participants] |  | |
|  |  | |  |  | 3/13 | Productive cough: 13.9 [as reported, pooled analysis, 153 participants | 1/13 | ^4^Day 21: 18% [calculated, narrative report, in-text data, 63 participants] |  | |
| Hay 2002 | 10/10 | | < 4 days and up to 2 weeks | range = 2 to 28 days (1-2, 5-8, 20-21 days0 | 1/10 | Mean recovery time: 5.8 days, 9.1 with no complications [as reported, narratively report, 68 participants] | 2/10 | Improved/ resolved.  Day 2: 66% [as reported, narratively, 74 participants] |  | |
|  |  | |  |  | 1/10 | Mean illness duration: 3.6 days [as reported, narratively, 303 participants] | 4/10 | Day 7: 75% [as reported, narratively, 169 participants] |  | |
|  |  | |  |  |  |  | 4/10 | **^5^**Day 14: 76% [calculated, narrative report, in-text data, unclear number of participants] |  | |
| ^6^Thompson 2013 | 5/5 | | less than 14 days | Follow-up 14-21 days or until symptoms resolved | 5/5 | Mean duration 1-25 days [as reported narratively, 828 participants] | 5/5 | Symptoms resolved.  Day 10: 50% [as reported, pooled analysis, 828 participants] |  |  |
|  |  | |  |  |  |  | 5/5 | Day 25: 90% [as reported, pooled analysis, 828 participants] |  |  |
| **Sore throat** |  | |  |  |  |  |  |  |  |  |
| ^6^Thompson 2013 | 7/7 | | < 1 to 4 days | 2-7 days or until symptom resolution | 4/7 | Mean duration:  2-6.7 days [as reported, narratively, 235 participants] | 1/7 | Proportion without symptoms  ^7^Day 1: 47% [calculated from summary tables, 17 participants] |  |  |
|  |  | |  |  |  |  | 1/7 | Day 2: 65% [calculated, narrative report, 17 participants] |  |  |
|  |  | |  |  |  |  | 2/7 | Day 3: 63-67% [calculated, narrative report, in-text data 206 participants] |  |  |
| **Otitis media** | | | | | | | | | | |
| ^8^Rosenfeld 2003 | 11/21 | | NR | NR (1,2-3, 4-7 days) | 6/11 | Mean duration  7-14 days [ as reported, pooled analysis, 542 participants] | 3/11 | Improved  Day 1: 61% [as reported, pooled analysis, 315 participants] |  |  |
|  |  | |  |  |  |  | 5/11 | Day 2-3: 80% [as reported, pooled analysis, 808 participants] |  |  |
|  |  | |  |  |  |  | 8/11 | Day 4-7: 74% [as reported, pooled analysis, 712 participants. |  |  |
|  |  | |  |  |  |  | 6/11 | Complete clinical resolution  Day 7-14: 70% [as reported, pooled analysis, 542 participants] |  |  |
| ^6^Thompson 2013 | 10/10 | | <4 days | Ranged from 8 days to 3 months or until symptoms resolution | 5/10 | 0.5-9 days [as reported, narratively, 500 participants] | 10/10 | Symptoms resolved.  Day 3: 50% [as reported, pooled analysis, 1376 participants] |  |  |
|  |  | |  |  |  |  | 10/10 | Day 7-8: 90% [as reported, pooled analysis, 1376 participants] |  |  |
| **Otitis Media with Effusion** | | | | | | | | |  |  |
| ^8^Rosenfeld 2003 | 20/20 | | Entry rate of infection provided | NR [1,3,6,9 months] | - | NR | 4/20 | Resolved  OME [untreated OM]  1 month: 59% [as reported, narratively, unclear number of participants] |  |  |
|  |  | |  |  |  |  | 4/20 | 3 months: 74% [as reported narratively, unclear number of participants |  |  |
|  |  | |  |  |  |  | 4/20 | OME of unknown duration  3 months:22-28% [as reported, pooled analysis, 291 participants. |  |  |
|  |  | |  |  |  |  | 4/20 | 6 months: 42% [as reported, pooled analysis, 229 participants |  |  |
|  |  | |  |  |  |  | 4/20 | 9 months: 56% [ as reported, pooled analysis, 133 participants] |  |  |
|  |  | |  |  |  |  |  |  |  |  |
| **Common cold** | | | | | | | | | | |
| ^6^Thompson 2013 | 6/6 | | one day  to 8.7 (SD 5.1) days. | 2 to 21 days in RCTs, 2-3 weeks in observational studies | 4/6 | Mean 7-15 days [as reported narratively, 1768 participants] | 5/6 | improved.  Day 10: 50% [as reported, pooled analysis, 565 participants] |  |  |
|  |  | |  |  |  |  | 6/6 | Day 15: 90% [as reported, narrative report, 712 participants] |  |  |
| **Croup** |  | |  |  |  |  |  |  |  |  |
| ^6^Thompson 2013 | 3/3 | | 21 hours to 2.4 days | 7-21 days | 2/3 | 2-3 days [as reported narratively, 67 participants] | 2/3 | Resolved  Day 1: 50% [as reported, pooled analysis, 415 participants] |  |  |
|  |  | |  |  |  |  | 2/3 | Day 2: 80% [as reported, pooled analysis, 415 participants] |  |  |
| **Bronchiolitis** | | | | | | | | | | |
| ^6^Thompson 2013 | 4/4 | | 4 days | 2-3 weeks | 2/4 | Mean duration  8-15 days [as reported, narratively, 156 participants] | 4/4 | Improved.  Day 13: 50% [as reported, pooled analysis, 520 participants] |  |  |
|  |  | |  |  |  |  | 4/4 | ^9^Day 21: 90% [as reported, narratively, 520 participants] |  |  |
| **Urinary tract infections** | | | | | | | | | | |
| ^10^Hoffmann 2020 | 3/3 | | NR  . | 7days to 6 weeks [3,4,7,9 days, 6 weeks] | 3/3 | Mean duration  9 days [as reported, narratively report, 111 participants] | 1/3 | **Symptom free**  Day 9: 42% [as reported, narratively, 78 participants. |  |  |
|  |  | |  |  |  |  | 1/3 | Week 6: 54% [as reported, narratively, 166 participants] |  |  |
|  |  | |  |  |  |  | 1/3 | **Symptoms improved.**  Day 3: 34% [as reported narratively, 35 participants] |  |  |
|  |  | |  |  |  |  | 1/3 | Day 7: 9% [as reported narratively, 33 participants] |  |  |
| **Impetigo** | | | | | | | | | | |
| ^11^Hoffmann 2021 | 7/7 | | 6-8 days | 0-14 | 7/7 | Mean duration  7 days [ reported pooled result, 557 participants] | 7/7 | Cured.  Day 7: 13%-74% [as reported, narratively, 577 participants] |  |  |
|  | |  | | | **Systematic reviews with placebo-controlled RCTs** | | | | | |
| Cough | | | | | | | | | | |
| ^12^Becker 2015 | 1/7 | | < 4 weeks | 1 to 7 days (7) | - | - | 1/1 | improved or cured Day 7: 59% [calculated pooled analysis, 29 participants] |  |  |
|  |  | |  |  |  |  |  |  |  |  |
| Bergmann 2021 | 4/7 | | less than 7 days | 28 days (3,7,14,28). | 1/4 | Mean illness duration: 20.4 (SD10) [ as reported pooled analysis, 2530 participants] | 1/4 | Improved Day 3: 52% [as reported, narratively, 1016 participants] |  |  |
|  |  | |  |  | 1/4 | The median duration of cough: 8 (IQR 6–14.5) days. | 1/4 | Day 7: 65.7% [as reported, narratively, 1016 participants] |  |  |
|  |  | |  |  | 1/4 | Median time for recovery: 9 to 11 days. | 1/4 | Day 14: 81% [as reported, narratively, 1016 participants] |  |  |
|  |  | |  |  |  |  | 1/4  2/4  1/4 | **Cured**  Day 7:10.8% [as reported, narratively, 1016 participants]  Day 14: 40-67% [as reported, narratively, 3706 participants]  Day 28+ 79% [as reported, narratively, 3100 participants] |  |  |
| Fahey 1998 | 9/9 | | NR | 7-11 days | 5/9 | Duration of resolution:  7-11 days [as reported narratively, 344 participants] | 5/9 | Proportion improved Day 7-11: 76% [calculated, pooled analysis, summary table, 252 participants] |  |  |
| ^13^Speich 2018 | 6/6 | | 3–8 weeks (that is, subacute) | 14 and 28 days | 1/6 | - | 1/6 | Improved  Day 14: 54% [as reported, narratively, 43 participants] |  |  |
| ^14^Wagner 2015 | 4/34 | | 2 days | 3 to 10 days | - | NR | 3/4 | Improve or cure (Ivy, Primrose, and Thyme) Day 7-9: 55% [calculated, pooled analysis, 395 participants] |  |  |
| **Sore throat** |  | |  |  |  |  |  |  |  |  |
| ^15^de Cassan 2020 | 1/9 | | < 7 days | 1,3,7 and 28 days | 1/1 | Median duration of pain:  2.3. [ as reported narratively, 60 participants] | 1/1 | Complete resolution  Day 1: 18% [calculated, narrative report, in-text data, 277 participants] |  |  |
|  |  | |  |  |  |  | 1.1 | Day 2: 27% [calculated, narrative report, in-text data, 277 participants] |  |  |
| ^16^Spinks 2021 | 29/29 | | 6 to 8 days | Day 3 and 7 | - | NR | 16/29 | Proportion with symptom of sore throat on day 3 [ reversed for those without symptoms] Day 3: 33.7% [calculated, pooled analysis 1607 participants] |  |  |
|  |  | |  |  |  |  | 14/29 | Day 7: 82% [as reported narratively,1187 participants] |  |  |
| **Sinusitis** |  | |  |  |  |  |  |  |  |  |
| Lemiengre 2018 | 15/15 | | 4 to 15.4 | NR | 3/15 | ^17^Mean duration: 3.7 to 7 days [as reported narratively, 253 participants] | 5/15 | Cured participants  Day 7: 46% [as reported, pooled analysis, 503 participants] |  |  |
|  |  | |  |  | 2/15 | Median duration: 13.5 and 17 [as reported narratively, 65 participants] | 11/15 | Day 10: 53% [as reported, pooled analysis, 603 participants] |  |  |
|  |  | |  |  |  |  | 4/15 | Day 14: 67.5% [as reported, pooled analysis, 351 participants] |  |  |
| ^18^Shaikh 2014 | NA | | NA | NA |  | NA |  | NA |  |  |
| Venekamp 2014 | 1/5 | | Median=13.5 days | 0 and day 14 of treatment. End of follow-up (8 weeks) | 1/1 | Time to resolution: 9 days [as reported narratively, 86 participants] | 1/1 | Resolution or improved symptoms Day 7: 55.8% [as reported, narratively, 86 participants] |  |  |
|  |  | |  |  |  |  | 1/1 | Day 14: 80% [as reported narratively, 86 participants] |  |  |
| ^19^Zalmanovici 2013 | 4/4 | | NR | 15-21 days | 3/4 | Median number of days to clinical success: 9.5 days [as reported, narratively] | 3/4 | Resolution of symptoms or improved  Day 9.5: 66.4% [calculated; pooled analysis, 635 participants] |  |  |
| **Common Cold** | | | | | | | | | | |
| ^20^De Sutter 2015 | 18/18 | | <48 hours | 6-10 days | - | NR | 3/18 | Improved/cured.  Days 1-2: 63% [calculated, pooled analysis, 750 participants] |  |  |
|  |  | |  |  |  |  | 1/18 | Day 3-4: 70.5% [calculated, pooled analysis, 115 participants |  |  |
|  |  | |  |  |  |  | 3/18 | Day 6-10: 30% [calculated, pooled analysis, 778 participants] |  |  |
| De Sutter 2022 | 14/30 | | <48 hours to 7 days. | Up to 14 days. | - | NR | 6/14 | Global evaluation [cure]  Antihistamine-decongestant  Day 6.5: 55.3% [as reported. pooled analysis, 284 participants |  |  |
|  |  | |  |  |  |  | 1/4 | Antihistamine-Analgesic  Day 6: 43% [as reported, pooled analysis, 292 participants |  |  |
|  |  | |  |  |  |  | 1/7 | Analgesic Decongestant Day 5: 52 [as reported pooled result, 90 participants] |  |  |
|  |  | |  |  |  |  | 1/6 | Antihistamine-analgesic-decongestant Day 1: 65.8% [calculated, pooled analysis, 266 participants |  |  |
|  |  | |  |  |  |  | 1/6 | Day 3: 93.7% [calculated, pooled analysis, 48 participants] |  |  |
|  |  | |  |  |  |  | 1/6 | Day 5: 45% [calculated, pooled analysis, 40 participants] |  |  |
| Deckx 2016 | 1/15 | | Range from 28 hours to less than five days | one to 10 days | 1/15 | Mean duration  6 days [as reported narratively, 123 participants] | - | NR |  |  |
| Douglas 2004 | 11/21 | | NR | 4 days after day 0 of treatment | 1/11 | Duration of colds: 3.52 days [as reported narratively, 285 participants]. |  | NR |  |  |
| ^21^Hayward 2015 | 3/3 | | 24 hours to 3 days | 1, 7, 14, days after treatment and 1 month | 1/3 | Mean number of symptomatic days 10.3 days [as reported narratively, 54 participants]  3. Median time to recovery 11 days. | 1/3 | NR |  |  |
| ^22^Karsch‐Völk 2014 | 5/24 | | up to 36 hours | 24 hours to 10 days | 2/5 | Mean duration of cold:  5.75 (SD 2.34) [calculated, pooled analysis 73 participants] 6.86 (SD 3.62) [calculated, pooled analysis, 176 participants]  Pooled mean: 6.5 [3.2] |  | NR |  |  |
| ^23^Kenealy 2013 | 6/11 | | less than 10 days | 1 to 7 days | - | NR | 5/6 | Persisting symptoms [reversed for those without symptoms]  Day: 1-7: 65% [ calculated, pooled analysis, 461 participants] |  |  |
| Kim 2015 | 6/11 | | 2 days or less | Follow-up period 7 days | 2/6 | Mean duration of colds 8.4 (SD 3.4) [as reported, pooled analysis 214 participants 8.4 (SD 3.4), [as reported, pooled analysis, 90 participants] 3 (SD 2) [as reported, pooled analysis, 22 participants] |  | NR |  |  |
| Lissiman 2014 | 1/1 | | <48 hours | 12 weeks | 1/1 | Duration of cold:  5.63 [as reported narratively, 73 participants] | - | NR |  |  |
| Science 2012 | 17 | | less than 48hours | until resolution of symptoms | 5/17 | Mean duration *Adults 7.12 (1.25) [calculated pooled mean, 185 participants] | 8/17 | Proportion of patients who were symptomatic [reverse: Proportion of patients with no symptom] Day 3: 14.2% [calculated, pooled analysis, 858 participants] |  |  |
|  |  | |  |  | 3/17 | *Children 7.0 (2.4) [ calculated pooled mean, 281 participants | 9/17 | Day 7: 52.9% [calculated, pooled analysis, 471 participants] |  |  |
| **Otitis media** | | | | | | | | | | |
| Venekamp 2015 | 13/13 | | NR | NR (1, 2-3,4-7,10-14, 2-4 weeks, 3 months) |  | NR | 6/13 | Proportion of patients recovered:  Day 1: 60% [as reported, pooled analysis, 685 participants. |  |  |
|  |  | |  |  |  |  | 7/13 | Days 2-3: 84% [as reported, pooled analysis, 1134 participants. |  |  |
|  |  | |  |  |  |  | 8/13 | Days 4-7: 76% [as reported, pooled analysis, 667 participants. |  |  |
|  |  | |  |  |  |  | 1/13 | Days 10-12: 78% [as reported pooled analysis, 139 participants] | | |
| **Laryngitis** |  | |  |  |  |  |  |  |  |  |
| Reveiz 2015 | 3/3 | | 3.6 days | Ranged from 1 and 2 weeks, up to 2 to 6 months (5, 8, 28) | 3/3 | Mean duration  5-28 days [as reported narratively, 351 participants | 1/3 | Clinical cure  **Fusafungine versus no treatment:**  Day 5 ± 1: 44.4 % [calculated, pooled analysis 45 participants] |  |  |
|  |  | |  |  |  |  | 1/3 | Day 8 ± 1: 78% [calculated, pooled analysis, 45 participants] |  |  |
|  |  | |  |  |  |  | 1/3 | Day 28 ± 2: 76% [calculated, pooled analysis, 38 participants] | | |
|  |  | |  |  |  |  | 1/3 | **Fusafungine + clarithromycin versus no treatment**  Day 5 ± 1: 44.4% [calculated, pooled analysis, 97 participants] | | |
|  |  | |  |  |  |  | 1/3 | Day 8 ± 1: 78% [calculated, pooled analysis, 97 participants] |  |  |
|  |  | |  |  |  |  | 1/3 | Day 28 ± 2: 57% [calculated, pooled analysis, 40 participants] | | |
|  |  | |  |  |  |  | 1/3 | **Erythromycin versus placebo [symptoms present, reversed for those with no symptoms**.  Day 7: 49% [calculated, pooled analysis, 99 participants] |  |  |
|  |  | |  |  |  |  | 1/3 | Day 14: 66.7% [calculated, pooled analysis, 99 participants. |  |  |
| **Rhinitis** |  | |  |  |  |  |  |  |  |  |
| ^24^Segboer 2019 | 13/13 | | NR | 4 weeks | - | NR | 1/13 | Symptom improvement  Week 6: 49% [ calculated, primary study report, 162 participants] |  |  |
|  |  | |  |  |  |  | 1/13 | Week 6: 39% [ calculated, primary study report, 32 participants] |  |  |
|  |  | |  |  |  |  | 1/13 | Week 4: 51% [ calculated, primary study report 25 participants] |  |  |
|  |  | |  |  |  |  | 1/13 | *Week 2: 17% [ calculated, primary study report, 24 adult participants] |  |  |
| **Conjunctivitis** | | | | | | | | | | |
| ^25^Sheikh 2012 | 11/11 | | <4 weeks | NR (2,5,6, 10) | - | NR | 6/11 | Remission rate: Clinical (cure)  Day 5: 30% [as reported pooled analysis, 1059 participants] | | |
|  |  | |  |  |  |  | 8/11 | Day 10: 41% [as reported, pooled analysis,1197 participants] | | |
|  |  | |  |  |  |  | 7/11 | **Microbiological remission (cure)**  Day 5: 54% [calculated, pooled analysis, 864 participants] | | |
|  |  | |  |  |  |  | 8/11 | Day 10: 62% [calculated, pooled analysis, 1197 participants] | |  |
| **Otitis media with Effusion** | | | | | | | | | | |
| Griffin 2011 | 16/16 | | NR | 2-6 weeks, >12 weeks | - | NR | 3/16 | cure or no cure [TBD]:  1. Antihistamine versus control  1-3 months: 48%, [calculated, pooled analysis, 109 participants] |  |  |
|  |  | |  |  |  |  | 3/16 | 2. Decongestant versus control  < 1 month: 71%, [calculated, pooled analysis, 113 participants | | |
|  |  | |  |  |  |  | 2/16 | 1-3 months: 61.5%, [calculated, pooled analysis, 83 participants | | |
|  |  | |  |  |  |  | 4/16 | 3. Antihistamine/decongestant combination versus control  1 month: 76% [calculated, pooled analysis, 444 participants] |  |  |
|  |  | |  |  |  |  | 3/16 | 1-3 months: 58% [calculated, pooled analysis for 158 participants] |  |  |
|  |  | |  |  |  |  | 2/16 | 3 months, 27% [calculated, pooled analysis for 119 participants] |  |  |
| ^26^Venekamp 2016 | 23/25 | | mean duration 10.6 weeks | 10-14 days, up to 4 weeks | - | - | 6/23 | Complete resolution]  Month 2 to 3: 25%, [calculated, pooled analysis, 227 participants] |  |  |
|  |  | |  |  |  |  | 14/23 | Complete resolution at fixed time post randomisation  Week 2 to 4: 20.3 % [calculated, pooled analysis, 870 participants] |  |  |
|  |  | |  |  |  |  | 5/23 | > 6 months, 26% [ calculated, pooled analysis, 275 participants] |  |  |
|  |  | |  |  |  |  |  | Complete resolution [end of treatment] |  |  |
|  |  | |  |  |  |  | 4/14 | Day 10-14: 17.2% [ calculated, pooled analysis, 285 participants |  |  |
|  |  | |  |  |  |  | 4/14 | Week 4: 19% [calculated, pooled analysis, 237 participants |  |  |
|  |  | |  |  |  |  | 2/14 | Month 3: 27% [calculated, pooled analysis, 60 participants |  |  |
|  |  | |  |  |  |  | 2.14 | Month 6: 15% [ calculated, pooled analysis, 80 participants |  |  |
| **Acute exacerbation of Chronic Obstructive Pulmonary Disease** | | | | | | | | | | |
| ^27^Vollenweider 2018 | 8/19 | | 7 to 1 months |  |  | Mean duration of symptoms | 1/1 | Cure  Day 5: 26% [ reported, primary study, 41 participants |  |  |
|  |  | |  |  | 1/1 | 12.8 days [reported, primary study,180 participants] | 1/1 | Day 8: 10% [ reported, primary study, 136 participants |  |  |
|  |  | |  |  | 1/1 | 13.5 days [reported, primary study, 30 participants] | 1/1 | Day 10: 60% [reported, primary study, 91 participants.  Day 20: 67.8% |  |  |
|  |  | |  |  |  |  | 1/1 | Day 21: 55% [reported, primary study, 99 participants] |  |  |
| **Otitis externa** | | | | | | | | | | |
| Kaushik 2010 | 2/19 | | NR | 3,5,7,10,21 | NR | NR | 1/2 | Cure  Day 10: 10% [as reported, narratively, 20 participants] |  |  |
| Rosenfeld 2006 | 3/20 | | 2-4 weeks | up to 21 days | - | NR | 2/3 | Clinical cure  ^28^Day 3-10: 15.2% [ calculated, pooled analysis, 46 participants |  |  |
|  |  | |  |  |  |  |  |  |  |  |
| **Bronchiolitis** | | | | | | | | | |  |
| ^29^Gadomski 2014 | 10/30 | | NR | 90-120min | 2/10 | Time to resolution: 6.6 [3.07] [calculated, pooled analysis, 135 participants] | 2/10 | Improvement in clinical score  Day X: 84% [ calculated, pooled analysis, 77 participants] |  |  |
| **Abscess** |  | |  |  |  |  |  |  |  |  |
| ^30^Bowen 2017 | 4/15 | | NR | 3-30days | - | - | 1/1 | Clinical cure  **Drained abscess > 2cm [uncomplicated]**  Day 7: 73.6%, [narrative synthesis, 1247 participant] |  |  |
|  |  | |  |  |  |  | 1/1 | **Abscess > 5cm**  ^31^Day 7: 74% [as reported narratively, 102 participants] |  |  |
|  |  | |  |  |  |  | 1/1 | Day 10-14: 68.9% [as reported, narratively, 786 participants] |  |  |
|  |  | |  |  |  |  | 1/1 | Day 10-14: 95% [as reported, narratively, 161 participants] [Duong]: |  |  |
| ^32^Wang 2018 | 8/14 | | NR | 7-90 days | - | NR | 8/8 | Treatment failure [reversed for treatment success]  Day 28: 85% [calculated, narrative report, summary tables, 1211 participants] |  |  |
|  |  | |  |  |  |  |  |  |  |  |

N: Overall number of studies included in the original review n: Number of studies contributing to the reported natural history outcome. X: number of studies contributing data to the mean duration. Y: Number of studies contributing data to the proportion of participants. NR= not reported, NA: not applicable

^1^ The total number of reporting is more than the total number of included reviews (n=40), as a few reviews (n=2) have reported on more than one condition.

^2,3^ The outcomes are reported for the placebo or no treatment arm. Outcome data were extracted from intext information, forest plots, tables, or graphs.

^4^ The outcome data were reported as the proportion of participants with cough symptoms-we calculated outcome data from those with symptoms at the specified timepoint.

^5^ Day 14 data obtained from the reverse of patients who may be no better at 14 days as narratively reported in the abstract

^6^ Contributed data to other conditions, including cough, sore throat, otitis media, common cold, croup, and bronchiolitis.

^7^ The outcome data were reported as the proportion of participants with sore throat remaining at follow-up; we calculated the proportion with symptom resolution from the number with symptoms at time point.

^8^ Contributed data to other conditions, including both otitis media and otitis media with effusion.

^9^ The proportion of participants symptom free at day 21 is an estimated proportion presented narratively in the review.

^10^ As reported in the review, the proportion of participants symptom free at week 6 will be much lower (36%) if the review had accounted for crossover. The review provided details for the participant's cure and the number with symptom improvements.

^11^ As reported in the review, concomitant treatment (such as povidone-iodine shampoo, hexachlorophene soap, and Castile bath soap), was allowed in some included studies, although not specified.

^12^ Included trials in the review permitted the use of antibiotics in the placebo group except in one study. We calculated the outcome data from the one study for which no antibiotic was allowed. The outcome data was reported as participants with cough. We calculated the proportion with symptoms resolution from the number with symptoms.

^13^ Outcome reported for patients with subacute cough.

^14^ No timepoint was provided for the outcome data. We assumed the timepoint from one trial, Kammerich 2017, which was the only included study that provided a timepoint for assessment.

^15^ Outcome reported from only one study in the review. Review reported the median duration of pain.

^16^ The outcome data on day 3 was reported as the proportion of participants with symptoms; we calculated the proportion with symptoms resolution from the number with symptoms.

^17^ The mean time to symptom resolution was reported as a range from 3 of the included studies in the review.

^18^ The review did not find any eligible studies.

^19^ We used the length of time to clinical success for the proportion data point.

^20^ The outcome data were reported as an improvement in symptoms score; we calculated the proportions from the forest plots with proportion data.

^21^ The outcome data on day 7 was reported as the proportion of participants with symptoms; we calculated the proportion symptoms resolution from the number with symptoms.

^22^ We calculated the pooled mean from the 2 eligible studies.

^23^ The outcome data were reported as the proportion of participants with persistent symptoms. We calculated the proportion with symptom resolution from the number with symptoms.

^24^ We extracted outcome data from included primary studies that provided proportions of patients with symptoms improvement or cure.

^25^ The review reported a higher proportion of participants with microbial remission than those with clinical remission.

^26^ The review reported that most trials were performed in secondary care. 52% of placebo participants received a true placebo; others received treatment of unproven efficacy.

^27^Outcome data reported from individual studies.

^28^Outcome calculated from review pooled analysis.

^29^ The time to resolution of symptoms was calculated from the reported pooled mean of two studies that provided data for outpatient outcomes. We used the time point to the resolution of symptoms from the pooled mean.

^30^ The outcome data were presented narratively from individual studies.

^31^ The outcome data were reported as the proportion of participants with treatment failure. We calculated the proportion from symptom resolution from treatment failure participants.

^32^ We obtained outcome data from 8 trials that compared antibiotics to no antibiotics. Outcome data was reported as treatment failure; we calculated the proportion symptom resolution from the number that failed to improve.
